# Recent polyploidization events in three *Saccharum* founding species

**DOI:** 10.1111/pbi.12962

**Published:** 2018-07-24

**Authors:** Jisen Zhang, Qing Zhang, Leiting Li, Haibao Tang, Qiong Zhang, Yang Chen, Jie Arrow, Xingtan Zhang, Aiqin Wang, Chenyong Miao, Ray Ming

**Affiliations:** ^1^ FAFU and UIUC Joint Center for Genomics and Biotechnology Key Laboratory of Sugarcane Biology and Genetic Breeding Ministry of Agriculture Fujian Agriculture and Forestry University Fuzhou Fujian China; ^2^ Fujian Provincial Key Laboratory of Haixia Applied Plant Systems Biology, and Key Laboratory of Genetics Breeding and Multiple Utilization of Corps Ministry of Education Fujian Agriculture and Forestry University Fuzhou Fujian China; ^3^ College of Life Sciences Fujian Normal University Fuzhou China; ^4^ College of Life Sciences Fujian Agriculture and Forestry University Fuzhou Fujian China; ^5^ College of Horticulture Nanjing Agricultural University Nanjing China; ^6^ Key Laboratory of Plant Germplasm Enhancement and Specialty Agriculture Wuhan Botanical Garden Chinese Academy of Sciences Wuhan China; ^7^ Department of Plant Biology University of Illinois at Urbana‐Champaign Urbana IL USA; ^8^ State Key Lab for Conservation and Utilization of Subtropical Agro‐biological Resources Guangxi University Nanning China

**Keywords:** genetic map, polyploidy, *Saccharum*, single‐dose marker, transcriptome sequencing

## Abstract

The complexity of polyploid *Saccharum* genomes hindered progress of genome research and crop improvement in sugarcane. To understand their genome structure, transcriptomes of 59 F_1_ individuals derived from *S. officinarum* LA Purple and *S. robustum* Molokai 5829 (2*n* = 80, *x *=* *10 for both) were sequenced, yielding 11 157 and 8998 SNPs and 83 and 105 linkage groups, respectively. Most markers in each linkage group aligned to single sorghum chromosome. However, 71 interchromosomal rearrangements were detected between sorghum and *S. officinarum* or *S. robustum*, and 24 (33.8%) of them were shared between *S. officinarum* and *S. robustum*, indicating their occurrence before the speciation event that separated these two species. More than 2000 gene pairs from *S. spontaneum*,* S. officinarum* and *S. robustum* were analysed to estimate their divergence time. *Saccharum officinarum* and *S. robustum* diverged about 385 thousand years ago, and the whole‐genome duplication events occurred after the speciation event because of shared interchromosomal rearrangements. The ancestor of these two species diverged from *S. spontaneum* about 769 thousand years ago, and the reduction in basic chromosome number from 10 to 8 in *S. spontaneum* occurred after the speciation event but before the two rounds of whole‐genome duplication. Our results proved that *S. officinarum* is a legitimate species in its own right and not a selection from *S. robustum* during the domestication process in the past 10 000 years. Our findings rejected a long‐standing hypothesis and clarified the timing of speciation and whole‐genome duplication events in *Saccharum*.

## Introduction

Sugarcane is the most economically important sugar crop, supplying 80% sugar consumed worldwide. Given the recent demand for alternatives to fossil fuels, it is one of the most productive, first‐generation liquid biofuel feedstocks (Kole, 2010). Sugarcane exemplifies an extreme case of autopolyploidy making fundamental genetic studies comparatively more complex than those with other diploid crops (Henry *et al*., 2010). There are six species in the genus *Saccharum*, including *S. spontaneum*,* S. robustum*,* S. officinarum*,* S. barberi*,* S. sinense* and *S. edule*. *Saccharum spontaneum* and *S. robustum* are wild species with basic chromosome number *x *=* *8 and *x *=* *10, respectively (D'Hont *et al*., 1996; D'hont *et al*., 1998; Ha *et al*., 1999). Chromosome numbers vary widely in the two wild species with 2*n* = 40 to 128 in *S. spontaneum* and 2*n* = 60–200 in *S. robustum* (Irvine, 1999). *Saccharum officinarum* is a domesticated high biomass and high sugar content species with 2*n* = 8*x* = 80 and is thought to be derived from the wild species *S. robustum* (mainly 2*n* = 60, 80) as shown by the close relationship of these two species (D'Hont *et al*., 1993; Lu *et al*., 1994; Schenck *et al*., 2004). *Saccharum barberi* and *S. sinense* are interspecific hybrids between *S. officinarum* and *S. spontaneu*m (D'Hont *et al*., 2002), and *S. edule* could be an interspecific or intergeneric hybrid between either *S. officinarum* or *S. robustum* with a species in the *Saccharum* complex (Roach and Daniels, 1987). Hence, *S. spontaneum, S. robustum* and *S. officinarum* are the founding species of *Saccharum*.

Modern sugarcane cultivars are hybrids made by sugarcane breeders starting in the early 1900s to complement the high sucrose but often disease susceptible *S. officinarum* with the disease resistance and stress tolerance traits of *S. spontanuem*. As a consequence, *Saccharum* cultivars exhibit an exceedingly complex interspecific aneupolyploid genome with 100–130 chromosomes, of which 70%–80% are from *S. officinarum*, 10%–20% from *S. spontaneum* and about 10% from interspecific recombination. Thus, the genome structure of sugarcane cultivars is characterized by both homologous interspecific and intraspecific chromosomes (D'Hont, 2005). The evidence from genomics showed that divergence between *S. spontaneum* and *S. officinarum* occurred 1.5–2 million years ago (mya) (Jannoo *et al*., 2007). So far, there is no convincing published information regarding the genomic relationship between *S. robustum* with *S. spontanuem* and *S. officinarum*. The remaining species in *Saccharum* are interspecific hybrids of the three aforementioned species and are sometimes classed as a horticultural group (Aitken and McNeil, 2010).

The *Saccharum* is particularly noted for polyploidy with octopolyploid at the highest frequency in *S. spontaneum*,* S. officinarum* and *S. robustom* (Irvine, 1999). Previous studies based on comparison of partial genetic maps to sorghum indicated that two rounds of whole‐genome duplication occurred in *S. officinarum* after the divergence of *Saccharum* and *Sorghum* (Ming *et al*., 1998). Recently, *Saccharum* was suggested to share one round of whole‐genome duplication with *Miscanthus* after their divergence from the sorghum lineage about 3.8–4.6 mya (Kim *et al*., 2014). The sequence of the sugarcane genome remains unavailable due to the high degree of polyploidy and heterozygosity. High levels of gene retention rate and conservation of gene structure were detected among seven homologous haplotypes in sugarcane cultivar R570, *S. spontaneum* AP85‐441 and *S. officinarum* LA Purple, and most of the genes in the haplotypes were predicted to be functional (Garsmeur *et al*., 2011). However, transposable elements evolved at a much higher rate and showed no collinearity among haplotypes (Garsmeur *et al*., 2011). *Saccharum* shared common ancestors with its close diploid species sorghum about 6–8 mya (Jannoo *et al*., 2007; Ming *et al*., 2005; Wang *et al*., 2010). The studies of comparative genomics based on bacterial artificial chromosomes (BACs) have shown that *Saccharum* and sorghum share a strong collinearity and contain few chromosomal rearrangements (Dufour *et al*., 1997; Guimarães *et al*., 1997; Jannoo *et al*., 2007; Ming *et al*., 1998; Wang *et al*., 2010), suggesting polyploidization of *Saccharum* did not cause significant genome reconstruction. In contrast, numerous small‐scale genome rearrangements were found between sorghum and sugarcane (Wang *et al*., 2010). In this decade, more than a dozen genetic linkage maps for *Saccharum* were developed for the progenitor species and important cultivars using pseudo‐test crosses and pseudo‐F_2_ mapping populations (Alwala *et al*., 2010; Pastina *et al*., 2010). At present, the most comprehensive map of sugarcane is made of >2000 marker loci in a segregating population of ~200 individuals and spans >9000 cM (Aitken *et al*., 2014). However, while this genetic map has cumulatively furthered the understanding of the complexity of the sugarcane genome composition. The genomes of *Saccharum* species still lack adequate understanding as most genetic maps were derived from *Saccharum* hybrids and had low coverage for their genomes.

In this study, we addressed the challenge for SNPs genetic mapping caused by the complex genomes in *Saccharum*. We sequenced leaf transcriptomes of the three founding *Saccharum* species and a segregating F_1_ population derived from *S. officinarum* LA Purple (*x *=* *10, 2*n* = 80) and *S. robustum* Molokai 6081 (*x *=* *10, 2*n *= 80). We analysed interchromosomal rearrangements between sorghum and *Saccharum*, intrachromosomal rearrangements among *Saccharum* species and the divergence time among three founding species of *Saccharum*. The aims of this project were (i) to understand the genome structure and organization of *Saccharum* genomes, (ii) to estimate the divergence time of the three founding species of the genus *Saccharum* and (iii) to present a strategy for dissecting large complex autopolyploidy genomes.

## Results

### The development of single‐dose SNPs for genetic mapping

To develop single‐dose SNPs in coding sequences of sugarcane genomes, we sequenced the transcriptomes of *S. officinarum* (LA Purple) and 59 F_1_ progenies from the cross of between *S. officinarum* (LA Purple) and *S. robustum* (Molokai 5829). Considering the high level of polyploidy in *Saccharum*, a large quantity of reads, in total over 190 Gbp with an average of 3.22 Gbp for each library, was generated by RNA‐Seq with Illumina HiSeq 2000 (Table 1). The alignment results showed that 68% of raw reads were aligned to the reference's gene models of sorghum. The distribution of aligned reads showed sequence depth within SPS gene presented parallel pattern among the testing six libraries (Figure S1), showing a similar depth coverage distributed among individual libraries.

**Table 1:** Summary of RNA‐Seq data (bp) from the segregating F_1_ mapping population

	Read sequences	Aligned	Gapped alignment	Quality filter	Homopolymer filter
Total	1 903 710 934	1 295 645 376	234 936 140	3 566 099	43 469
Average	32 266 287	21 960 091	3 981 968	60 442	737

We used the ratios of minor and major allele frequency within the 1 : 6 and 1 : 30 window to screen the single dosage SNPs by merging aligned sequences of progenies. We analysed the correlations between cumulative SNP number and the minor nucleotide depth. Our results showed that the cumulative numbers of SNPs ranged from 2680 to 62 398, which is correlated with descent depth, ranging from 24 to 198 for minor nucleotide depth (Figure S2). Furthermore, we analysed the distribution of single‐dose SNPs based on plant numbers and segregation ratios (Figures S3a and 3b). According to screening criteria of progeny segregation ratio with a range of 0.3–0.7 and a minimum of 39 individuals with less than 10% missing data, a total of 20 842 SNP and 11 158 SNP markers were generated from *S. officinarum* (LA Purple) and *S. robustum* (Molokai 6081), respectively.

### High‐density genetic mapping of *Saccharum* species based on transcriptome‐derived single‐dose SNPs

Linkage map of *S. officinarum*: The 20 842 SNP markers from *S. officinarum* were sorted into 8094 marker bins of co‐segregating markers, each bin containing 1–22 markers. Of the 8094 bins, 4629 were mapped to 83 linkage groups in the highest confidence map (Tables 2 and S1). These marker bins correspond to 14 050 SNP markers of the total 20 842 found. The total genetic map length of *S. officinarum* is 7096.5 cM, with a density of 1.53 markers/cM. Based on sorghum gene models, 6832 unigenes were mapped to the highest confidence maps.

**Table 2:** Linkage mapping of SNP markers in two *Saccharum* species

*Saccharum* species	No. of markers	No. of bins	No. of markers in mapped bins	No. of LGs	Map length (cM)	Density (/cM)
*S. officinarum*	20 842	8094	4628	83	7096.5	1.53
*S. robustum*	11 158	5619	3078	105	6742.0	2.19

Two RNA‐seq maps were created, one for each species. *Saccharum robustum* yielded more linkage groups with low density due to a lack of recombination events observed between a lower number of markers.

Linkage map of *S. robustum*: The 11 158 SNP markers were sorted into 5620 marker bins with co‐segregating markers**.** Of the 5620 marker bins, 3078 were used in the highest confidence map with 105 linkage groups with average of 52 markers (Tables 2 and S2). The total map length of *S. robustum* is 6742.0 cM with a density of 1.07 markers/cM. Based on sorghum gene models, there are 4371 unigenes mapped to the highest confidence map.

In addition, in the linkage map of *S. officinarum* and *S. spontaneum*, 10 and five unassembled scaffolds of sorghum were mapped, respectively (Table 3).

**Table 3:** The distribution of SNP markers in corresponding sorghum chromosomes

Sorghum chromosome	No. of genes	*Saccharum officinarum*			*Saccharum robustum*		
		LG	Bin	Detected genes	LG	Bin	Detected genes
1	5572	14	739	852	15	569	1587
2	4483	9	563	696	13	341	1068
3	4565	10	640	795	15	481	1261
4	3714	8	518	644	15	463	1115
5	2554	6	281	195	7	154	235
6	2981	4	297	452	5	172	360
7	2423	9	402	350	6	204	611
8	2113	7	275	226	6	132	237
9	2692	9	495	463	10	281	668
10	2913	7	409	426	13	276	740
Other			10			5	
Total	34 008	83	4629		105	3078	

### Conservation and collinearity in the genome structure of *Saccharum* and its close diploid relative sorghum

**Figure 1:**
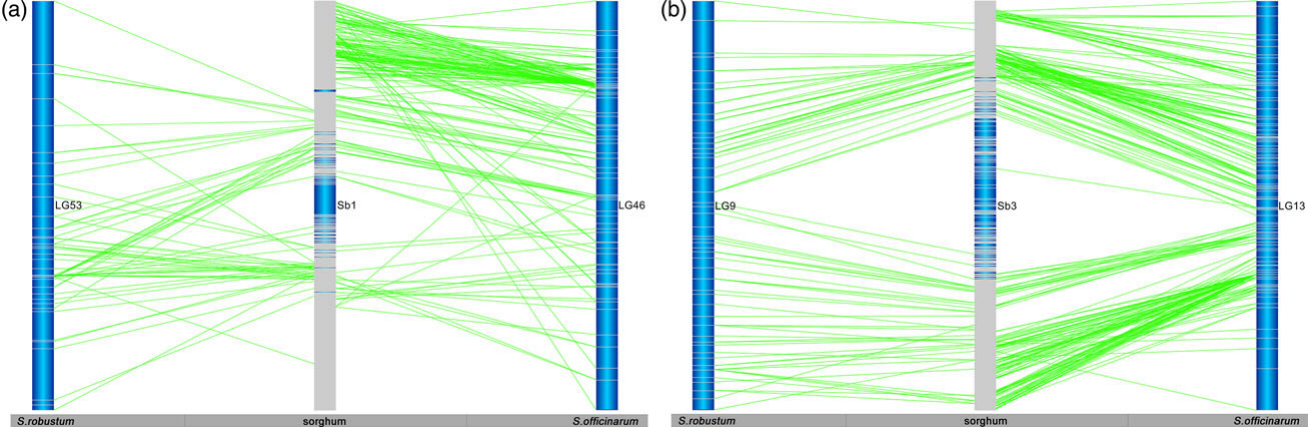
The collinearities among sorghum, *Saccharum officinarum* and *Saccharum robustum. Notes*: ?a) The collinearities between Sb1 and *S. officinarum* LG53 and between Sb1 and *S. robustum* LG46. (b) The collinearities between Sb3 and *S. officinarum* LG9 and between Sb3 and *S. robustum* LG13. The collinearities between sorghum and *Saccharum* and between *S. officinarum* and *S. robustum* were shown by the alignment of *Saccharum* homologous linkage groups to sorghum chromosome. Dark blue colour is the background of the chromosomes/LGs. The grey lines are the position of genetic markers/genes. The dark blue colour in the centre of the sorghum chromosome indicated that low density of genes existed in the regions of chromosome. The bars on the sugarcane LG represent markers from Table S1. These are aligned using the BLASTN algorithm (*P* < e^−20^) and the position indicated by lines to the other chromosomes. See Figure S5 for more information.

Based on the sorghum reference genome, the mapped gene models of sorghum provide 4629 and 3078 anchor points to the sorghum genome in *S. officinarum* and *S. robustum*, respectively (Table 2). We used the mapped gene models of sorghum for subsequent analysis of the two *Saccharum* genomes. As expected, few markers from both *Saccharum* species were aligned to potential sorghum centromeric regions due to low gene density and gene expression surrounding the centromeric heterochromatic regions (Figure S4). Comparing sugarcane LGs with sorghum chromosomes allowed the observation of the extensive collinearity among homologous chromosomes (Figures 1 and S5). Based on the homology between the genic markers and sorghum gene models, all LGs from both *Saccharum* species corresponded to 10 sorghum chromosomes, referred to as homologous groups 1–10 (HGs 1–10). Of the combined 4629 markers in 83 LGs of *S. officinarum*, 4018 (86.8%) aligned to orthologous sorghum chromosomes. Of the combined 3078 markers in 105 LGs of *S. robustum*, 2798 (90.9%) aligned to orthologous sorghum chromosomes.

Of the combined 4629 markers in 83 LGs of *S. officinarum*, 4018 (86.8%) aligned to orthologous sorghum chromosomes. Among the 83 LGs in *S. officinarum*, 12% (10) LGs were aligned to orthologous sorghum chromosomes with 100% markers and the remaining 73 LGs with majority of markers aligned to orthologous sorghum chromosomes, ranging from 43.7% to 98.6% (Table S3). Consistently, majority of markers in each HG aligned to orthologous sorghum chromosomes, ranging from 76.0% to 95.5%. However, three of *S. officinarum* HGs (HG5, 6 and 7) do not have any LG with 100% aligned to their orthologous sorghum chromosomes (Table S3). Interchromosomal rearrangements were also observed in all of HGs in *S. officinarum*, for example LG4, 18, 23, 40 and 48 in HG1; LG69 in HG2; LG2, 12 and 39 in HG3; LG1 and LG3 in HG4; LG17 and LG66 in HG5; LG20 in HG6; LG34 in HG7; LG26 and LG31 in HG8; LG47 in HG9; and LG6 in HG10 (Table S3). These results demonstrated that *S. officinarum* HGs shared high homology to their orthologous sorghum chromosomes, although interchromosomal rearrangements did occur.

Of the combined 3078 markers in 105 LGs of *S. robustum*, 2798 (90.9%) aligned to orthologous sorghum chromosomes. Among the 105 LGs in *S. robustum*, 24% (25) LGs were aligned to orthologous sorghum chromosomes with 100% markers, whereas the remaining 80 LGs with majority of markers aligned to orthologous sorghum chromosomes, ranging from 57.4% to 97.9% (Table S3). Similarly, majority of markers in each HG aligned to orthologous sorghum chromosomes, ranging from 81.3% to 94.5%; and each of the HGs has at least one LG with 100% aligned to their orthologous sorghum chromosomes (Table S3). Interchromosomal rearrangements were also observed in each of HGs in *S. robstum*, for example LG28 in HG1; LG69 in HG2; LG6 in HG3; LG22 in HG4; LG70 in HG5; LG55 in HG6; LG7 in HG7; LG41 in HG8; LG8 in HG9; and LG20 in HG10 (Table S3). These results demonstrated that *S. robustum* HGs shared strong homology to their orthologous sorghum chromosomes.

Based on the distribution of mapped bin markers in sorghum chromosomes, we were able to observe the homologous haplotype in *Saccharum* species (Table 4 and Figure S4). In the 80 LGs of *S. officinarum*, the HGs had a range from 5 to 14 LGs, of them, HG1, HG2, HG4, HG7 and HG9 had 14, 10, 9, 11 and 9 LGs, respectively. In *S. robustum*, the 10 HGs have five to 15 LGs (Table 3). We further analysed the distributions of homologous genes from the *Saccharum* LGs in sorghum chromosomes (Figure S4). In all HGs, there were some LGs that only covered partial chromosomes when we aligned the mapped markers onto their sorghum homologous chromosome. This could explain why some HGs have more than eight LGs and thus suggested that the expression levels of the homologous chromosomes were the result of bias in the polyploid *Saccharum* since SNP discovery depends on the sequencing depth of homologous chromosomes.

**Table 4:** The distribution of genetic map bin markers of the two *Saccharum* species in corresponding sorghum chromosome

*Saccharum* HG	*Saccharum* species	Sb01	Sb02	Sb03	Sb04	Sb05	Sb06	Sb07	Sb08	Sb09	Sb10	The other	Total	Congruous loci (%)
HG1	*S. robustum*	**531**	5	5	3	9	2		2	2	3	1	563	94.3%
	*S. officinarum*	**673**	2	58	10	30	13	1	6	1	7	2	803	83.8%
HG2	*S. robustum*	1	**307**	11	2	1	1	2	1	1	2		329	93.3%
	*S. officinarum*	9	**501**	8	5	1	5	1	4	17	2		553	90.6%
HG3	*S. robustum*	8	2	**450**	4	3	1	11	6	1	3	1	490	91.8%
	*S. officinarum*	21	22	**545**	9	20	11	4		38	44	3	717	76.0%
HG4	*S. robustum*	3	3	4	**440**	1	1	11	1	1	3		468	94.0%
	*S. officinarum*	4	3	12	**448**	2	7	6		2	8	1	493	90.9%
HG5	*S. robustum*	1	1		5	**133**	1	5	1		1	3	151	88.1%
	*S. officinarum*	3	10			**209**	1	4	6	4	5	2	244	85.7%
HG6	*S. robustum*	4		3	2		**155**						164	94.5%
	*S. officinarum*	5	1	2		2	**255**			1	1		267	95.5%
HG7	*S. robustum*	1	1				4	**159**	2	3	1		171	93.0%
	*S. officinarum*	10		8		1	3	**379**	4	23	4	2	434	87.3%
HG8	*S. robustum*	2	1	4	1	1	1	1	**115**	2			128	89.8%
	*S. officinarum*	10	11	3	5	9			**252**	3	20		313	80.5%
HG9	*S. robustum*	18	20	3		2		4	2	**270**	13		332	81.3%
	*S. officinarum*	2	4	2	39	4		2	3	**399**	4		459	86.9%
HG10	*S. robustum*		1	1	6	4	6	11	2	1	**250**		282	88.7%
	*S. officinarum*	2	9	2	2	3	2	5		7	**314**		346	90.8%
Summary	*S. robustum*	569	341	481	463	154	172	204	132	281	276	5	3078	90.9%
	*S. officinarum*	739	563	640	518	281	297	402	275	495	409	10	4629	86.8%

The majority bin markers of each HG of *Saccharum* species corresponding sorghum chromosome were labeled with bold.

### Inter‐ and intrachromosomal rearrangements between *Saccharum* and sorghum

**Figure 2:**
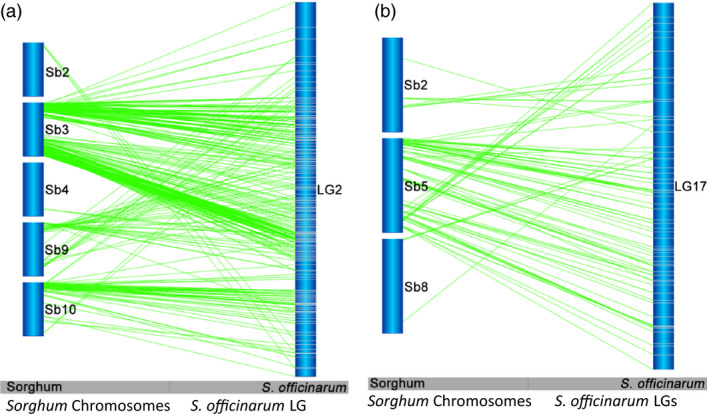
Interchromosomal rearrangements between *Saccharum* and sorghum chromosomes. The typical interchromosomal rearrangements between *Saccharum* and sorghum chromosomes were selected to present. (a) Interchromosomal rearrangements between *Saccharum* and sorghum chromosomes in *S. officinarum* LG2. (b) Interchromosomal rearrangements between *Saccharum* and sorghum chromosomes in *S. officinarum* LG17. See Figure S6 for more information.

**Figure 3:**
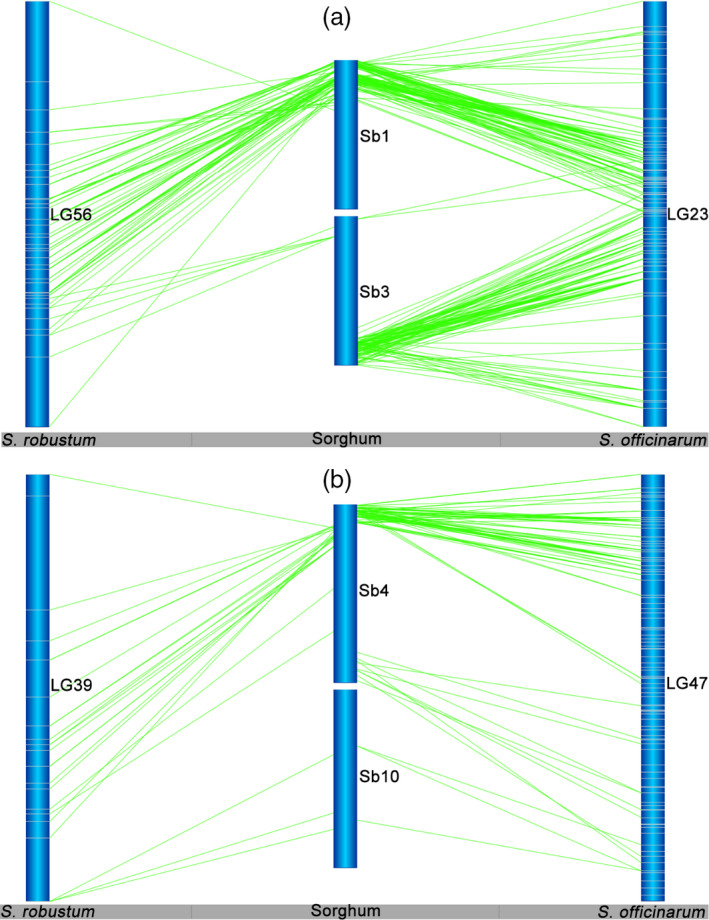
Interchromosomal rearrangements between *Saccharum* and sorghum chromosomes sharing in *S. officinarum* and *S. robustum*. (a) Interchromosomal rearrangements between Sb1 and Sb3 sharing in S. *officinarum* and *S. robustum*. (b) Interchromosomal rearrangements between Sb4 and Sb10 sharing in *S. officinarum* and *S. robustum*. *Notes*: See all 36 interchromosomal rearrangements sharing in *S. officinarum* and *S. robustum* in Figure S7. Selected representative of different types in these figures. These common interchromosomal rearrangements were assumed to occur before the divergence of *S. officinarum* and *S. robustum*, suggesting that the two rounds of whole‐genome duplications occurred after their speciation event.

Interchromosomal rearrangements between sorghum chromosomes and *Saccharum* LGs were observed as shown by a single *Saccharum* LG aligned to two or more sorghum chromosomes (Figures 2 and 3). Of the 83 LGs in *S. officinarum*, 39 (47.0%) LGs were found to have interchromosomal arrangements with a minimum of three syntenic markers aligned to another sorghum chromosome or chromosomes. Among these 39 LGs, one aligned to five sorghum chromosomes, two aligned to four sorghum chromosomes, seven aligned to three sorghum chromosomes and the remaining 29 aligned to two sorghum chromosomes. Fifty‐three recombination events were observed in the 39 LGs. Among the 53 interchromosomal recombination events, 14 (26.4%) were shared with *S. robustum*. Of the105 LGs in *S. robustum*, 17(16.2%) LGs were observed to have interchromosomal arrangements, and each LG was aligned to two sorghum chromosomes besides LG7 that was aligned to three sorghum chromosomes. Eighteen recombination events were observed in the 17 LGs. Among these 18 interchromosomal rearrangements, 10 (55.6%) were shared with *S. officinarum*. The different number of LGs (14 in *S. officinarum* and 10 in *S. robustum*) detecting shared interchromosomal rearrangements is caused by multiple LGs detecting one interchromosomal rearrangement or one LG detecting two or more interchromosomal rearrangements as summarized in Table S4. For example, the interchromosomal rearrangement involved two sorghum chromosomes Sb1 + Sb5 was detected in one LG (LG27) in *S. robustum* and two LGs (LG40 and LG15) in *S. officinarum*, hence different numbers of LGs in shared interchromosomal rearrangements. Of the combined 71 interchromosomal rearrangements in *S. officinarum* and *S. robustum*, 24 (33.8%) were conserved and shared in these two species (Table S4 and Figure S6). Moreover, four *S. robustum* LGs (LG27, LG56, LG6 and LG8) have 2–4 common interchromosomal arrangements in *S. officinarum*, while two *S. officinarum* LGs, LG2 and LG12, have two and five common interchromosomal arrangements in *S. robustum* (Table S4).

**Figure 4:**
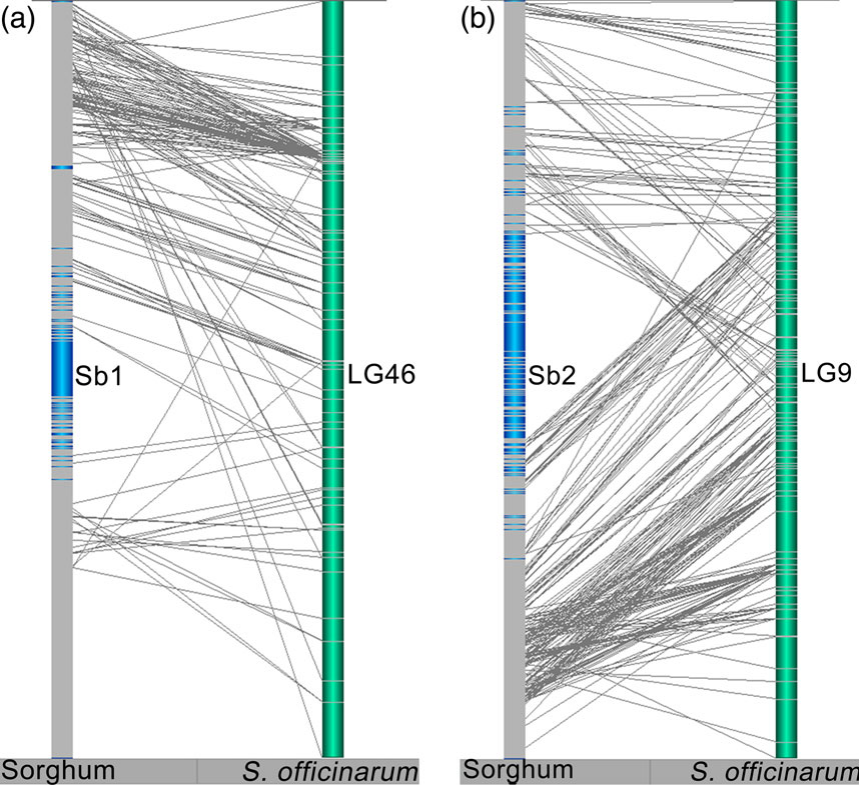
Intrachromosomal rearrangements between *Saccharum* and sorghum chromosomes. Intrachromosomal rearrangements between *Saccharum* and sorghum chromosomes were observed to be existed from both of *S. officinarum* and *S. robustum* (see more information in Figure S6). Inversion between *Saccharum* and sorghum was presented in HG1 for LG46 in *S. officinarum* (a). Expansion between *Saccharum* and sorghum was existed in HG2 for LG9 in *S. officinarum* (b).

**Figure 5:**
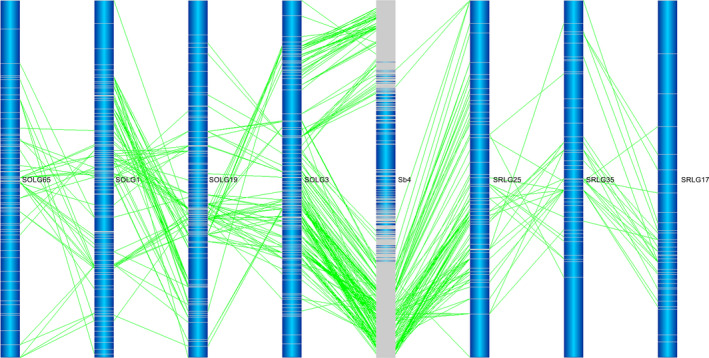
Intrachromosomal rearrangements of homologous group among *Saccharum* chromosomes. *Notes*: See Figure S8 for the 10 HGs of *S. officinarum* and *S. robustum*. Intrachromosomal arrangements were commonly existed among the LGs of each homologous groups, suggesting that the homologous chromosomes had genome structure variations in *Saccharum*.

Intrachromosomal rearrangements between *Saccharum* and sorghum chromosomes were prevalent in linkage groups from both *S. officinarum* and *S. robustum* (Figures 4 and S7). For example, several inversions between *Saccharum* and sorghum were presented in HG1 for LG46 in *S. officinarum* and LG27 in *S. robustum* (Figures 4a and S7), and in HG7 for LG58 in *S. officinarum* and LG7 in *S. robustum* (Figure S7); expansion between *Saccharum* and sorghum was existed in HG2 for LG9 in *S. officinarum* and LG97 in *S. robustum* (Figures 4b and S7), and in HG3 for LG2 in *S. officinarum* and LG9 in *S. robustum* (Figure S7). Moreover, intrachromosomal arrangements were detected among the LGs of each homologous groups (Figures 5 and S8), suggesting that the homologous chromosomes had genome structure variations in *Saccharum*.

### Divergence time between *Saccharum* and *Saccharum*–sorghum

We conducted RNA‐Seq analyses using the Illumina Hiseq2000 from five different tissues (leaf roll, leaf, top stalk, middle stalk and bottom stalk) of *S. officinarum* (LA Purple), *S. robustum* (Molokai 6081) and *S. spontaneum* (SES 208). A total of 459.75 million reads were generated from 15 RNA‐Seq libraries, which translates to an average of 30.65 million reads for each tissue of the species. The assembled contigs were aligned against sorghum gene models (Phytozome version 8.0). A total of 9150 contigs of *S. officinarum*, 59 098 contigs of *S. robustum* and 36 350 contigs of *S. spontaneum* matched high‐scoring segment pairs (HSPs), and those exceeding 50% of the length of the sorghum orthologs were retained for further analyses.

**Figure 6:**
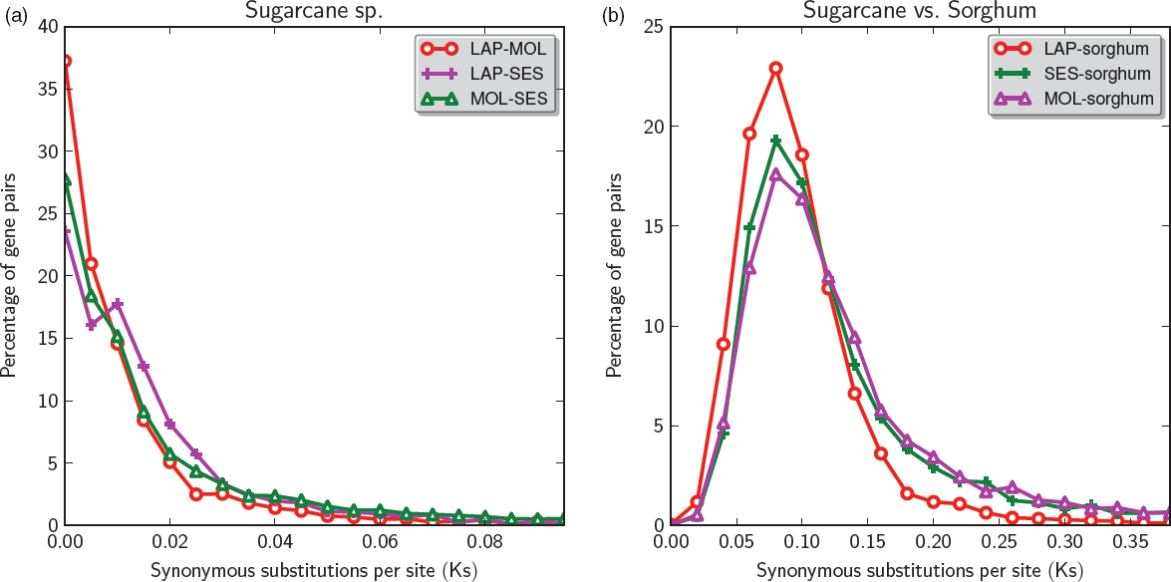
*K*s distribution between (a) *Saccharum* species and (b) *Saccharum*–sorghum. Among the three pairwise comparisons, *S. officinarum* (LA Purple) and *S. robustum* (Molokai 6081) have the smallest median *K*s value at 0.005, corresponding to a divergence time of 385 thousand years ago, while *S. robustum* (Molokai 6081) and *S. spontaneum* (SES 208) have the highest median *K*s value at 0.009, a divergence time of 692 thousand years ago. *S. officinarum* (LA Purple) and *S. spontaneum* (SES 208) have *K*s value at 0.010, a divergence time of 769 thousand years ago, close to the divergence time between *S. robustum* and *S. spontaneum*.

We estimated the divergence time between *Saccharum* species and between sugarcane–sorghum based on *K*s values (substitutions in synonymous sites) of more than 2000 gene pairs among three species (Table 5), assuming a generic grass substitution rate (Gaut *et al*., 1996). The pairwise *K*s between the two sugarcane species are all very close to zero, suggesting recent divergence among the three species (Figure 6 and Table 5). Among the three pairwise comparisons, *S. officinarum* (LA Purple) and *S. robustum* (Molokai 6081) have the smallest median *K*s value at 0.005, corresponding to a divergence time of 385 thousand years ago, while *S. robustum* (Molokai 6081) and *S. spontaneum* (SES 208) have the highest median *K*s value at 0.009, a divergence time of 692 thousand years ago. *Saccharum officinarum* (LA Purple) and *S. spontaneum* (SES 208) have *K*s value at 0.010, a divergence time of 769 thousand years ago, close to the divergence time between *S. robustum* and *S. spontaneum*. The *K*s values of the three sugarcane species and the best matching sorghum gene range from 0.087 to 0.106 (6.692 to 8.154 mya), similar to previous estimates based on a smaller set of sugarcane genes (Wang *et al*., 2010).

**Table 5:** Divergence time among *Saccharum* species

*Saccharum* species	Median *K*s	Gene pairs used	Divergence time (mya)
*S. officinarum*/*S. robustum*	0.005	2245	0.386
*S. officinarum/S. spontaneum*	0.010	2085	0.769
*S. spontaneum/S. robustum*	0.009	5171	0.692
*S. officinarum*/*Sorghum*	0.087	4050	6.692
*S. robustum*/*Sorghum*	0.106	22 378	8.154
*S. spontaneum*/*Sorghum*	0.101	14 329	7.779

## Discussion

The 24 interchromosomal rearrangements shared by *S. officinarum* and *S. robustum* accounted for 26.4% (14 of 53) in *S. officinarum* and 55.6% (10 of 18) in *S. robustum*. This is a vital piece of evidence supporting that the common ancestor of *S. officinarum* and *S. robustum* was a diploid about 385 thousand years ago, because of their shared interchromosomal rearrangements. The two rounds of WGD occurred less than 385 thousand years ago via autopolyploidization because there was hardly any preferential paring among their chromosomes as one would be expected if allopolyploidization occurred (Ming *et al*., 1998). It was suggested before that *Saccharum* and *Miscanthus* shared allopolyploid event before the divergence of these two genera about 3.8–4.6 mya (Kim *et al*., 2014). If true, it is unlikely to have 26.4%–55.6% interchromosomal rearrangements shared between *S. officinarum* and *S. robustum*. The estimated divergence time of 769 thousand years between *S. spontaneum* and the common ancestor of *S. officinarum* and *S. robustum* added further support to the absence of shared allopolyploid event between *Saccharum* and *Miscanthus*. The common ancestor of these three *Saccharum* species is likely diploid with 10 chromosomes as in sorghum. The reduction in basic chromosome number from 10 to 8 in *S. spontaneum* occurred less than 769 thousand years after the speciation event but before the two rounds of WGD via autopolyploidization as shown by lack of preferential pairing (Ming *et al*., 1998).

*Saccharum officinarum* was speculated to be domesticated from *S. robustum* 10 000 years ago as they share the same basic chromosome number and the same origin and centre of diversity in Papua New Guinea (Roach and Daniels, 1987). However, based on *K*s analysis of large gene sets, we estimated the divergence time of *S. officinarum* and *S. robustum* at 385 thousand years (Table 5 and Figure 6), well before the origin of agriculture in Papua New Guinea about 10 000 years ago. Our results indicate that *S. officinarum* evolved from a common ancestor with *S. robustum* about 385 thousand years and the speciation event is due to natural selection, not artificial selection. There are more interchromosomal rearrangements in *S. officinarum* than in *S. robustum*, which might be caused by the artificial selection in *S. officinarum* after the split of *S. robustum* and *S. officinarum* from their common ancestor.

The divergence time of *S. officinarum* and *S. robustum* is shorter than that of *S. officinarum* and *S. spontaneum*, a finding that is consistent with previous studies that utilized genetic molecular markers (Jsteven *et al*., 2007) and molecular cytogenetics (D'Hont *et al*., 2002). We estimated the divergence time of *S. officinarum* and *S. spontaneum* at 769 thousand years ago, much more recent than that of previous estimates, 1.5–2 mya (Jannoo *et al*., 2007) and 1.9–2.1 mya (Zhang *et al*., 2016). Previous *K*s estimates were based on about a dozen gene pairs on homologous BACs bearing *ADH* and *Bru1* genes that evolve rapidly, while our estimates were based on more than 2000 gene pairs genome wide, eliminating bias from a small gene set in 100‐ to 200‐kb region.

The basic chromosome number of *S. officinarum* and *S. spontaneum* was *x *=* *10 and *x *=* *8 (Bremer, 1961; D'Hont *et al*., 2011; Panje and Babu, 1960), respectively. The reduction in basic chromosome number from 10 to 8 could result from either chromosome fusion or chromosome fission followed by integration of chromosome segments into different chromosomes after the speciation event separating *S. spontaneum* from the common ancestor of *S. officinarum* and *S. robustum*, because *S. spontaneum* linkage groups aligned to all 10 sorghum chromosomes (Ming *et al*., 1998). The two rounds of WGD via autopolyploidization occurred after these chromosome reduction events, likely earlier than the two rounds of WGD events in *S. officinarum* and *S. robustum*, because higher SNP rate was detected among orthologous haplotypes in *S. spontaeum* than those in *S. officinarum* and *S. robustum* (Zhang *et al*., 2013). The genic high‐density genetic maps provided large‐scale collinearity analysis and allowed detection of more interchromosomal rearrangements between *Saccharum* and sorghum chromosomes. In contrast, only one interchromosomal rearrangement was detected from RFLP linkage maps due to the lower density and fewer aligned markers (Ming *et al*., 1998). In *Saccharum* hybrid Q165, four chromosomal rearrangements between Sb5/6, Sb 8/2, Sb 9/8/2and Sb8/7/5 were identified (Aitken *et al*., 2014), and of these four translocations, Sb5/6 and Sb 8/2 were observed in genetic maps of hybrid R570 (Dufour *et al*., 1997). However, only one of (Sb 8/2 in LG26) these four translocations in *Saccharum* hybrids was found in the *S. officinarum* despite the consensus LG covered more than 90% of corresponding sorghum chromosome region. *Saccharum* hybrids were observed to contain 5%–10% of genome from the recombination between *S. officicinarum* and *S. spontaneum* (Cordeiro *et al*., 2006). Compared to the genetic map of *S. officinarum*, the other three chromosomal rearrangements in *Saccharum* hybrids could be the results of genome recombination after the cross between these *S. officinarum and S. spontaneum*.

SNPs have been reported for sugarcane (Aitken *et al*., 2014; Bundock *et al*., 2009; Cordeiro *et al*., 2006; Garcia *et al*., 2013; Grivet *et al*., 2001, 2003), but none of them has used genic SNPs for genetic mapping. Different from diploid, the high level of homologous redundancy in *Saccharum* species for SNPs genetic mapping offers a ‘fresh challenge’ (McKay and Leach, 2011). The main obstacles of this challenge are to identify the applicable SNPs for genetic mapping in high polyploidy. The two parents of the mapping population are autooctoploid, and allele dosage for a given locus in *Saccharum* can be 0 (aaaaaaaa), single dose (Aaaaaaaa), double dosage (AAaaaaaa), triple dose (AAAaaaaa) and quadruple doge (AAAAaaaa). In a reciprocal pseudo‐test cross (F_1_ population of two heterozygous parents), the single‐dose DNA markers segregate as 1 : 1 (presence: absence) when one of the parents is heterozygous (Wu *et al*., 1992); the double dose (AAaaaaaa), triple dose (AAAaaaaa) and quadruple dose (AAAAaaaa) are segregated as 3 : 14, 1 : 14 and 1 : 70 (presence: absence), respectively, when one of the parents is heterozygous for the F1 population (Silva *et al*., 1993). Similar to other DNA markers, by considering a heterozygous SNP allele as ‘presence’, SNPs mapping can use this binary marker system (presence: absence). In *S. officinarum* LA Purple, the total SNPs were estimated to be 17.7 million per genome according to its monoploid genome size (985 Mb) and SNPs density (1.8%) in the *bru1* genomic region (Zhang *et al*., 2016). About 70% of SNPs in the *SuSy* fragment haplotype are single dose in *Saccharum*, which is consistent with the estimation from the segregation ratios of different plex loci (Silva *et al*., 1993). Therefore, single dosage SNPs are sufficient for genetic mapping in a polyploid F_1_ population.

To detect allelic SNPs, the depth of coverage increases with ploidy level. Diploids require 7.73 coverage for the sequencing of both alleles greater than 99% of the time. Tetraploids have been suggested to require read depths of at least 153 for the identification of each allele (Griffin *et al*., 2011). Furthermore, a sequencing depth of 48 was recommended to accurately genotype autotetraploids with 95% of certainty (Uitdewilligen *et al*., 2013). In *Saccharum* species, for an applicable single‐dose SNP in autopolyploid with 2n=mX, the allelic ratio is 1a:(m‐1) A for one parent (P1) and 0a: mA for another one (P2) theoretically. It is difficult to identify the dosage of SNPs for individuals in the highly polyploid plants, for instance, in octopolyploid, to discover a single‐dose SNPs with a possibility (certainty) of 95% (not cover all alleles), a sequencing depth of 22 is required [*P* = 1−(1 − *p*)^*n*^, *n* = depth, *p* = 1/8]. In an F1 population, the allelic ratios are deduced to be 1a : 15A for the single‐dose SNPs according to the genetic transmission balance. A minimum depth of 72 can discover 99% of single‐dose SNP [*P* = 1−(1 − *p*)^*n*^, *n* = depth, *p* = 1/16]. In this study, merging the aligned sequences from the 59 F1 progenies provides high depth with average of 330 for SNP calling. Because the potential homologous chromosome expression dominance and the bias of sequence depth among the progenies could cause SNP allelic ratio deviation, we analysed the distribution of allelic ratios of SNPs (Figures S3a and 3b). Based on previous studies, about 70% of SNPs and other molecular markers are single dose in *Saccharum*. Therefore, we tested a range of allelic ratios around 1a : 15A, and allelic ratios from 1a : 6A to 1a : 30A were consequently used to achieve 70% of SNPs.

The limitation of a genic linkage map based on RNA‐Seq data is that the expression of genes or alleles affects marker distribution on each chromosome. Although deep RNA sequencing often covers 80% genes in the genome, the genes expressed in each tissue should be randomly distributed among chromosomes, except co‐expression of some genes on the same pathway (Williams and Bowles, 2004).

For sequencing and assembly of autopolyploid genomes, ultra‐high‐density linkage maps are an essential genomic resources to separate the homologous chromosomes and identify large‐scale interchromosomal rearrangements. Such maps should be generated from re‐sequencing the genomes of the mapping populations to cover both the genic and nongenic regions of the chromosomes. Combining long sequence reads, Hi‐C physical mapping and ultra‐high‐density linkage mapping, chromosome level assembly of autopolyploid genomes is achievable.

## Conclusions

*Saccharum officinarum* and *S. robustum* have similar chromosome structure as sorghum despite large amounts of intrachromosomal arrangements. Twenty‐four interchromosomal rearrangements detected in *Saccharum*–sorghum were shared by *S. officinarum* and *S. robustum*, indicating that polyploidization events occurred after their speciation event. The speciation event separating *S. officinarum* and *S. robustum* occurred about 385 thousand years ago. The common ancestor of *S. officinarum* and *S. robustum* diverged from *S. spontaneum* about 769 thousand years ago. Therefore, the polyploidization events were less than 385 000 years in *S. officinarum* and *S. robustum*, and less than 769 000 years in *S. spontaneum*. These estimates are substantially shorter than the 1.5–2 million years reported previously based on a small genomic region harbouring genes under intensive selection.

## Experimental procedures

### Plant materials

Three varieties of *Saccharum* species were used for RNA‐Seq analysis in the study: *S. officinarum* LA Purple (2*n* = 8*x* = 80), *S. robustum* Molokai 5829 (2*n* = 8*x* = 80) and *S. spontaneum* SES 208 (2*n* = 8*x* = 64). The tissues from three different stems, mature leave (the mixing of top visible dewlap leaf +1, +2 and +3) and leaf rolls of three *Saccharum* species were collected for RNA isolation.

An interspecific F1 population from the cross of *S. officinarum* (LA Purple) and *S. robustum* (Molokai 6081) containing 59 individuals, maintained in Hawaii, was used in the study. The mature leaf tissues (the mixing of top visible dewlap leaf +1, +2 and +3) were harvested from the individuals of the population after 10‐month growth.

### RNA‐Seq analysis

Total RNA was extracted using TRIzol^®^ (Invitrogen, Carlsbad, California). The cDNA libraries were prepared using Illumina^®^ TruSeq™ RNA Sample Preparation Kit (RS‐122‐2001(2), Illumina, San Diego, California) according to the manufacturer's protocol. A pair‐end library for LA Purple was made using Illumina^®^ TruSeq™ RNA Sample Preparation Kit (RS‐122‐2001(2), Illumina). The library samples were sequenced on the Illumina HiSeq 2000 with 120 cycles by the KECK centre in UIUC (http://www.biotech.uiuc.edu/). A preprocessing quality control filter was imposed for both quality (>30) and depth of coverage (>7) to remove false positives due to PCR duplicates and low‐quality reads.

### Transcriptome assembly

The filtered RNA‐Seq reads from the five tissues of three varieties of *Saccharum* species were assembled using CLC Genomics Workbench 5.0 (CLC Bio, Aarhus, Denmark) with default settings, and then, potential poly‐A tails were removed with EMBOSS trimest (Rice *et al*., 2000) followed by finalizing with MIRA (Chevreux *et al*., 2004) and CAP3 (Huang and Madan, 1999).

### Divergence time estimates between sugarcane species and sugarcane–sorghum

The assembled transcripts from the three varieties of *Saccharum* species were aligned against the predicted sorghum‐coding sequences (downloaded from Phytozome version 8.0; http://www.phytozome.net/) using LAST aligner (Kielbasa *et al*., 2011). The sugarcane transcripts with sum of matching HSPs exceeding 50% of the length of the best sorghum orthologs were retained for further analyses. The ‘filtered’ sequence set was clustered using CD‐HIT‐EST (Li and Godzik, 2006) with default parameters. For each pairwise species comparison among the four species, orthologous pairs of genes that are reciprocal best hits to one another are extracted from the LAST output (Kielbasa *et al*., 2011).

The calculation of *K*s values uses the same pipeline as described in Wang *et al*. (2010). Briefly, the protein sequences of orthologous gene pairs were aligned by CLUSTALW (Larkin *et al*., 2007), converted back to DNA (codon) alignments with PAL2NAL (Suyama *et al*., 2006). Substitutions per synonymous site or *K*s values for these gene pairs were calculated using Nei‐Gojobori method implemented in PAML (Yang, 2007). The species divergence time was estimated by this formula: *T_div* = median *K*s/(2 × 6.5e^−9^) (Gaut *et al*., 1996). A Python script used to process the calculations is available at http://github.com/tanghaibao/bio-pipeline/tree/master/synonymous_calculation/.

### SNP calling

The libraries of 59 F_1_ progenies and LA Purple were aligned to the sorghum gene models using Novoalign with the default alignment settings (http://www.novocraft.com/main/index.php). However, to account for the estimated 95% sequence identity between sorghum and *Saccharum* (Wang *et al*., 2010), the alignment score threshold settings were made less stringent (i.e. ‐t 95 option that allows ~5 bp mismatch).

A custom Perl script was used to group the number of markers into bins of markers of the 59 individuals with less than 10% missing data. Each bin would be comprised of markers with similarity scores less than the cut‐off for all markers. A representative marker of each bin was manually chosen to have the least amount of missing data.

Theoretically, the ratio of minor to major allele frequency for single dosage SNP is deducted to be 1 : 15. Each parent has eight allelic positions at a given reference nucleotide with a combined 16 nucleotide positions. There was one nucleotide mutated in one parent, hence 1 : 15 ratio in the F1 population at that particular nucleotide position. Segregation of single‐dose SNPs in F_1_ progenies is 1 : 1. Considering the variation of sequence depth at specific positions in individual libraries and potential subgenome dominance, a range of ratios from 1 : 6 to 1 : 30 was used to screen the SNPs from the alignment results of merging data of progenies. After the alignments of RNA‐Seq of progeny individuals through Novoalign, the alignment results of progenies ‘mpileup’ were merged using SAMtools. SNPs were detected and scored by Perl script (https://github.com/lileiting/Pileup2singledose) from the merged data with a ratio from 1 : 6 to 1 : 30 and individual base quality scores of >28 as thresholds. Meanwhile, using the ‘pileup’ command of SAMtools, the alignment result of LA Purple was then screened by Perl script with a ratio from 1 : 6 to 1 : 30. Progenies were aligned to each of the parents, and the presence/absence haplotypes were called of the parent SNPs (Figure S9).

### Genetic linkage mapping

Markers were sorted into linkage groups based on LOD score using JoinMap 4.1.

Linkage groups were made at a cut‐off LOD [logarithm (base 10) of odds] score of 6. Unmapped bins/markers due to this criterion were manually moved to their respective linkage in the map with an SCL value of 4 or greater. The linkage groups served as bin lists, which were expanded into marker lists. Each marker list was run in JoinMap separately. To minimize error, the largest linkage group at LOD score 4 was chosen to be the ideal marker list. The marker list was analysed using JoinMap's regression mapping with Kosambi's function algorithm. For each added locus, position is found by comparing the goodness‐of‐fit of the calculated map for each tested position. Should the goodness‐of‐fit decrease sharply, the locus is removed. A second round of testing may add loci previously removed, increasing number of markers without compromising fit of map. A third round would force fit all markers in the linkage group, providing a map that may not be accurate. The second map has the highest confidence with highest number of markers, therefore Map 2 is the one reported, though it has fewer markers than Map 3.

### Synteny analysis

LA Purple and Molokai 6081 markers were paired with the sorghum genes found as their homologs. Linkage groups with significant synteny (more than five homologous markers) between cultivars and *Sorghum* were visualized using Strudel and Circos programs.

### Availability of supporting data

Trimmed and quality‐filtered Illumina reads for the RNA‐Seq data have been deposited in the NCBI BioProject database (http://www.ncbi.nlm.nih.gov/bioproject) under accession number: PRJNA388550.

## Conflict of interest

The authors declare no conflict of interest.

## Supporting information


**Figure S1** The distribution of aligned reads within SPS gene among different libraries.
**Figure S2** Overview of single‐dose SNPs calling based on merged alignments RNA‐Seq libraries.
**Figure S3** (a and b) Distribution of single dose SNPs for parents based on qualified plant numbers and segregation ratios. (a) Distribution of single dose SNPs for female parent based on qualified plant numbers and segregation ratios. (b) Distribution of single dose SNPs for male parent based on qualified plant numbers and segregation ratios.
**Figure S4** The distribution of bin marker of LG in the sorghum genome.
**Figure S5** The collinearities between *S. officinarum* and *S. robustum*.
**Figure S6** Interchromosomal rearrangements between *Saccharum* and sorghum chromosomes.
**Figure S7** Intrachromosomal rearrangements between *Saccharum* and sorghum chromosomes.
**Figure S8** Intrachromosomal rearrangements of homologous group among *Saccharum* chromosomes.
**Figure S9** Technology roadmap for this study.

**Table S1** Genetic maps for *S. officinarum*.

**Table S2** Genetic maps for *S. robustum*.

**Table S3** The distribution of bin markers in linkage groups of *Saccharum* species.

**Table S4** The conserved interchromosomal rearrangements of sorghum–*S. officinarum* and sorghum–*S. robustum* shared in *S. officinarum* and *S. robustum*.
