# Supplementary material for: Recent polyploidization events in three Saccharum founding species
Source: Plant Biotechnol J. 2018 Jul 24;17(1):264–74. doi: 10.1111/pbi.12962 (PMC6330536; doi:10.1111/pbi.12962)

**The list of figures in Supplemental Figure 1-9:**

**Supplemental Figure 1.** The distribution of aligned reads within SPS gene among different libraries

**Supplemental Figure 2.** Overview of single-dose SNPs calling based on merged alignments RNA-seq libraries

**Supplemental Figure 3 A&B.** Distribution of single dose SNPs for parents based on qualified plant numbers and segregation ratios

**Supplemental Figure 3A.** Distribution of single dose SNPs for female parent based on qualified plant numbers and segregation ratios

**Supplemental Figure 3B.** Distribution of single dose SNPs for male parent based on qualified plant numbers and segregation ratios

**Supplemental Figure 4** The distribution of bin marker of LG in the sorghum genome

**Supplemental Figure 5.** The collinearities between *S. officinarum* and *S. robustum*

The collinearities between *S. officinarum* and *S. robustum* were shown by the alignment of *Saccahrum* homologous linkage groups to Sorghum chromosome*.* Dark blue color is the background of the chromosomes/LGs. The grey lines are the position of genetic markers/genes. The dark blue color in the center of the sorghum chromosome indicated that low density of genes existed in the regions of chromosome. The bars on the sugarcane LG represent markers from table S[1](https://www.ncbi.nlm.nih.gov/pmc/articles/PMC4222257/#S1). These are aligned using the BLASTN algorithm (P < e−20) and the position indicated by lines to the other chromosomes.The most colinear linkage groups for each of the 10 HG were selected to present .

**Supplemental Figure 6.** Interchromosomal rearrangements between *Saccharum* and sorghum chromosomes

The typical interchromosomal rearrangements between *Saccharum* and sorghum chromosomes were selected to presented.

**Supplemental Figure 7** Intrachromosomal rearrangements between *Saccharum* and sorghum chromosomes

**Supplemental Figure 8**  Intrachromosomal rearrangements of homologous group among *Saccharum* chromosomes.

**Supplemental Figure 9**  Technology roadmap for this study

Supplemental Figure 1 The distribution of aligned reads within SPS gene among different libraries


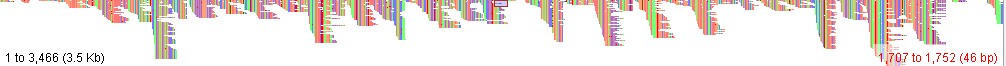

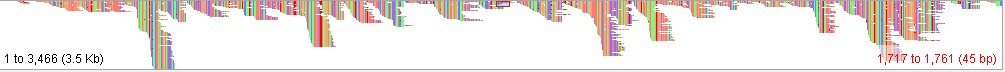

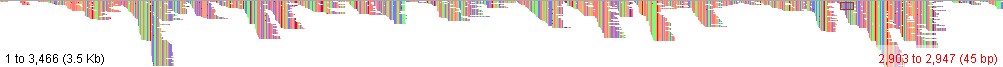

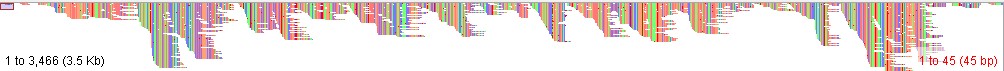

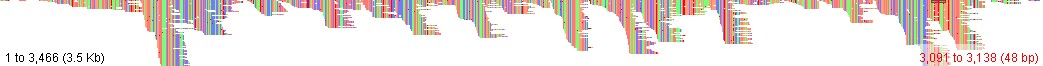

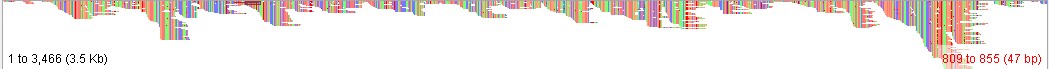

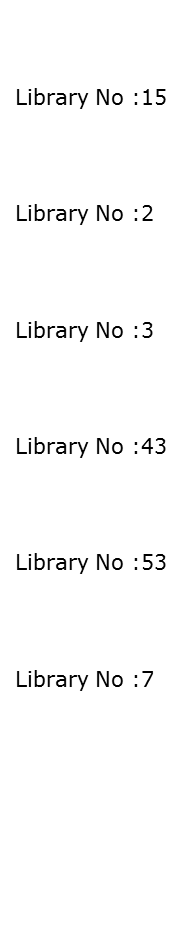


Supplemental Figure 2. Overview of single-dose SNPs calling based on merged aligned RNA-seq libraries


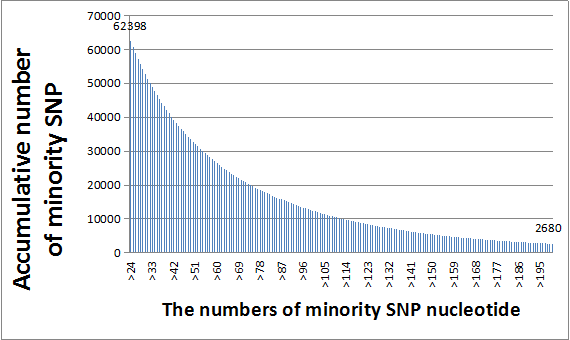


The candidate single-dose SNP numbers for 60 merge RNA-seq libraries based on allelic ratio with range from 1:6 to 1:30.

Supplemental Figure 3A. Distribution of single dose SNPs for female parent based on qualified plant numbers and segregation ratios


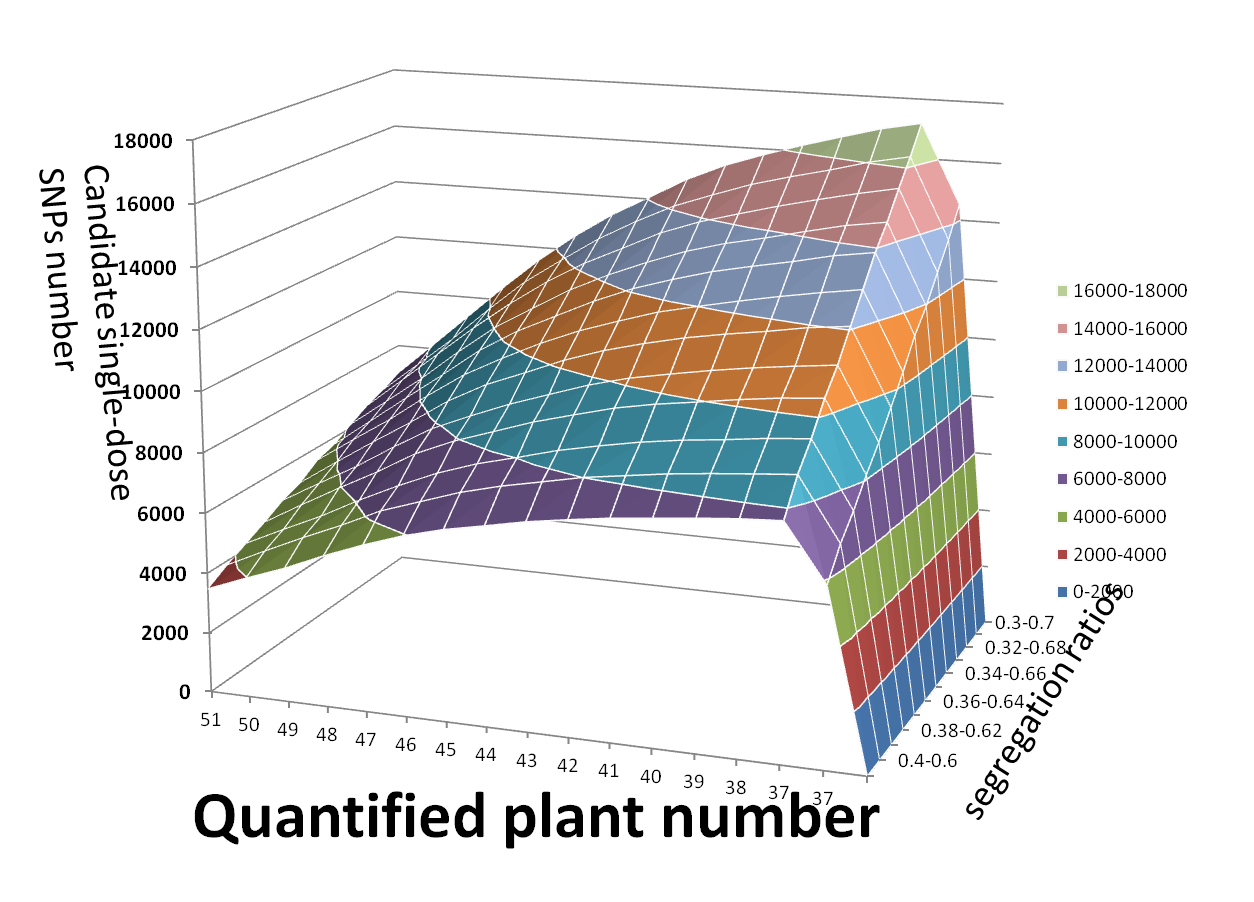


Supplemental Figure 3B. Distribution of single dose SNPs for male parent based on qualified plant numbers and segregation ratios


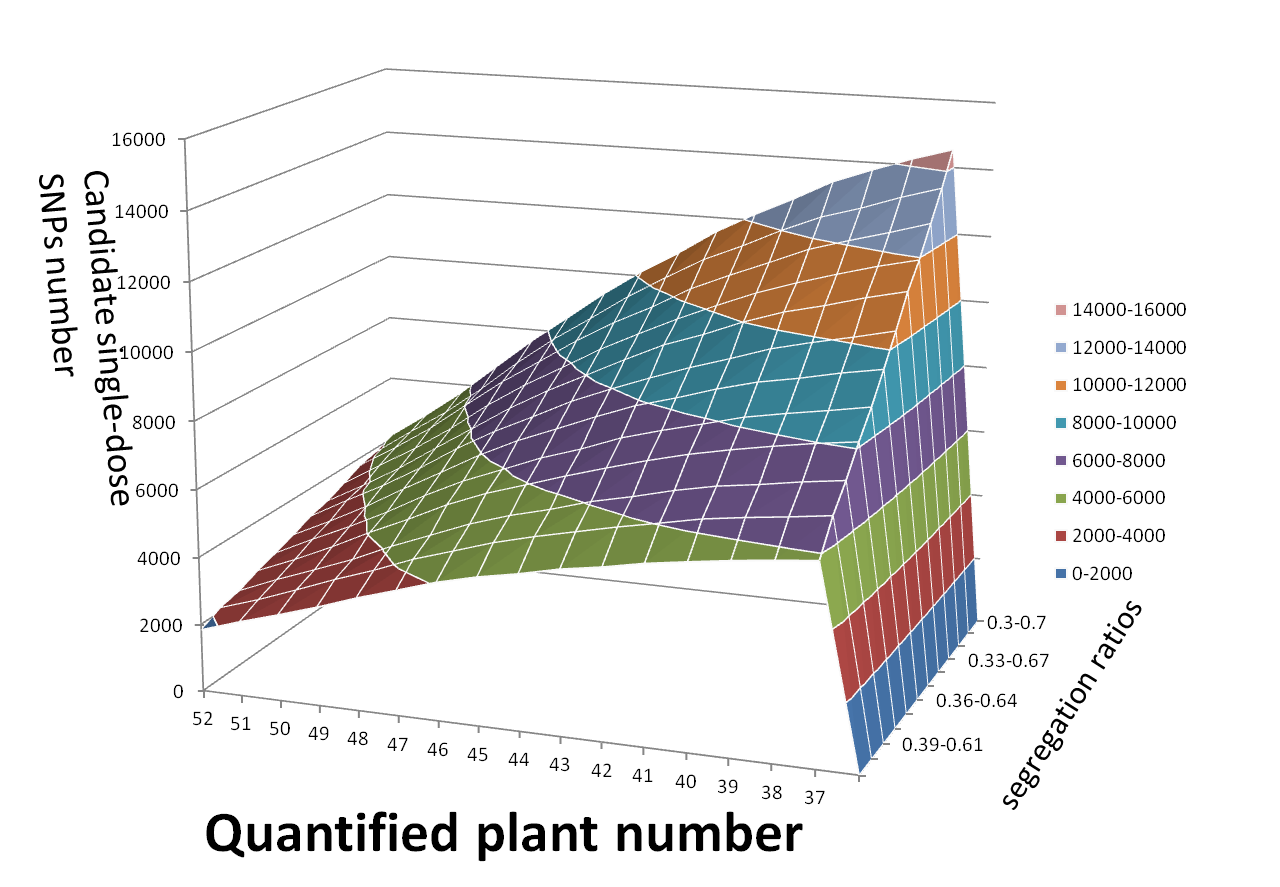


Supplemental Figure 4 The distribution of Bin Maker of LG in the Sorghum genome

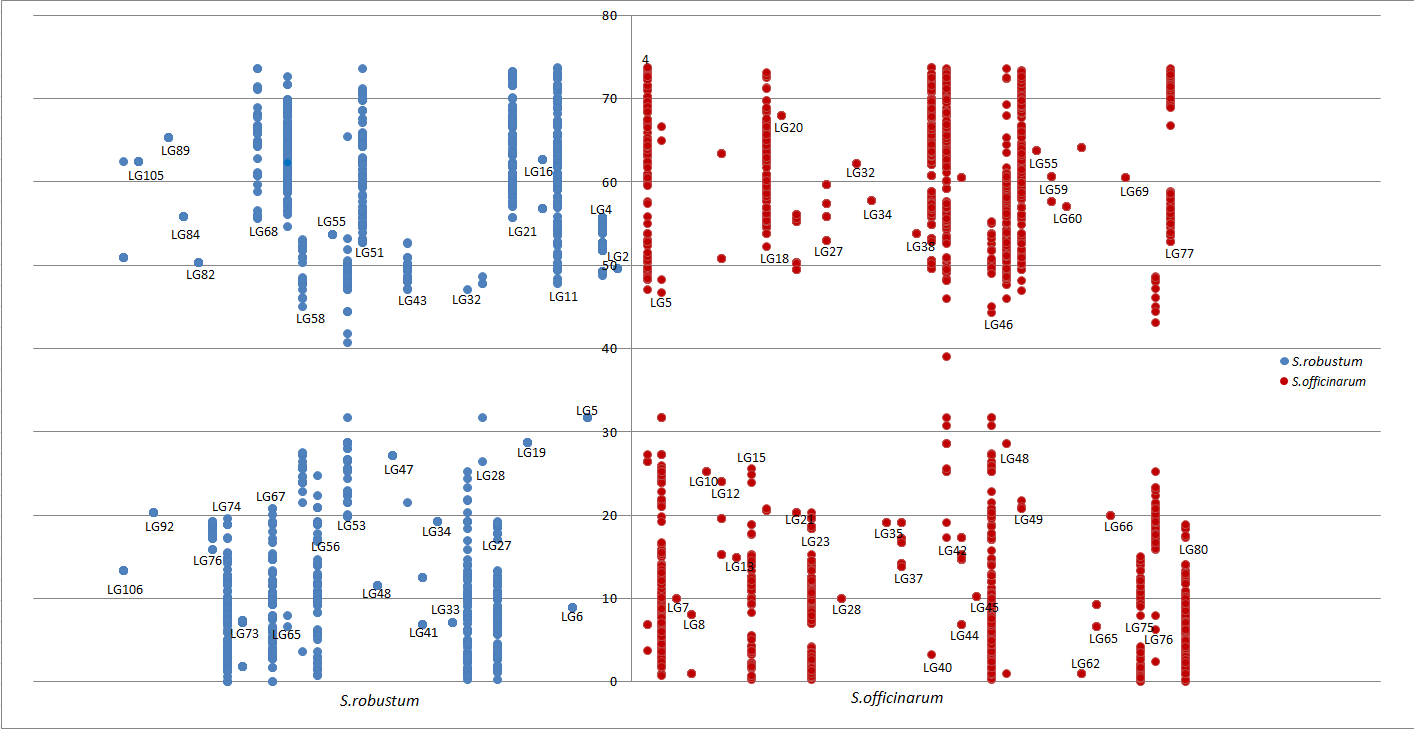

 Chr.1


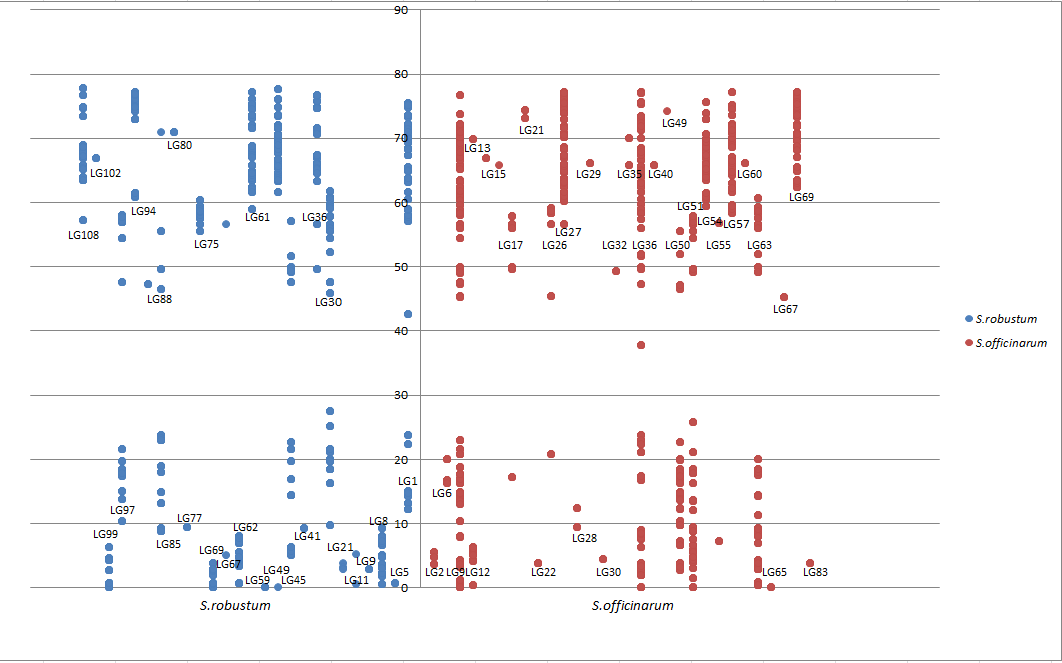


Chr.2


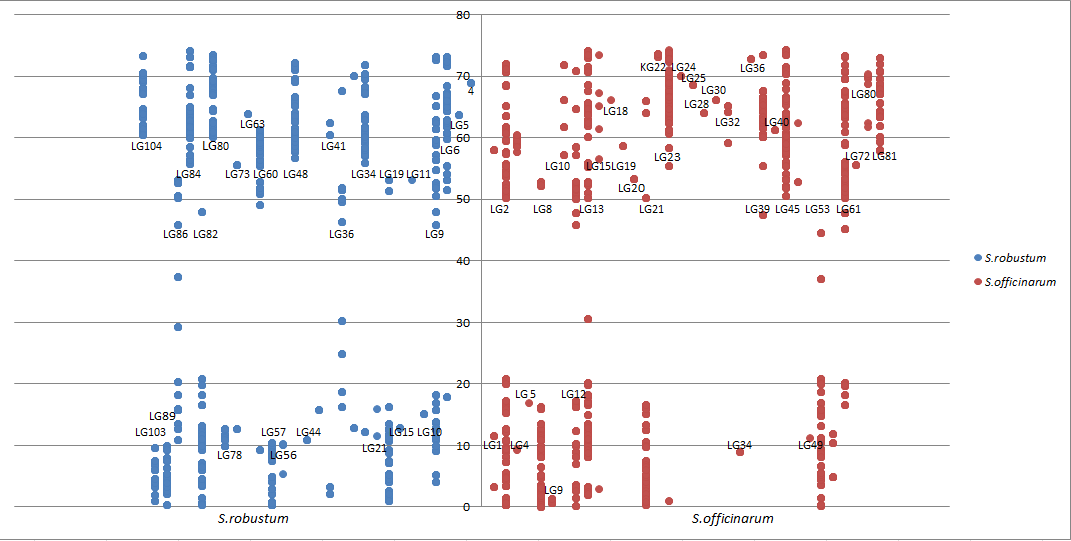


Chr.3


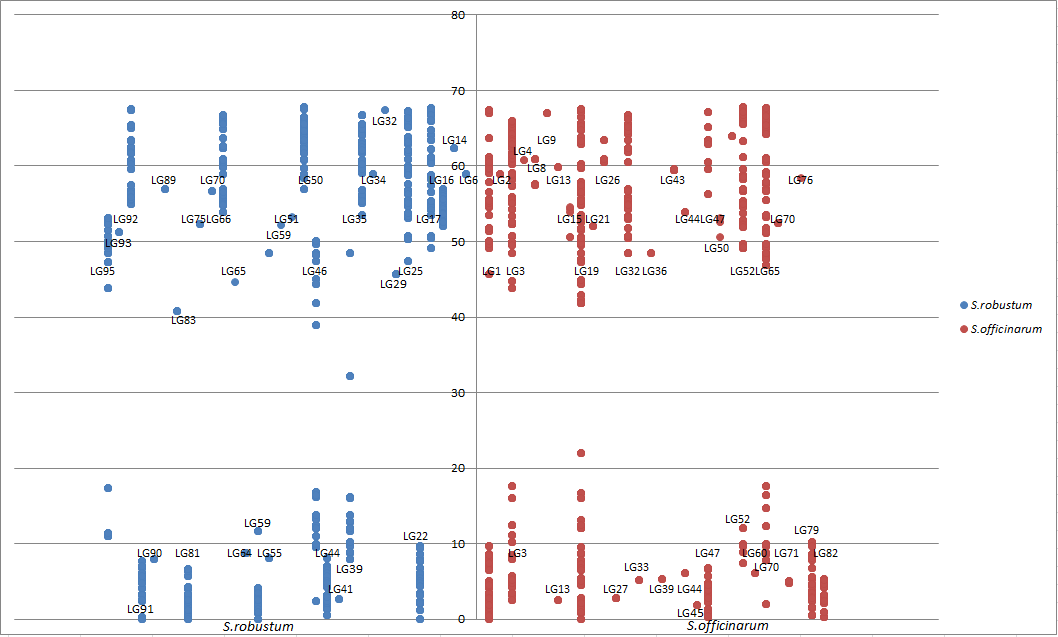


Chr.4


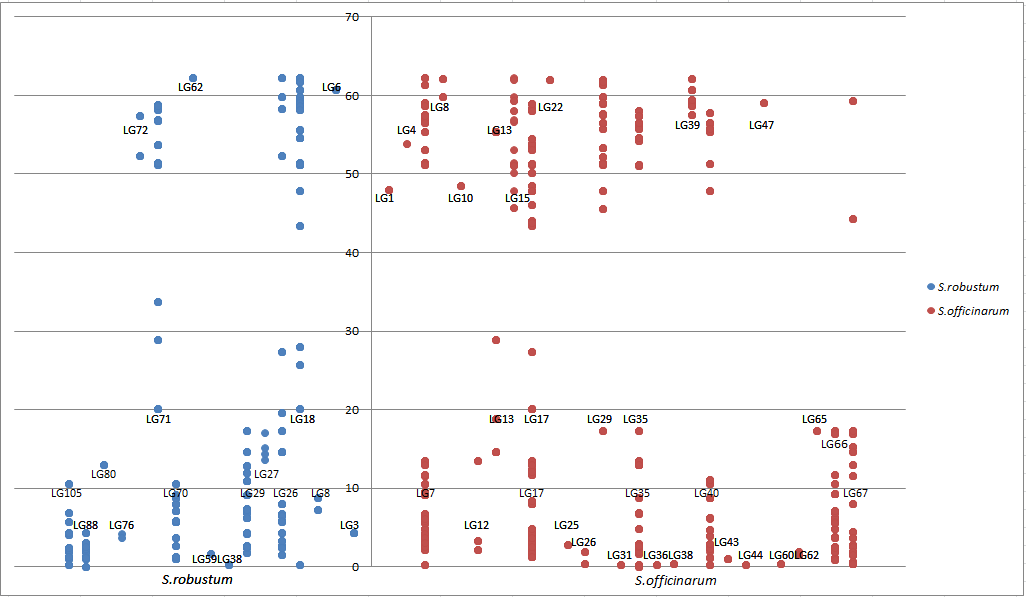


Chr.5


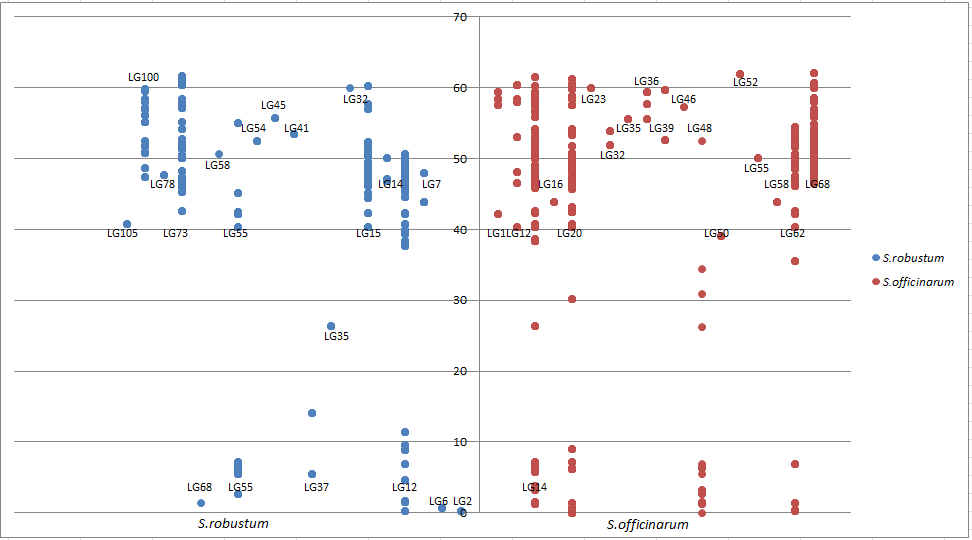


Chr.6


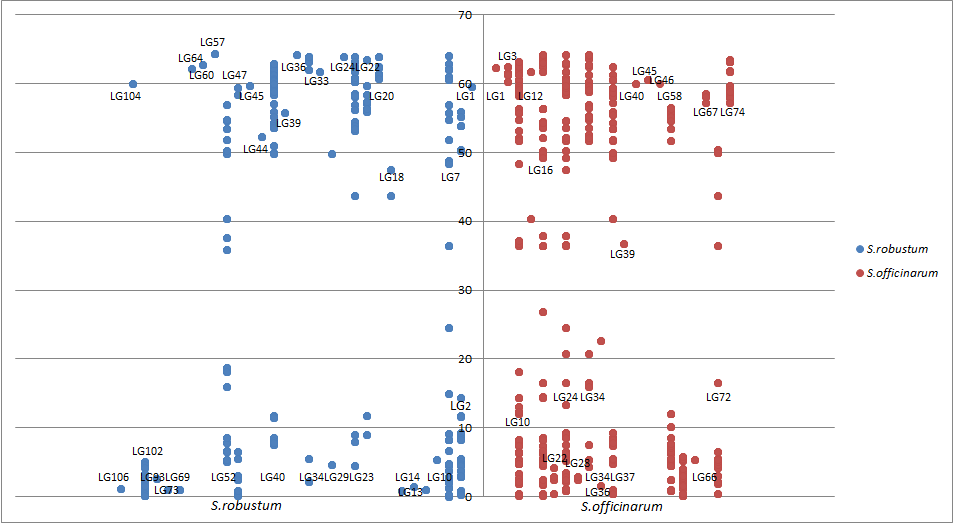


Chr.7


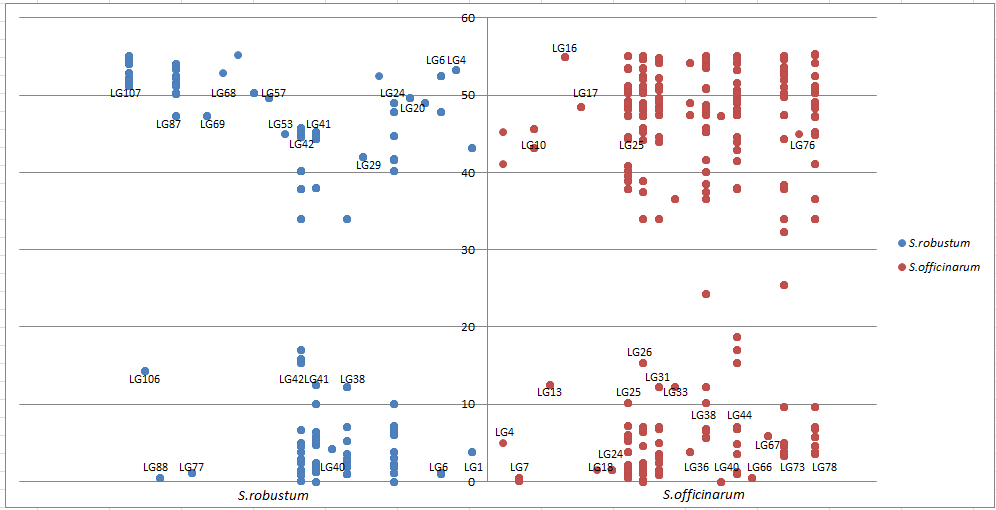


Chr.8


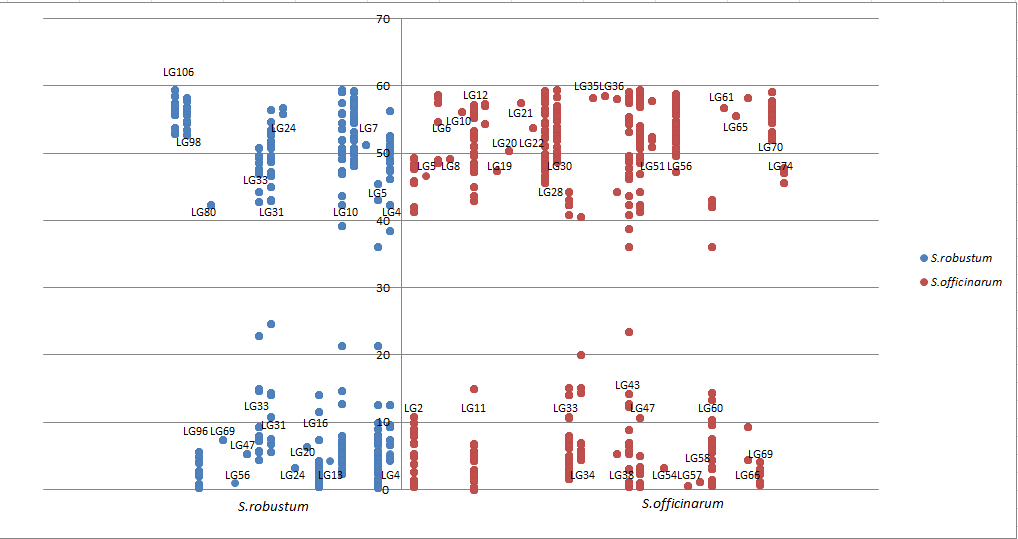


Chr.9


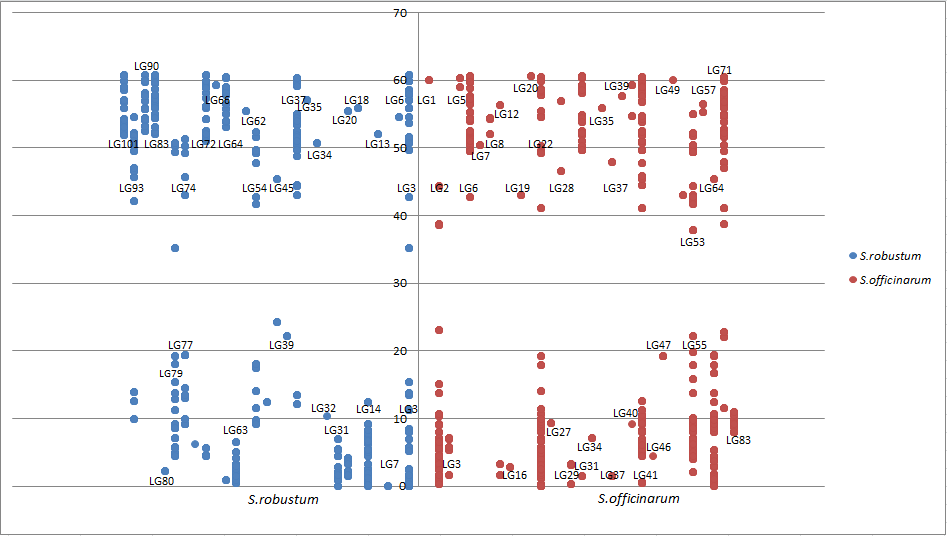


**Supplemental Figure 5:** The colinearities between *S. officinarum* and *S. robustum*


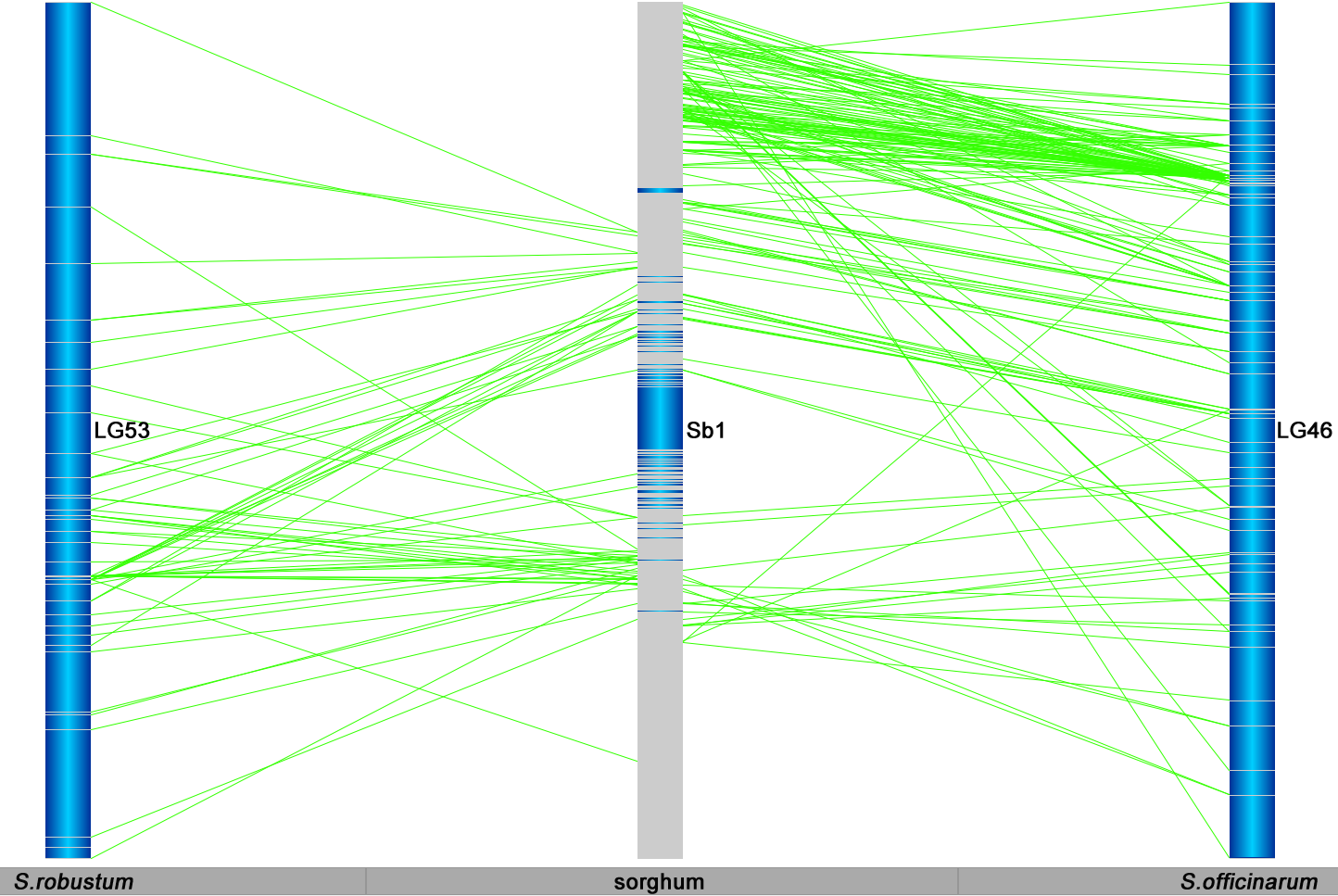


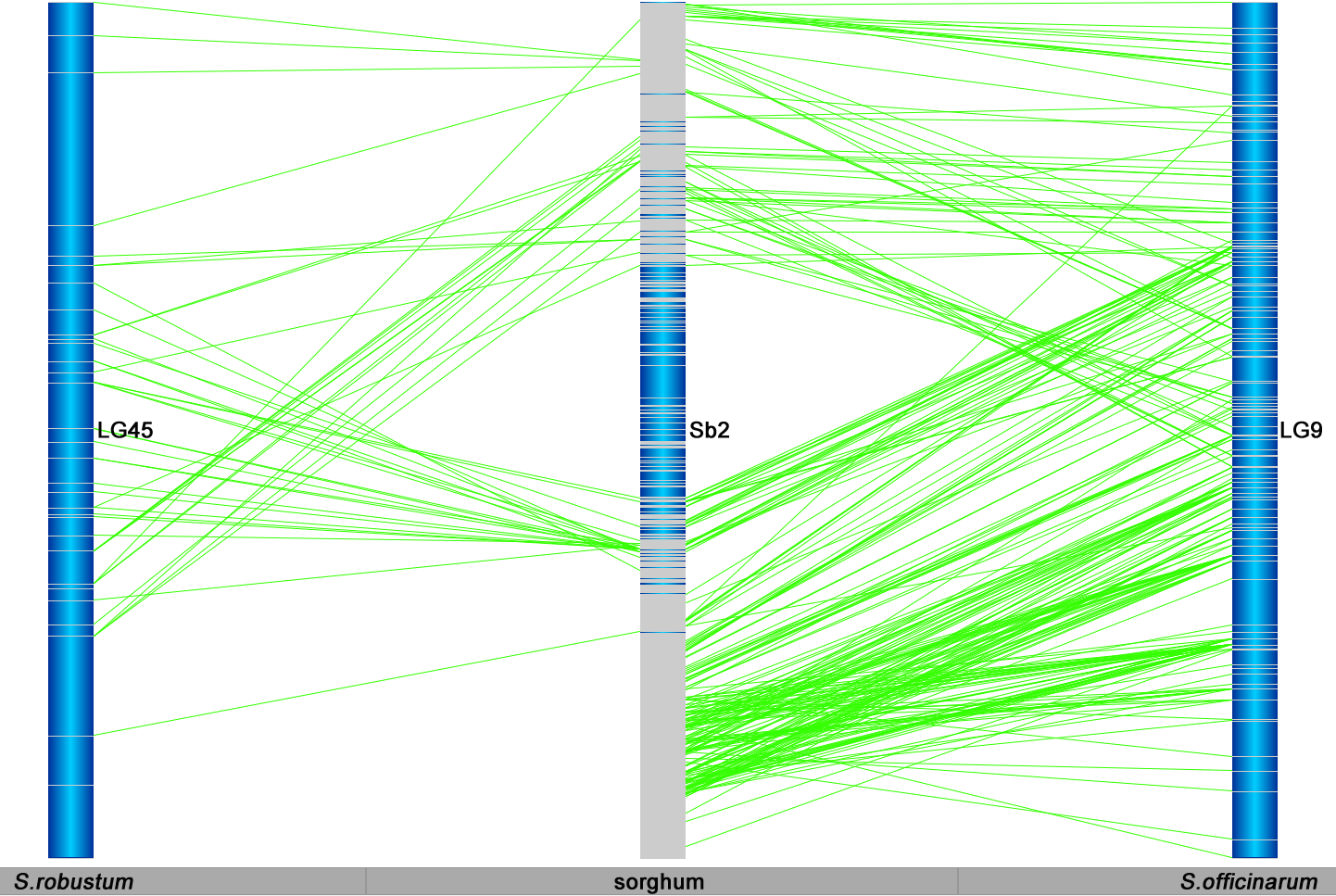


**
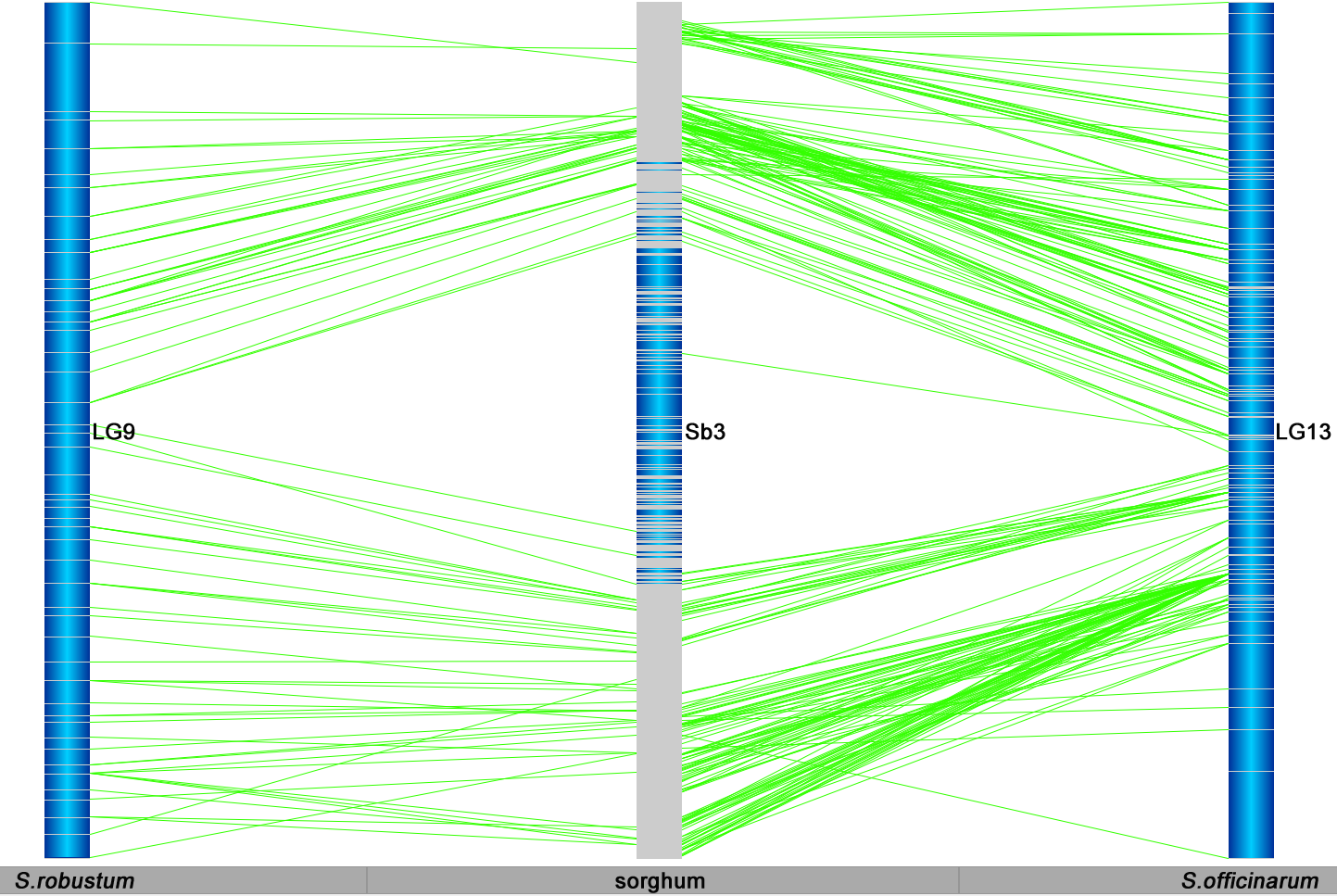
**

**
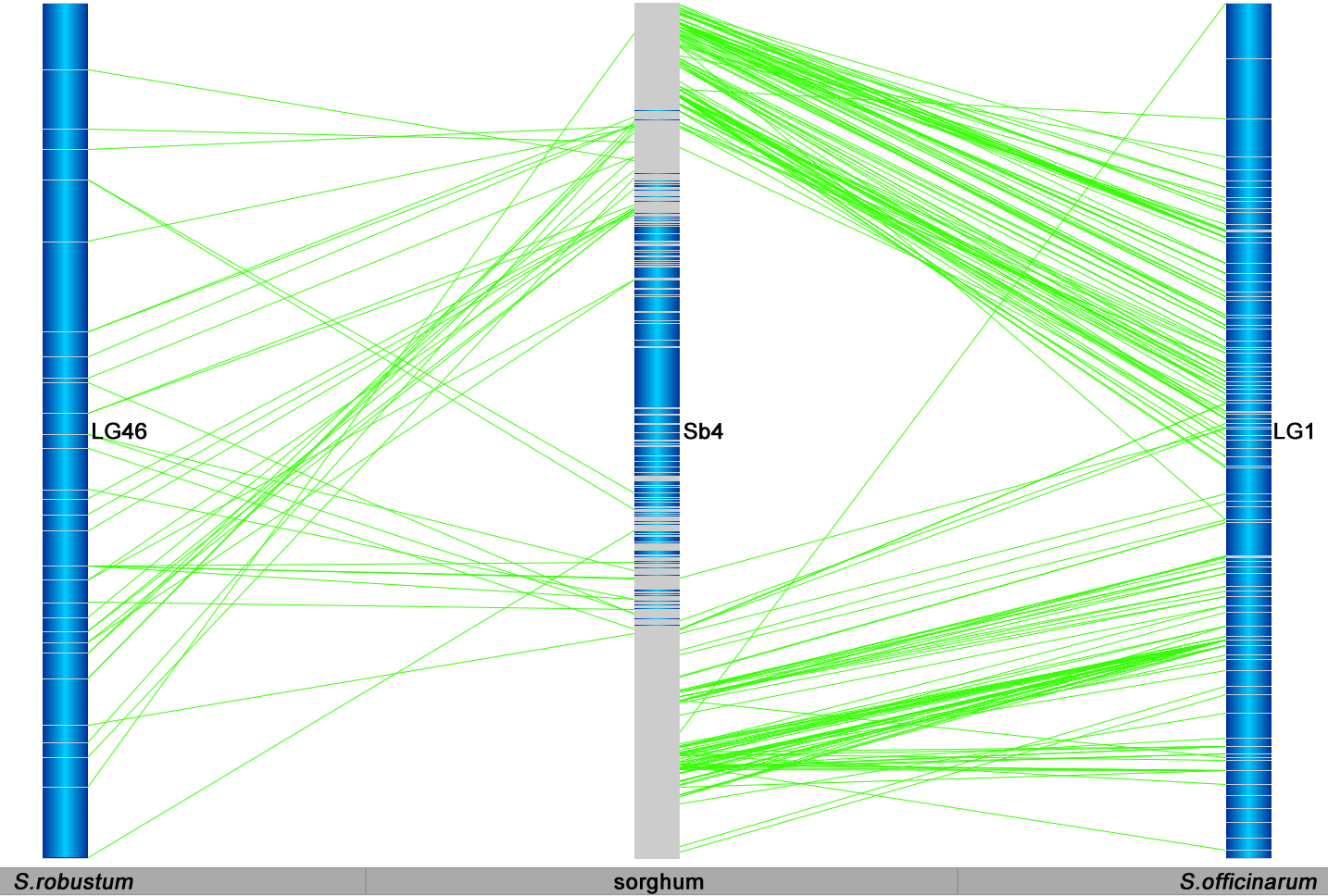
**

**
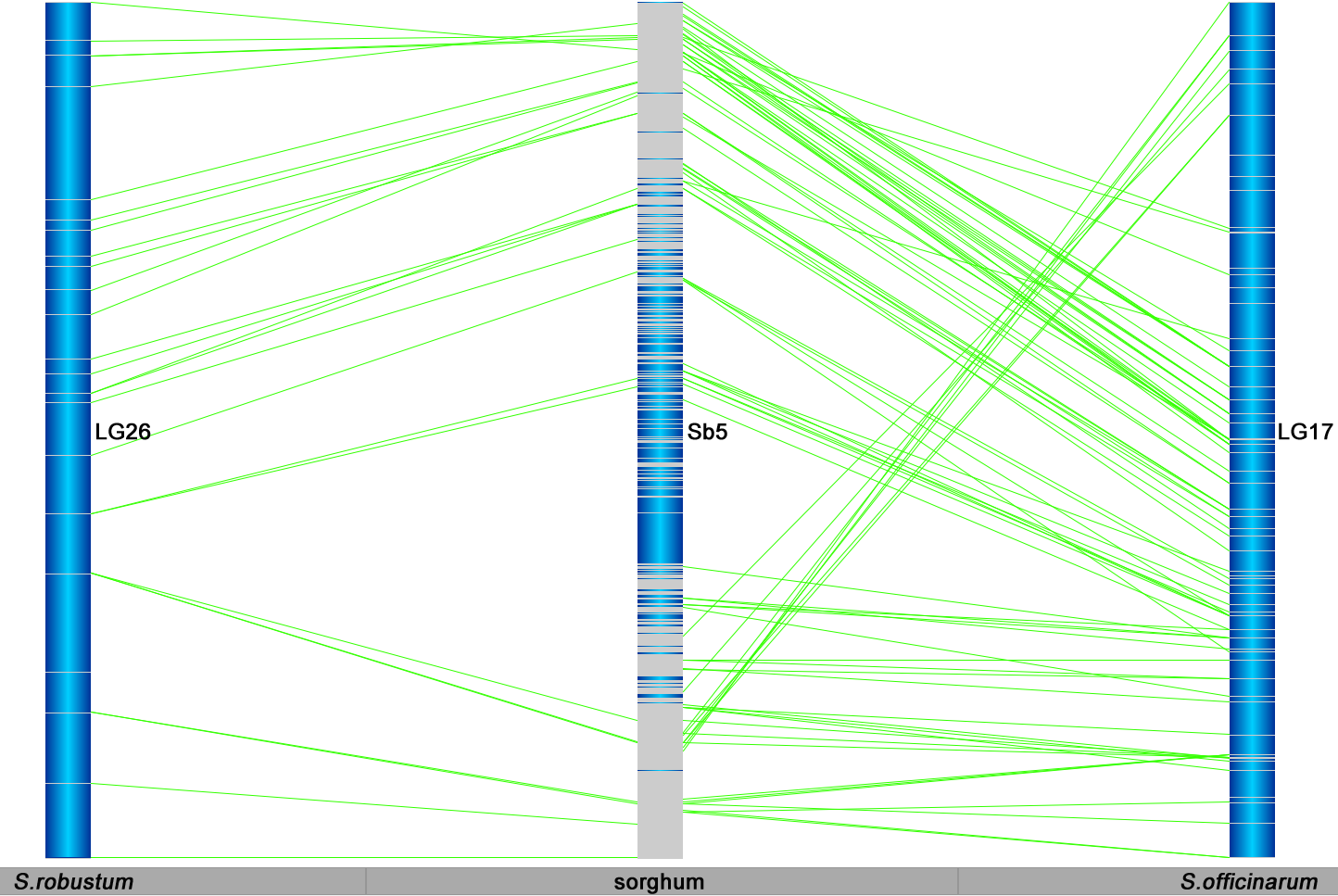
**

**
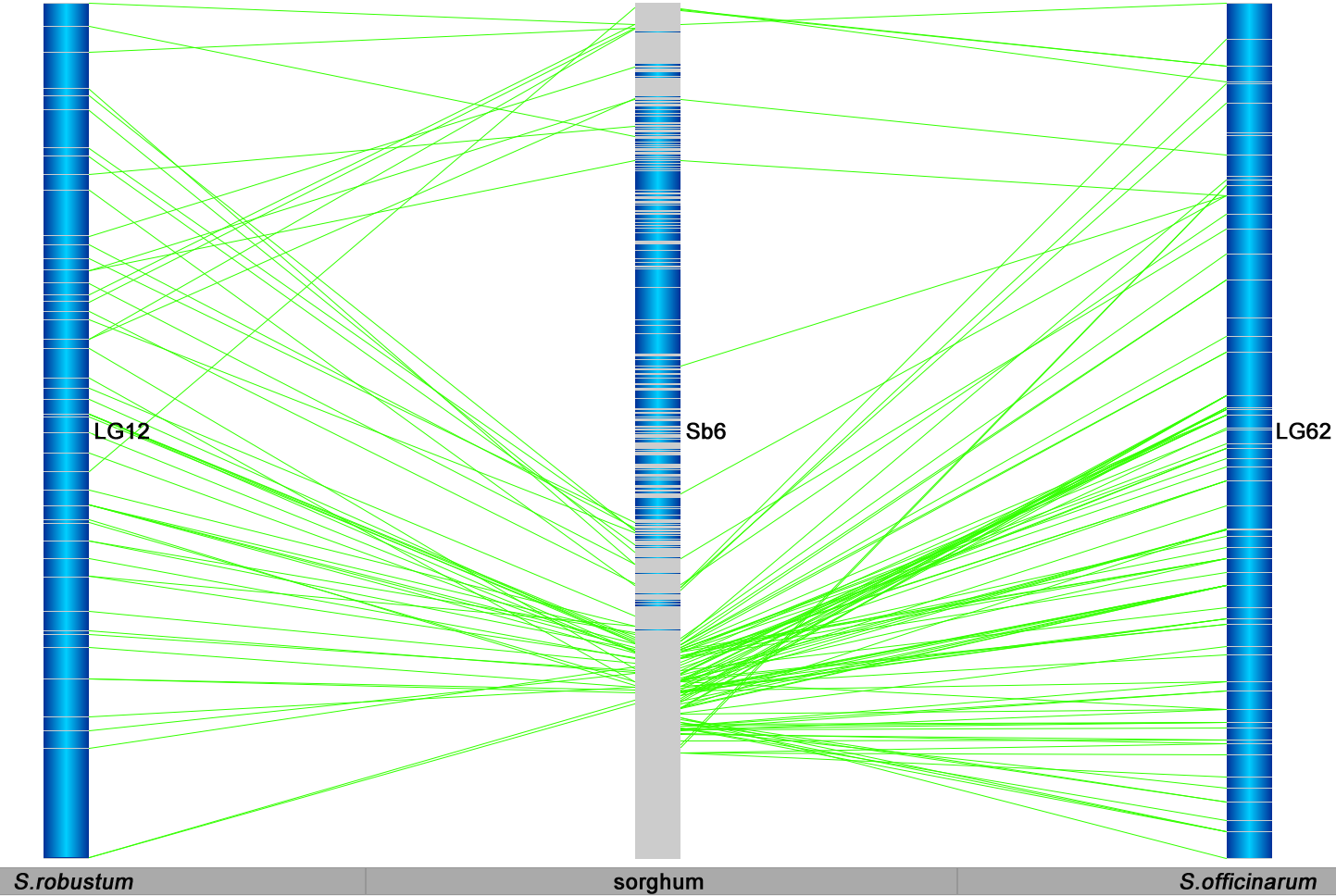
**

**
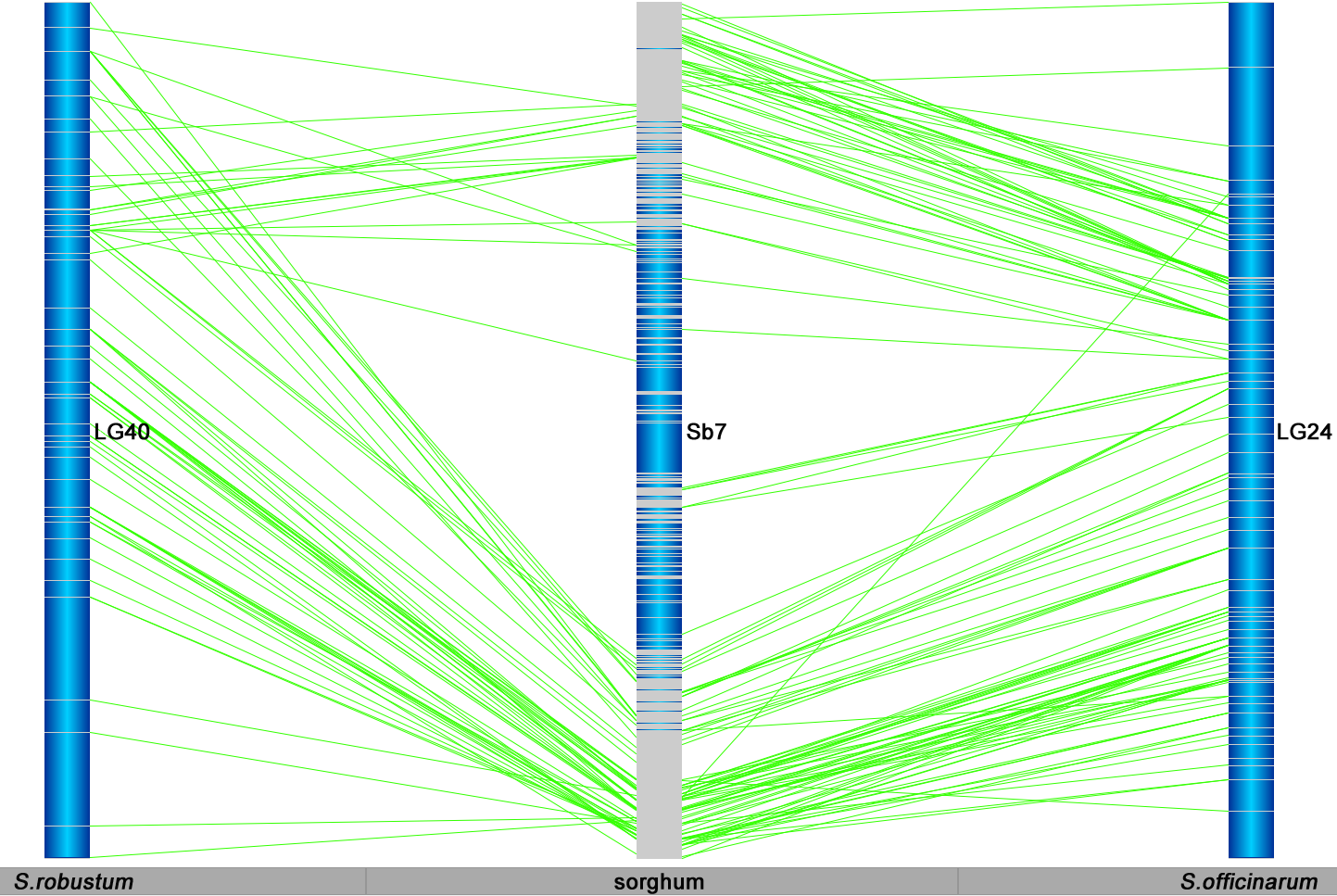
**

**
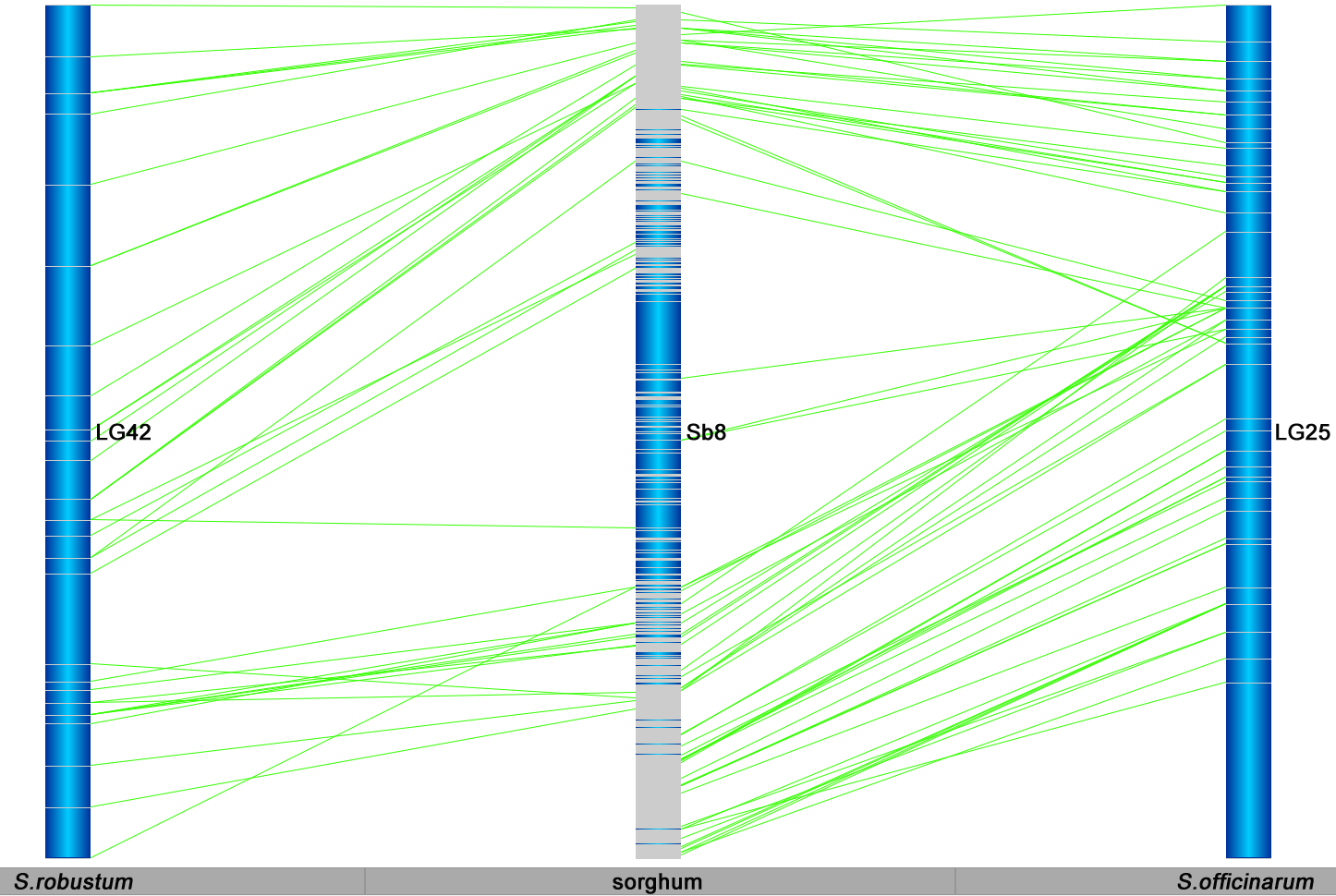
**

**
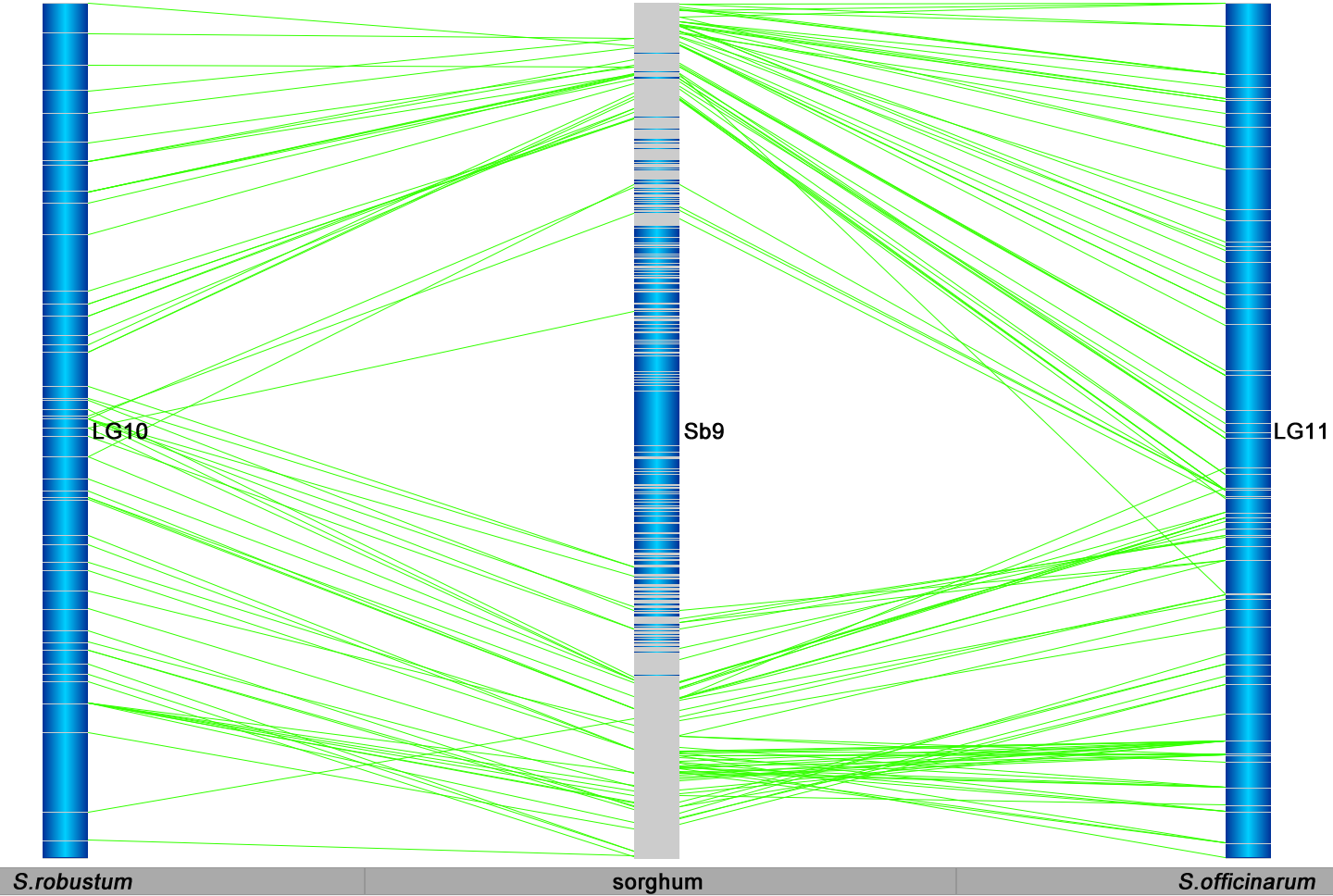
**

**
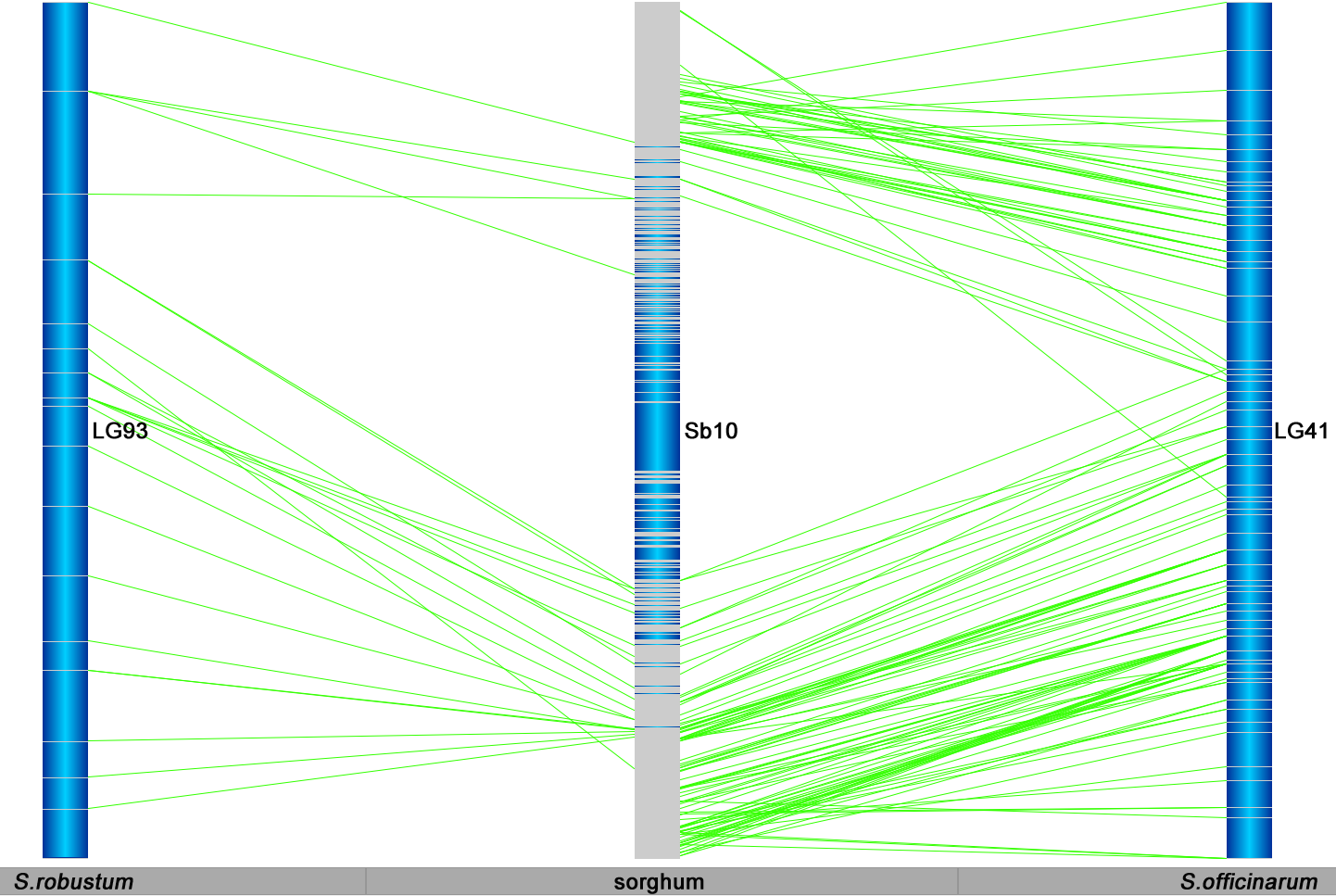
**

Supplemental Figure 6. Intrachromosomal rearrangements between Saccharum and sorghum chromosome
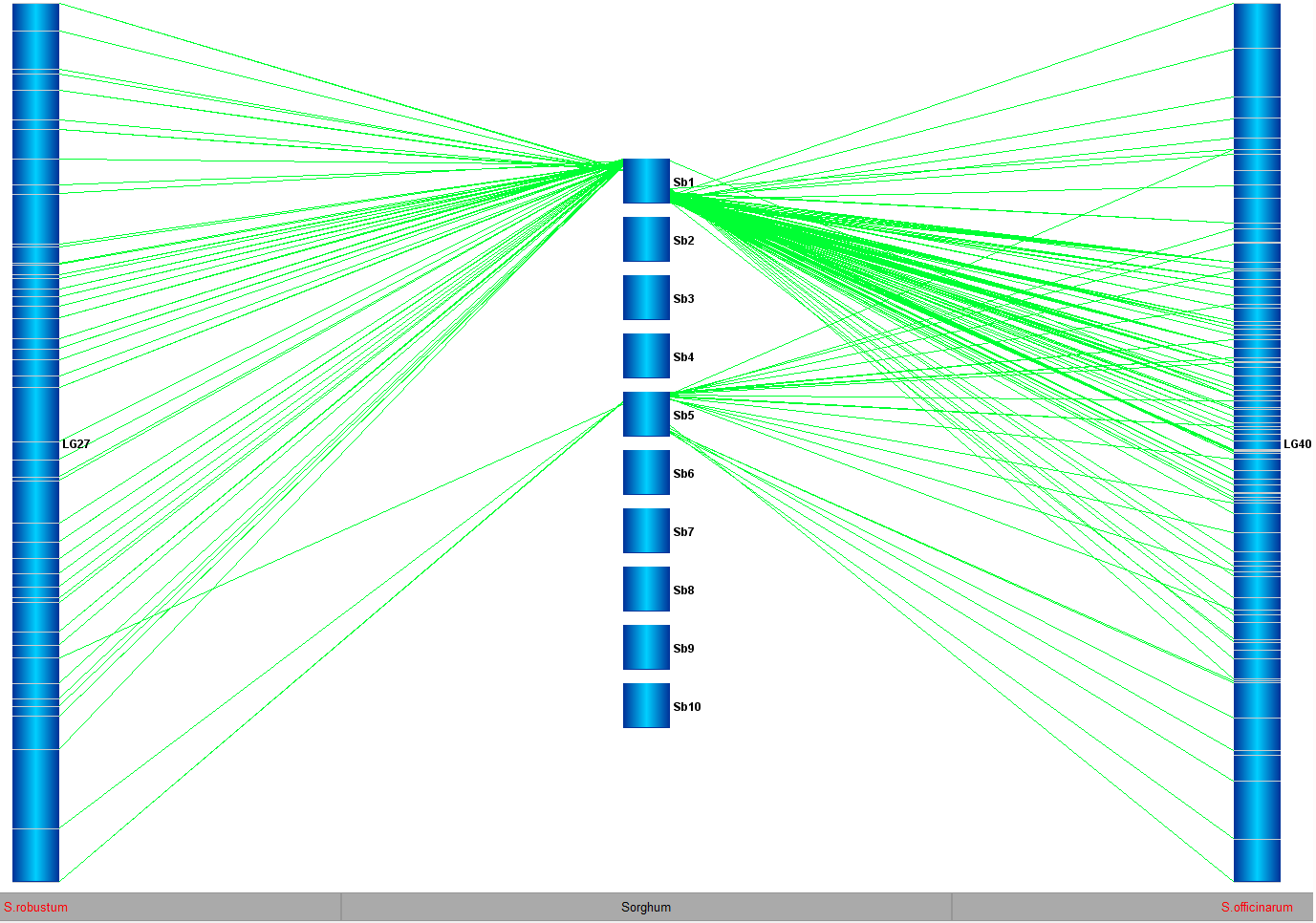

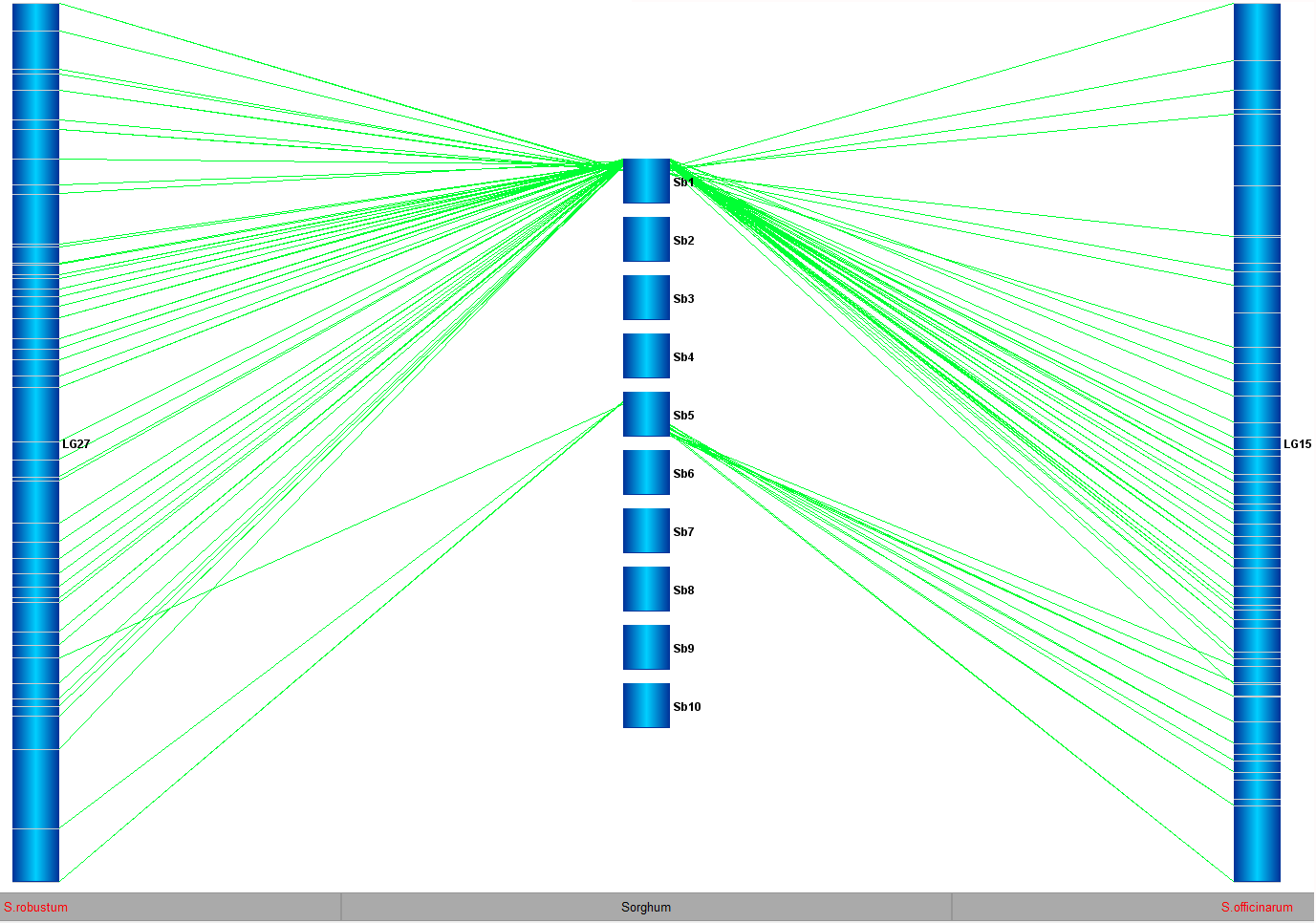


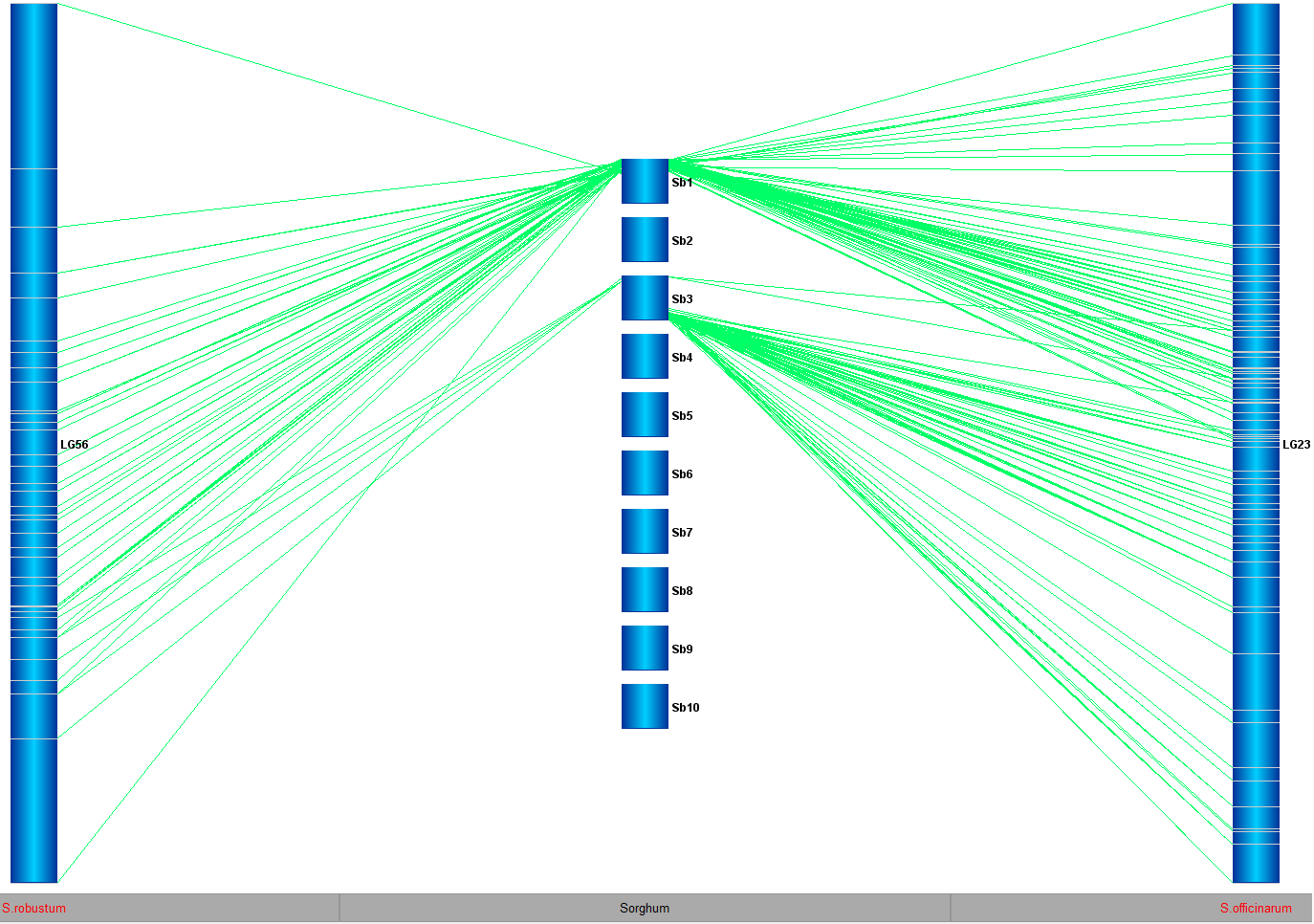


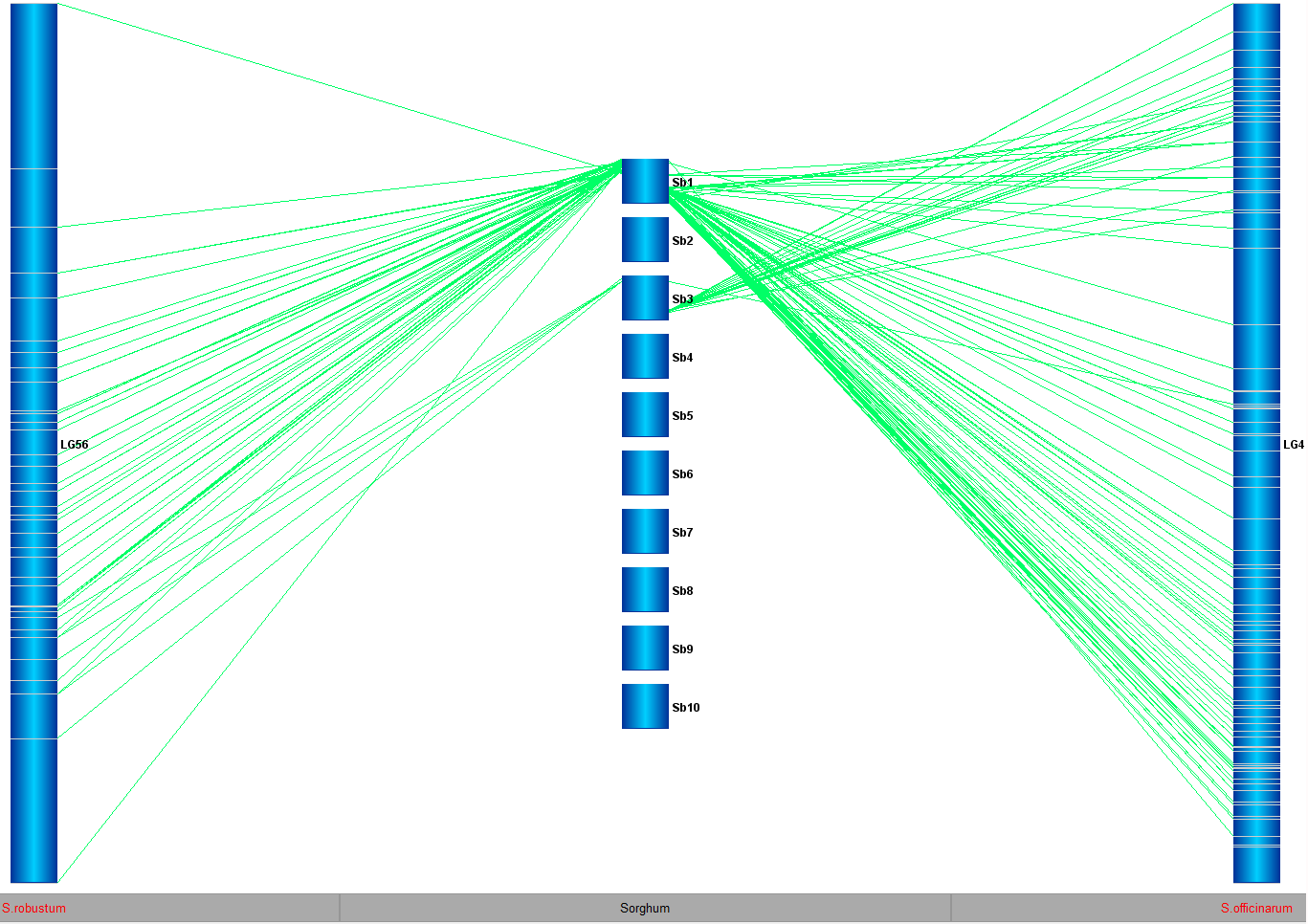

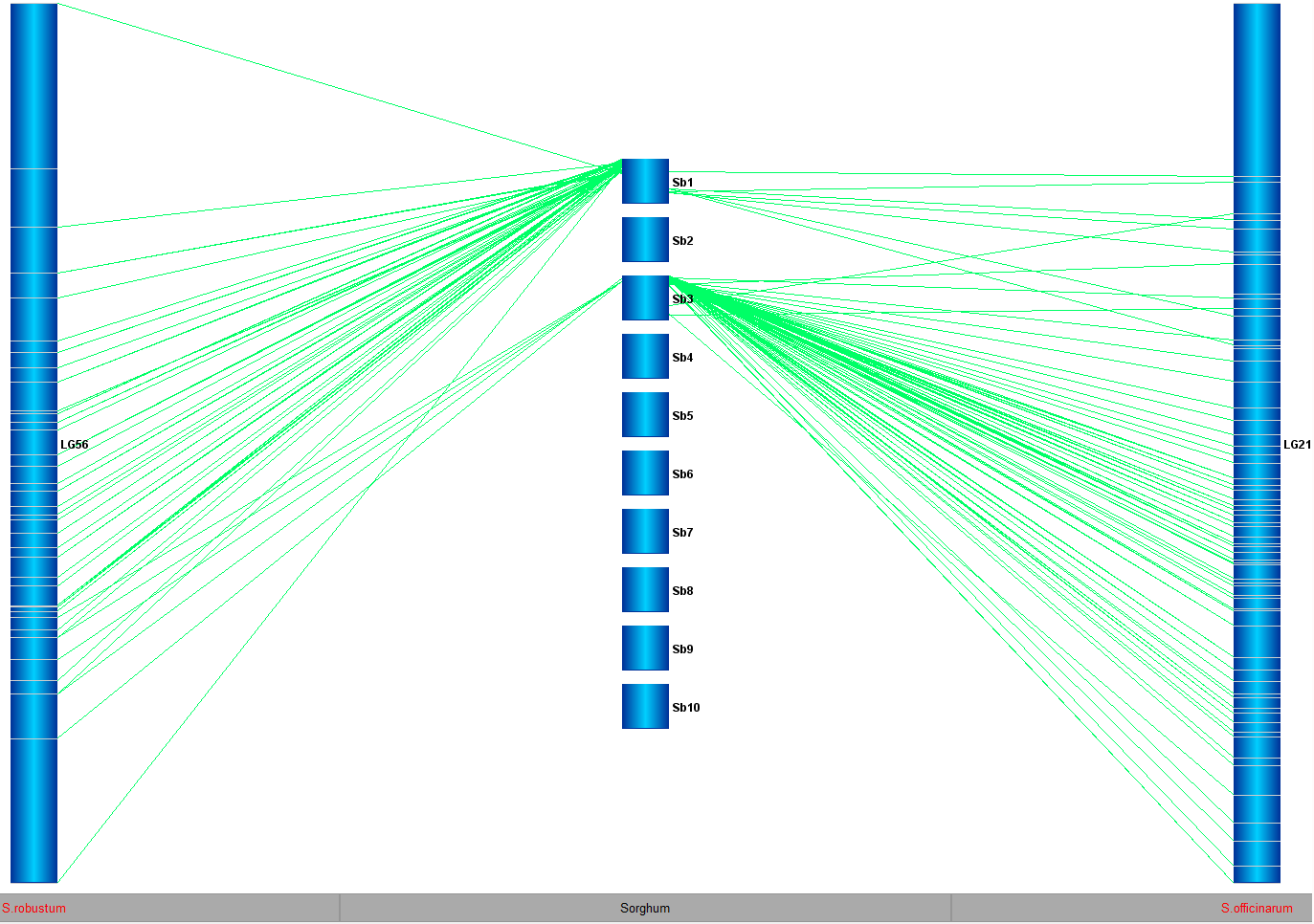


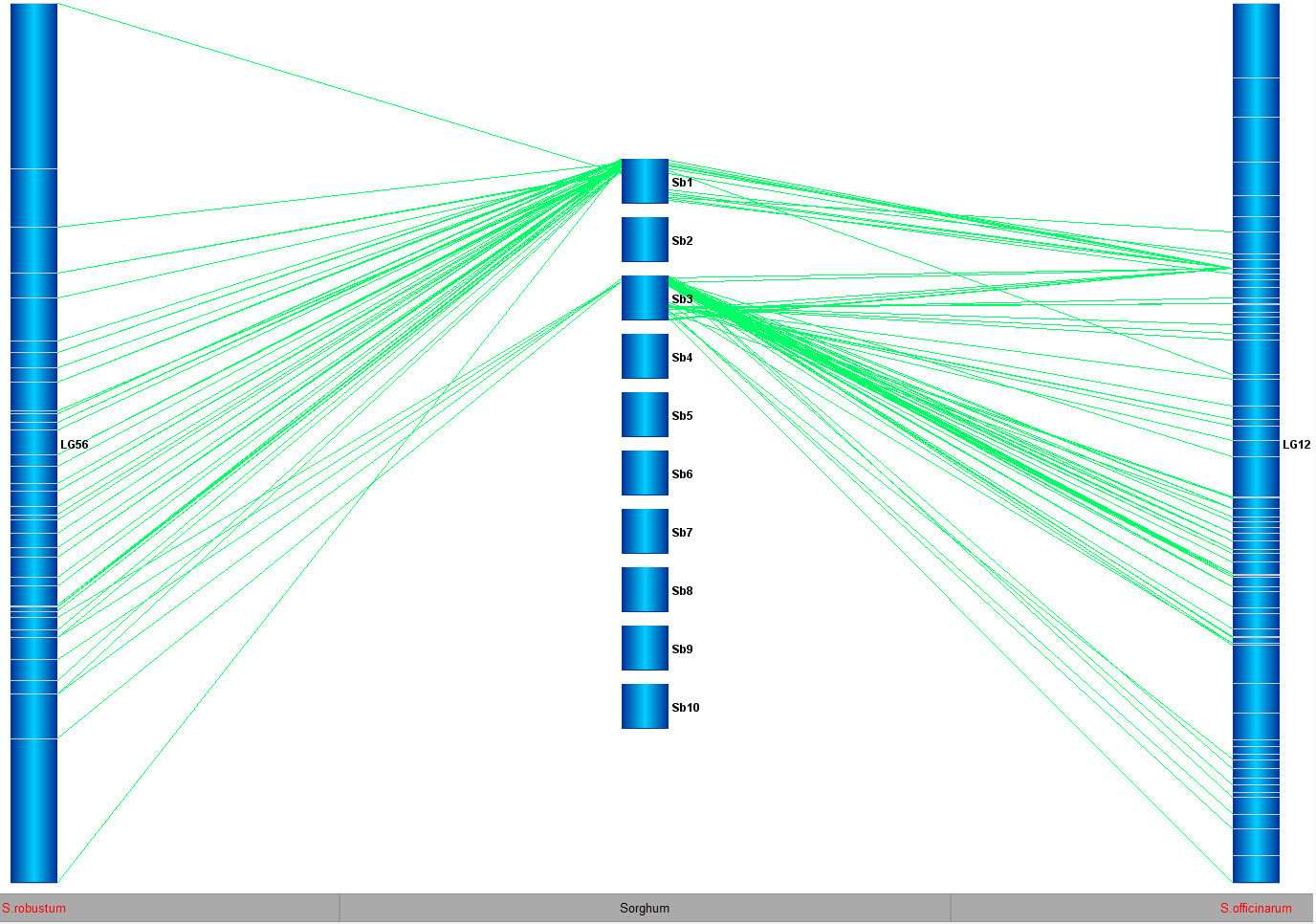


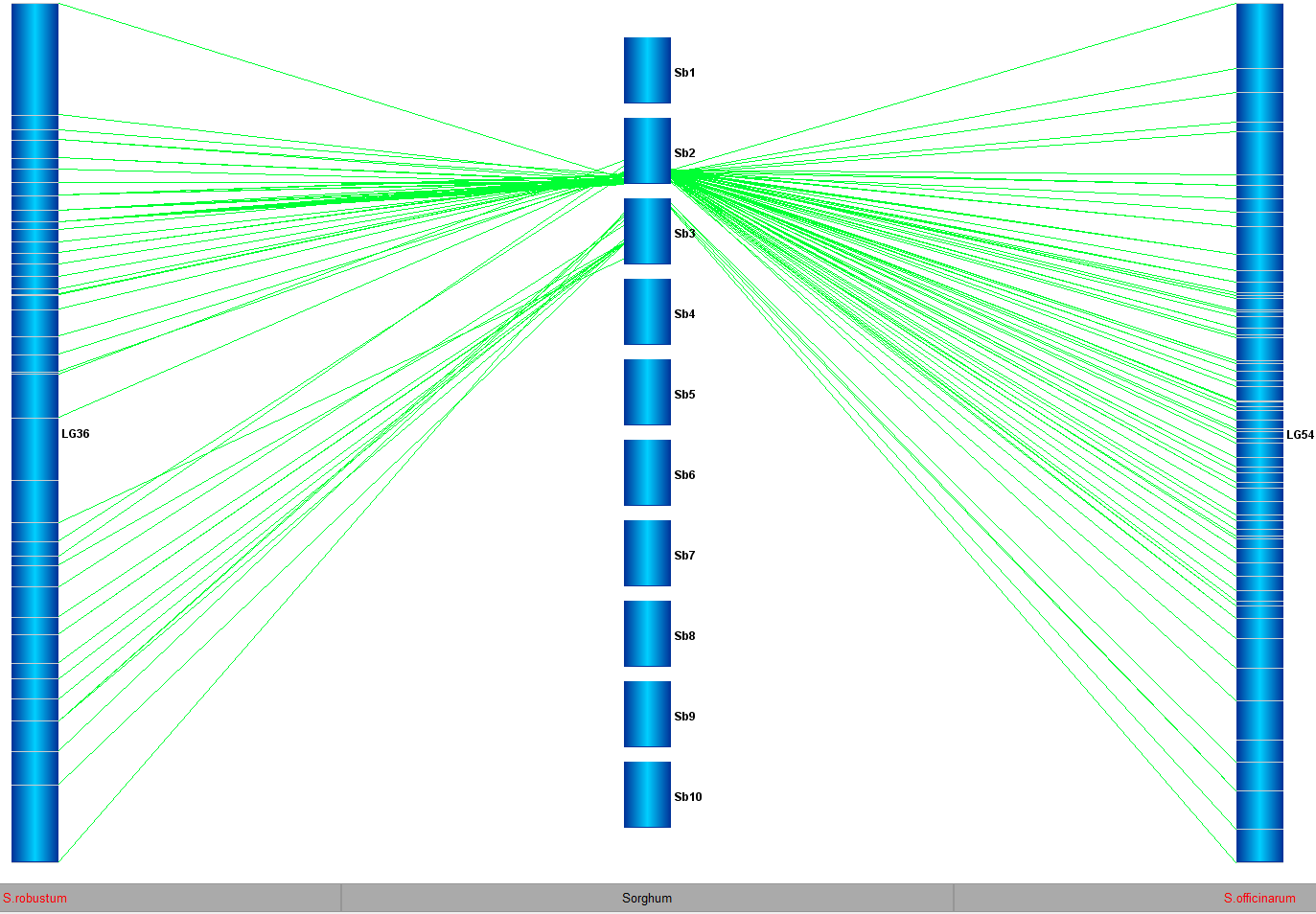


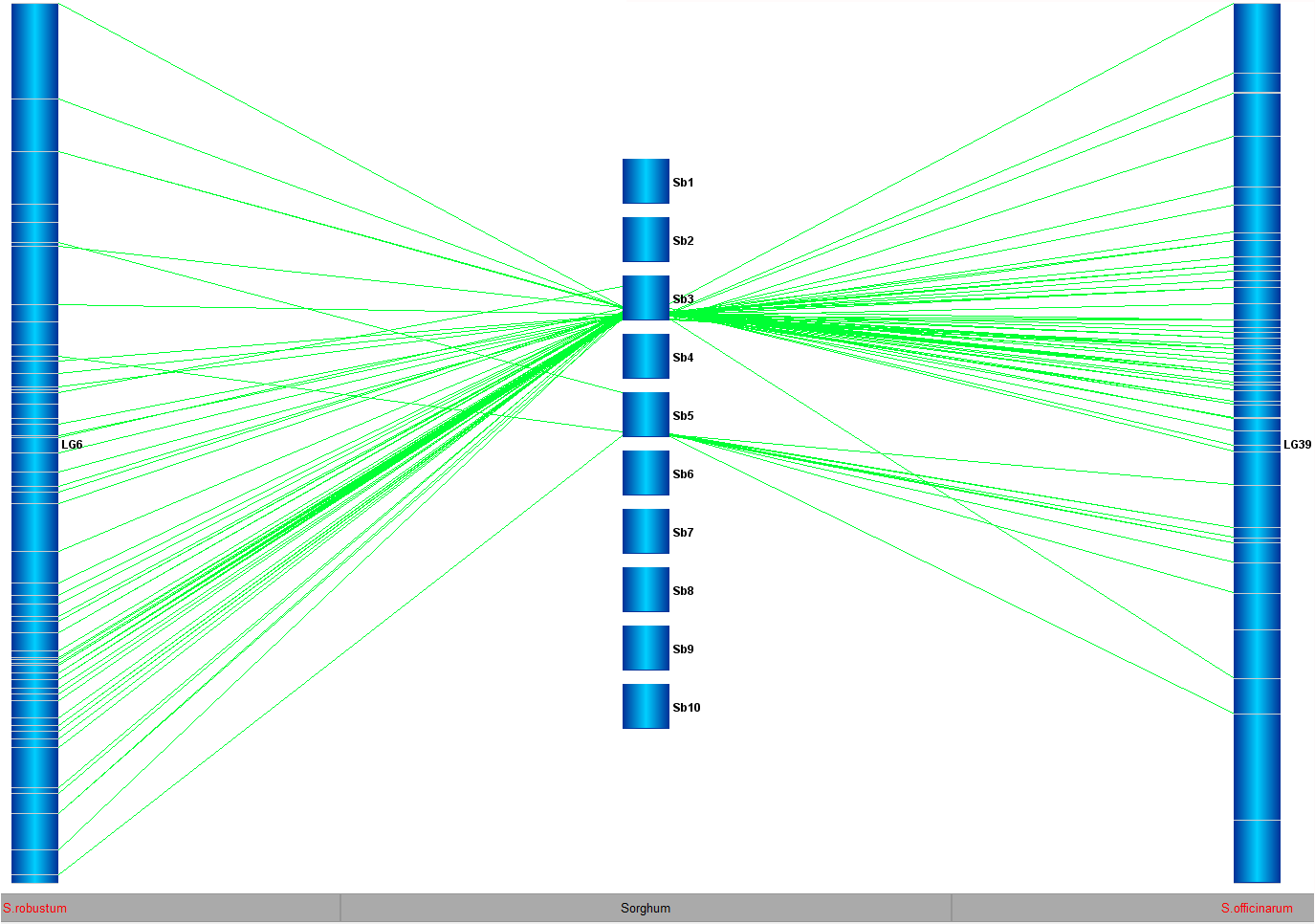

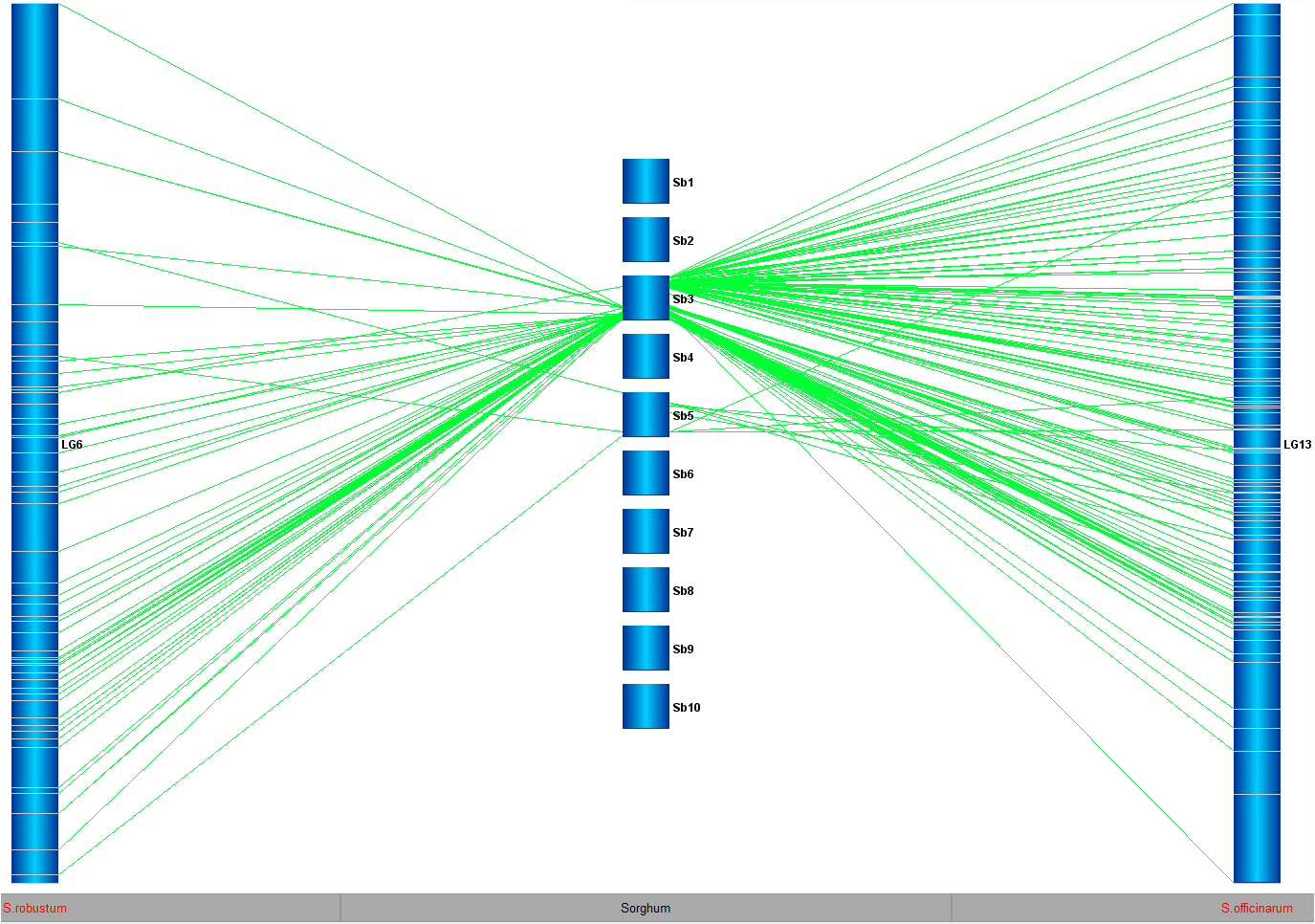

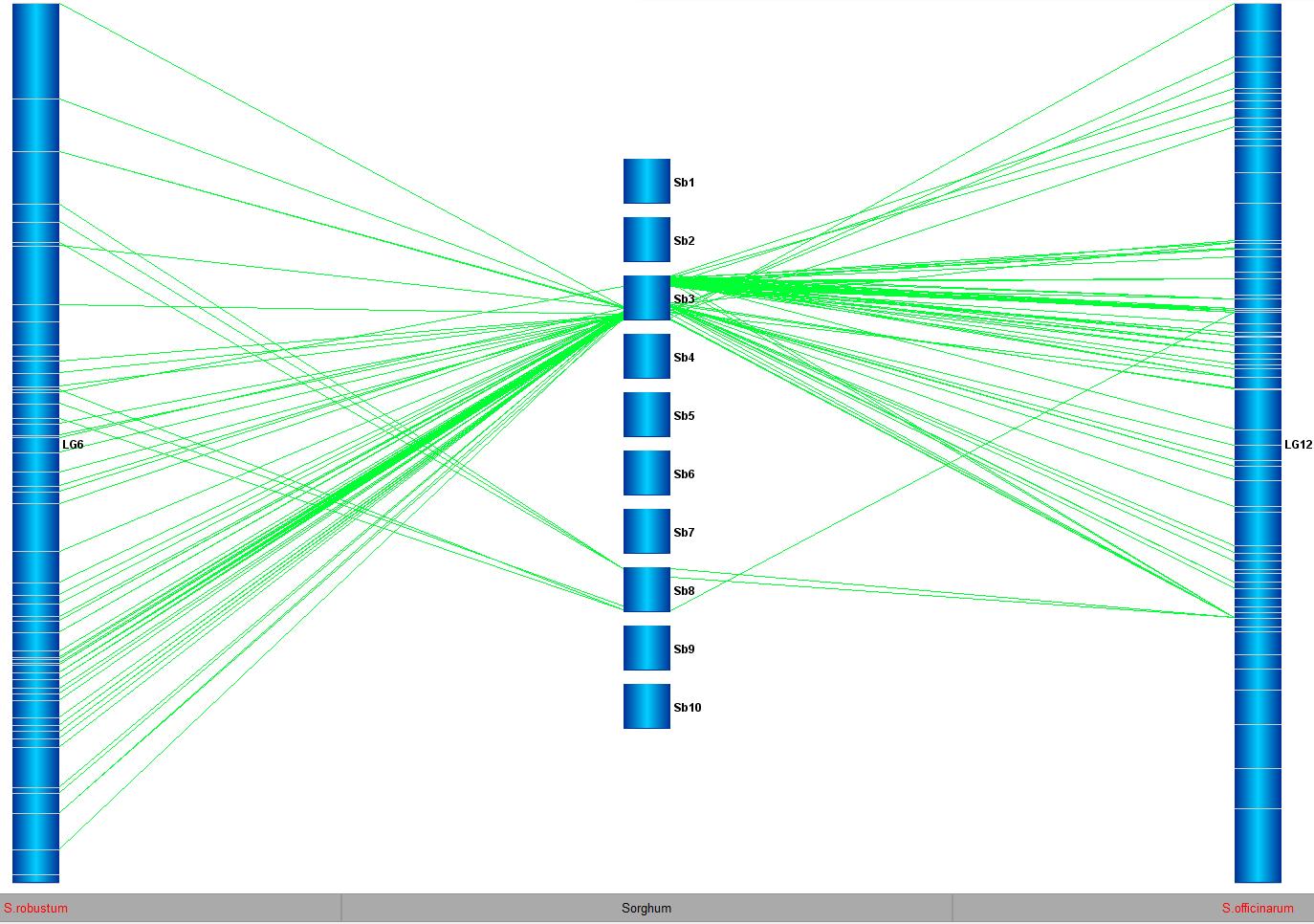

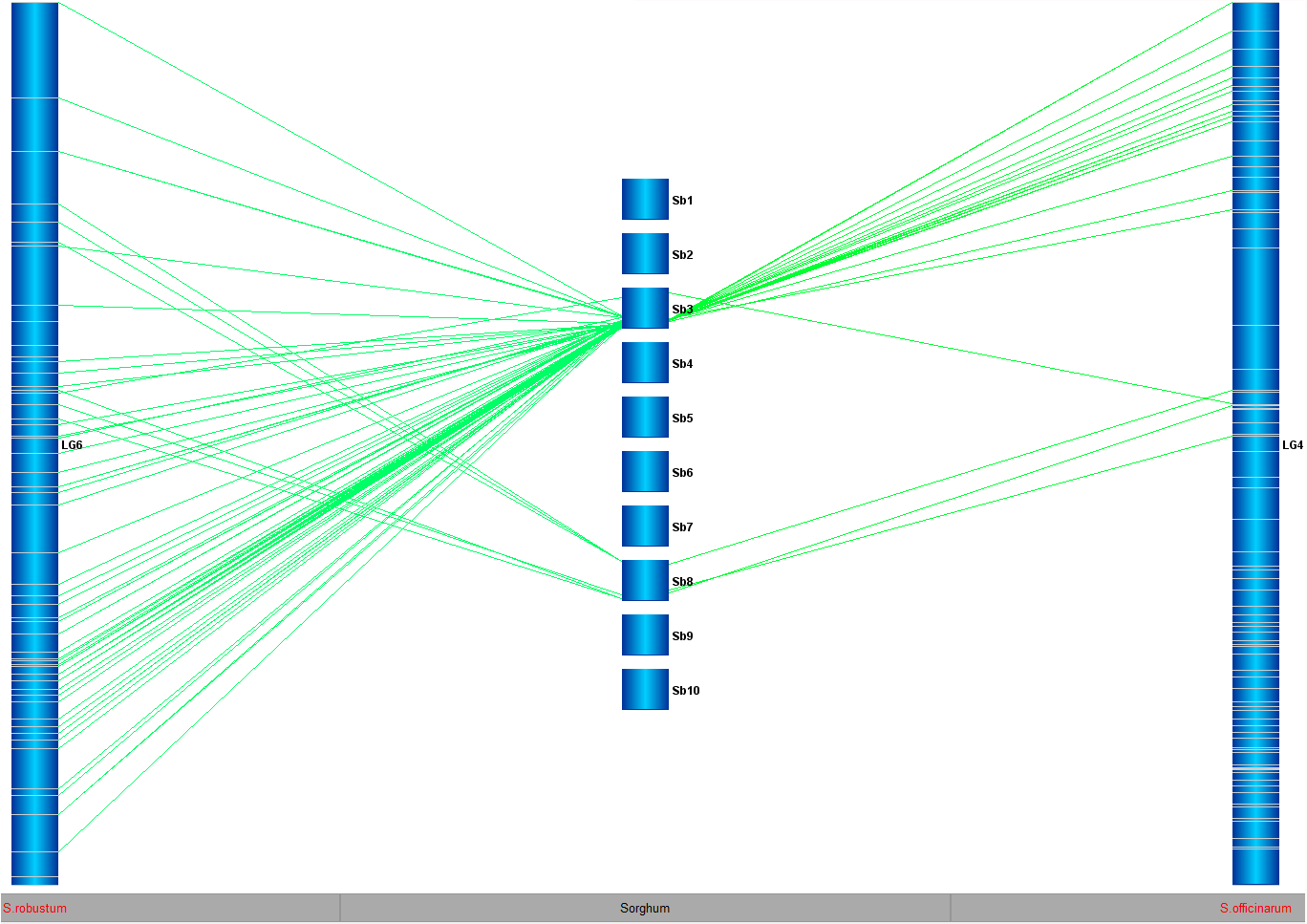


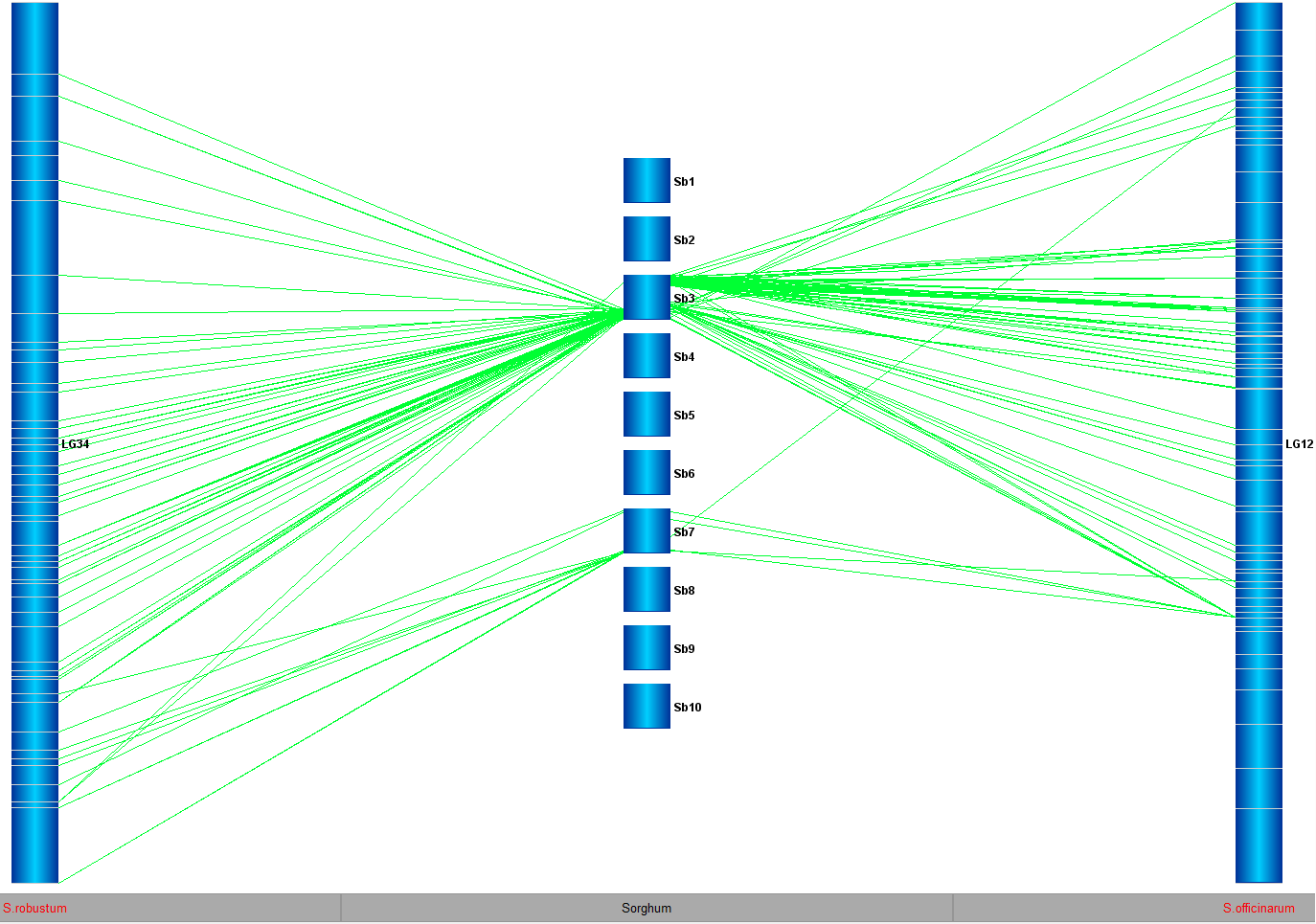


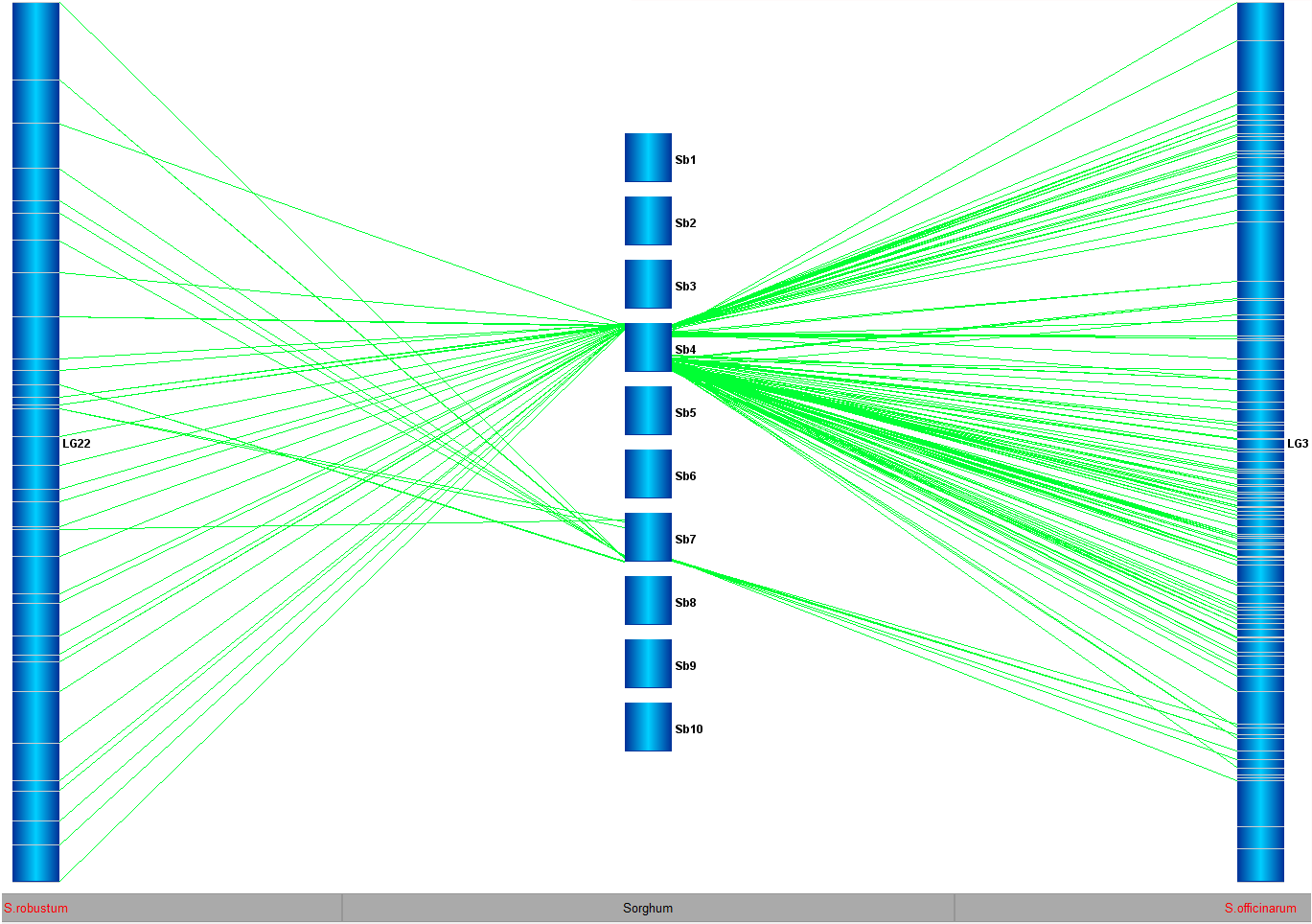


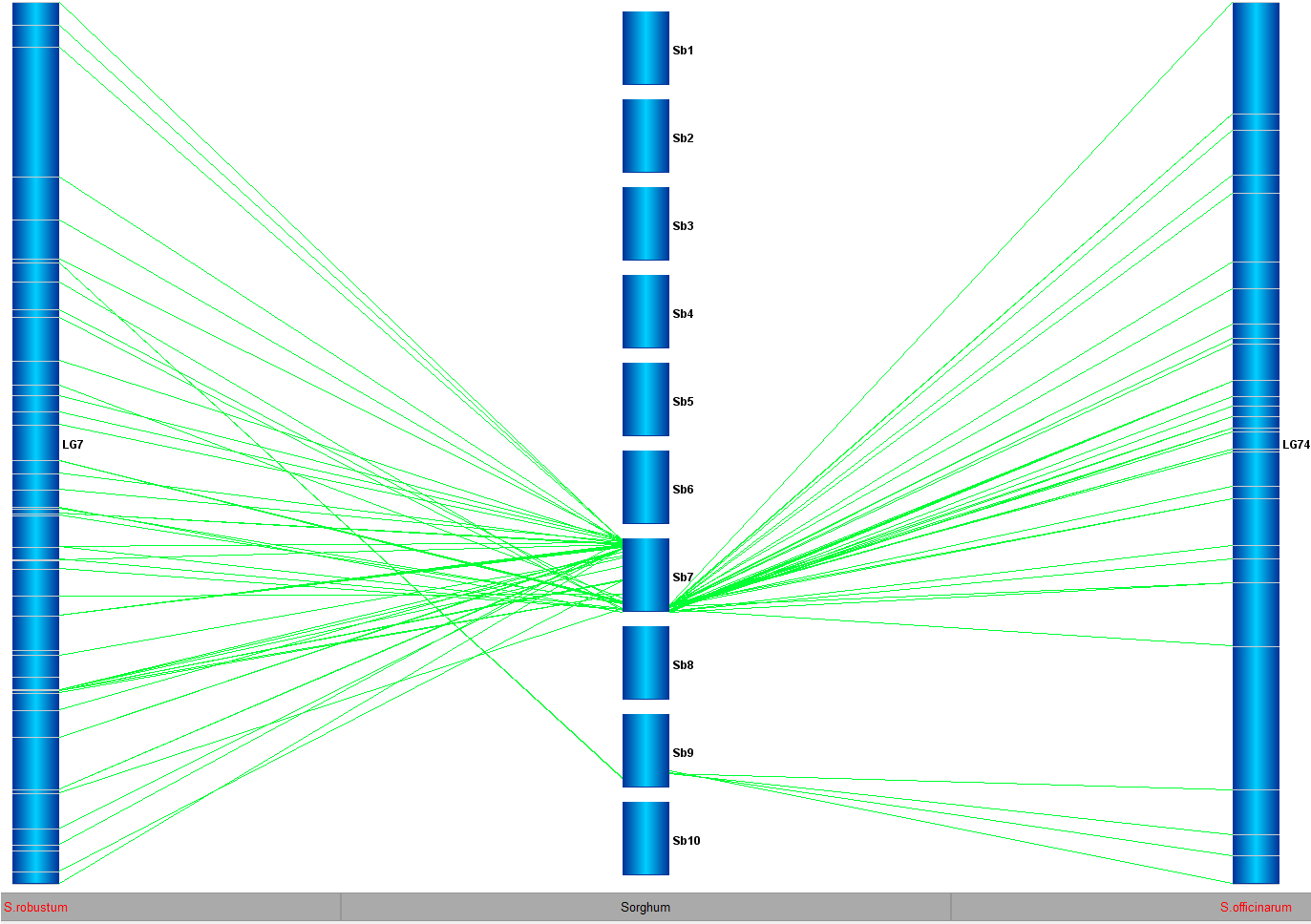


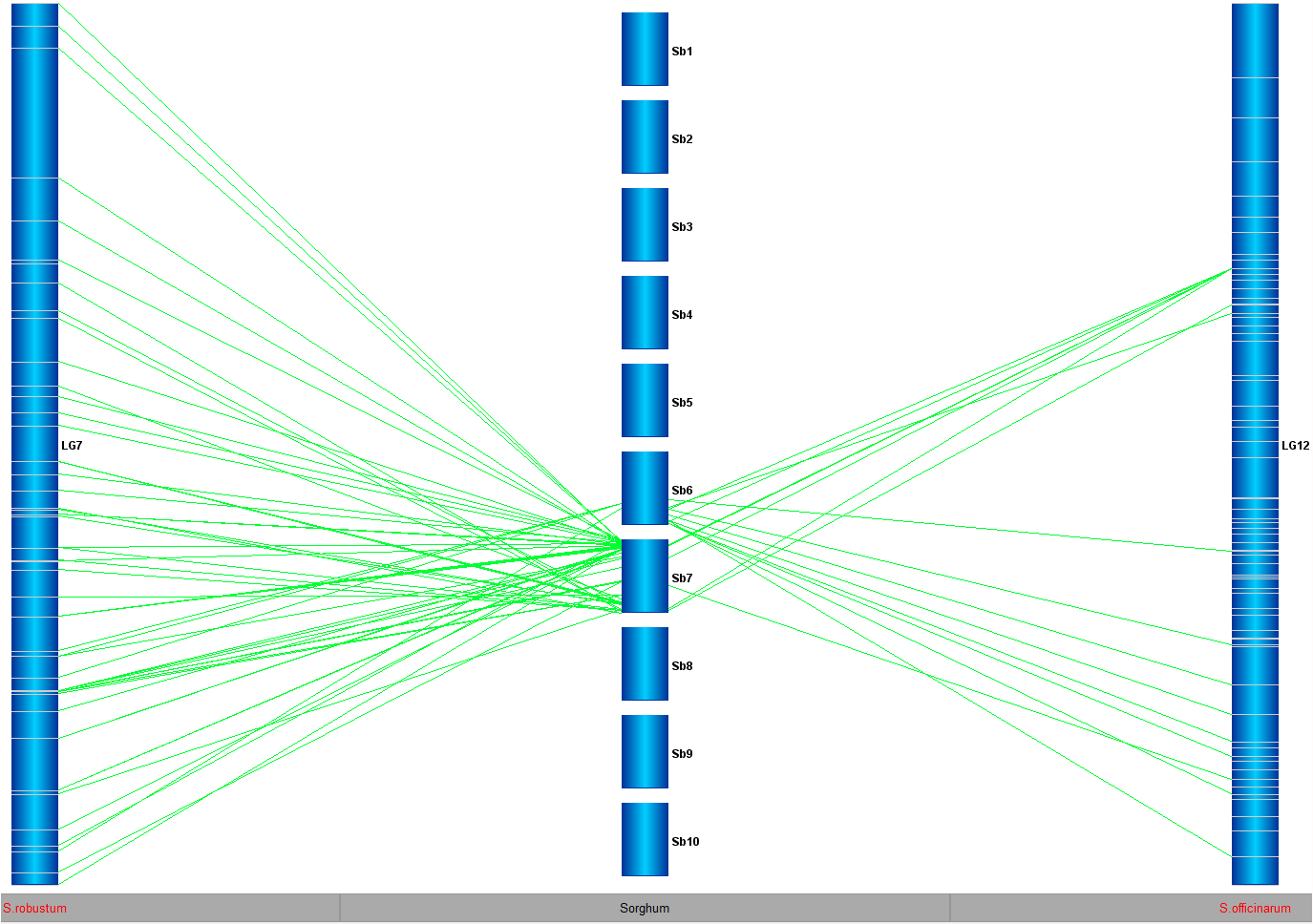


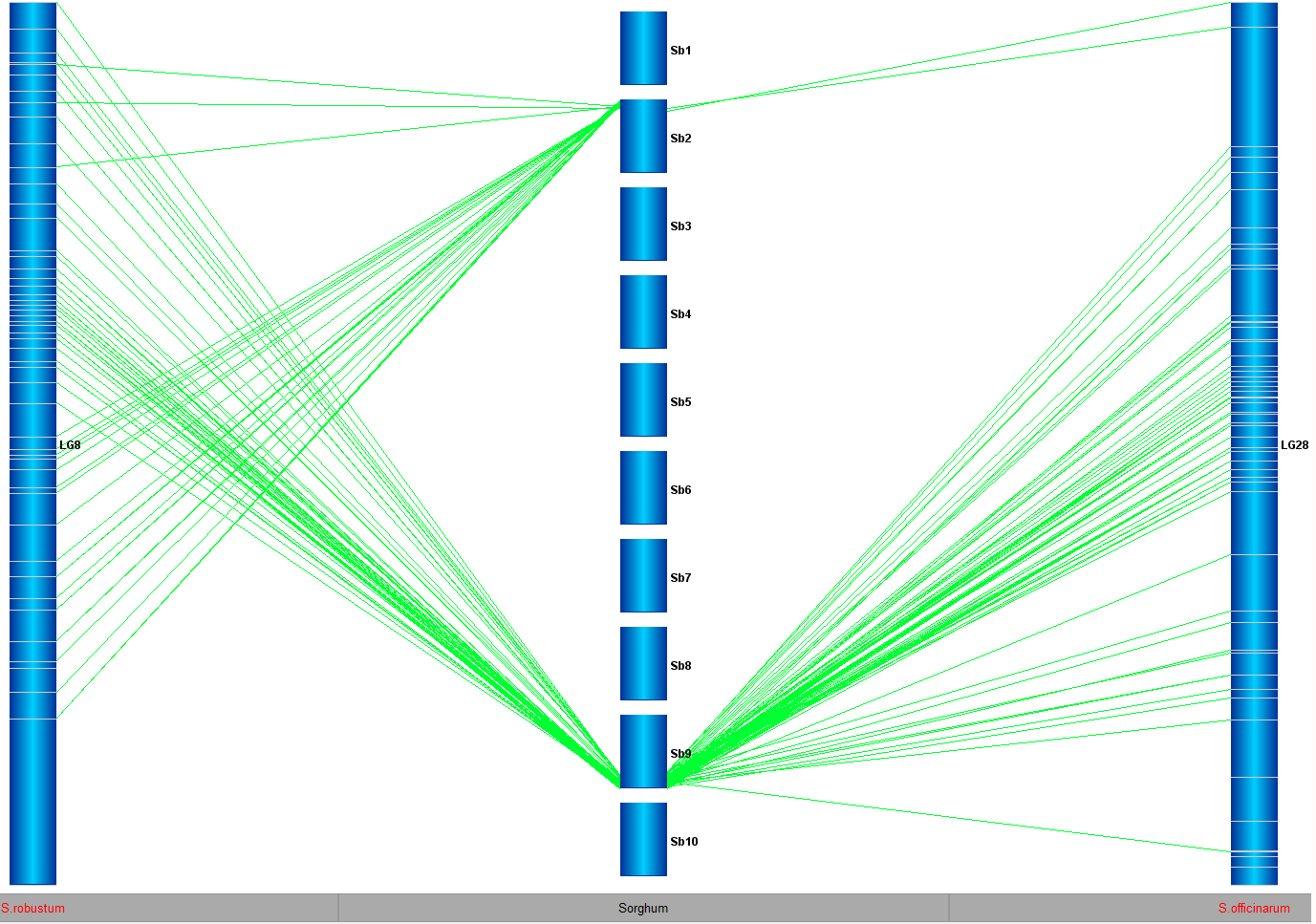


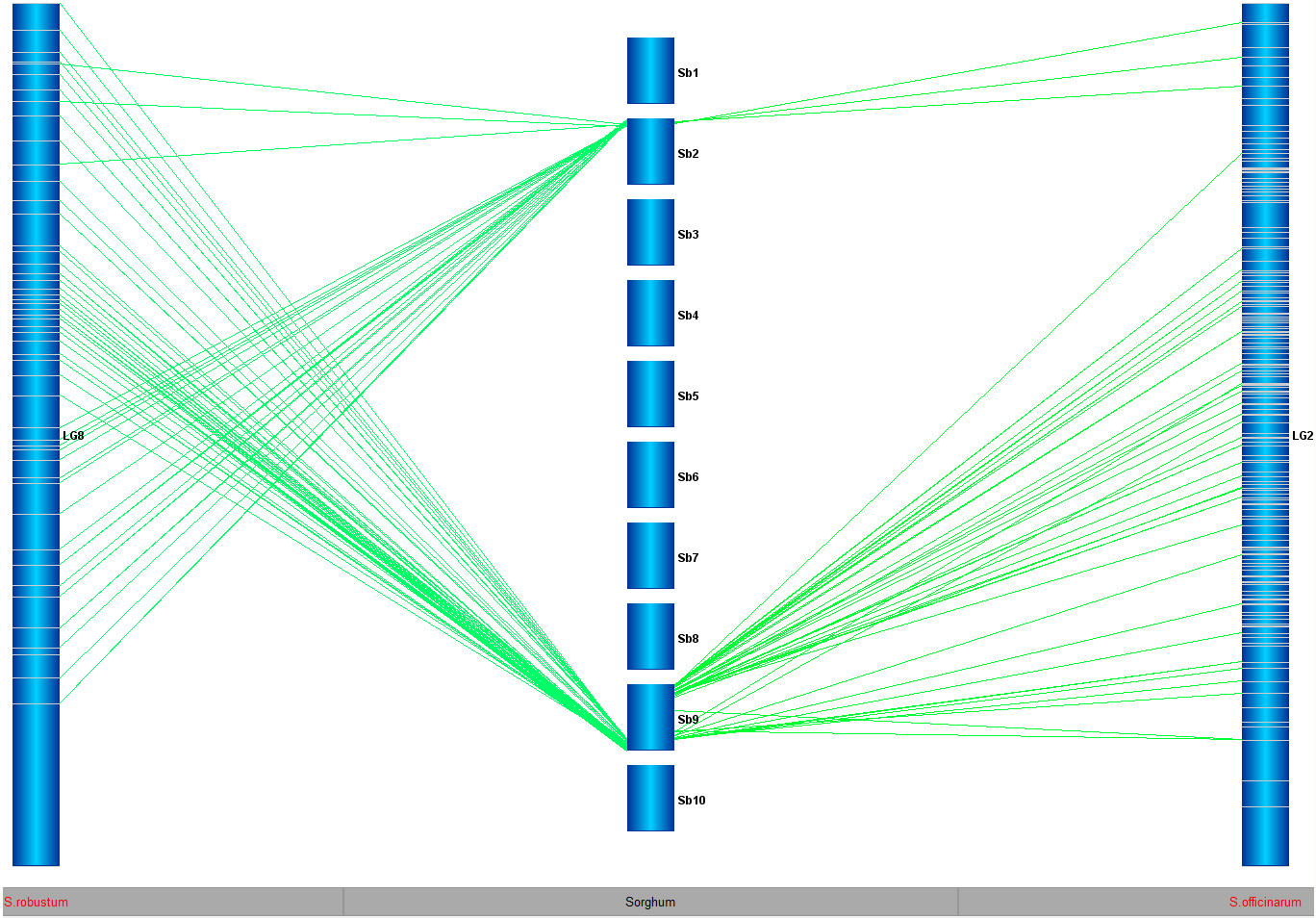


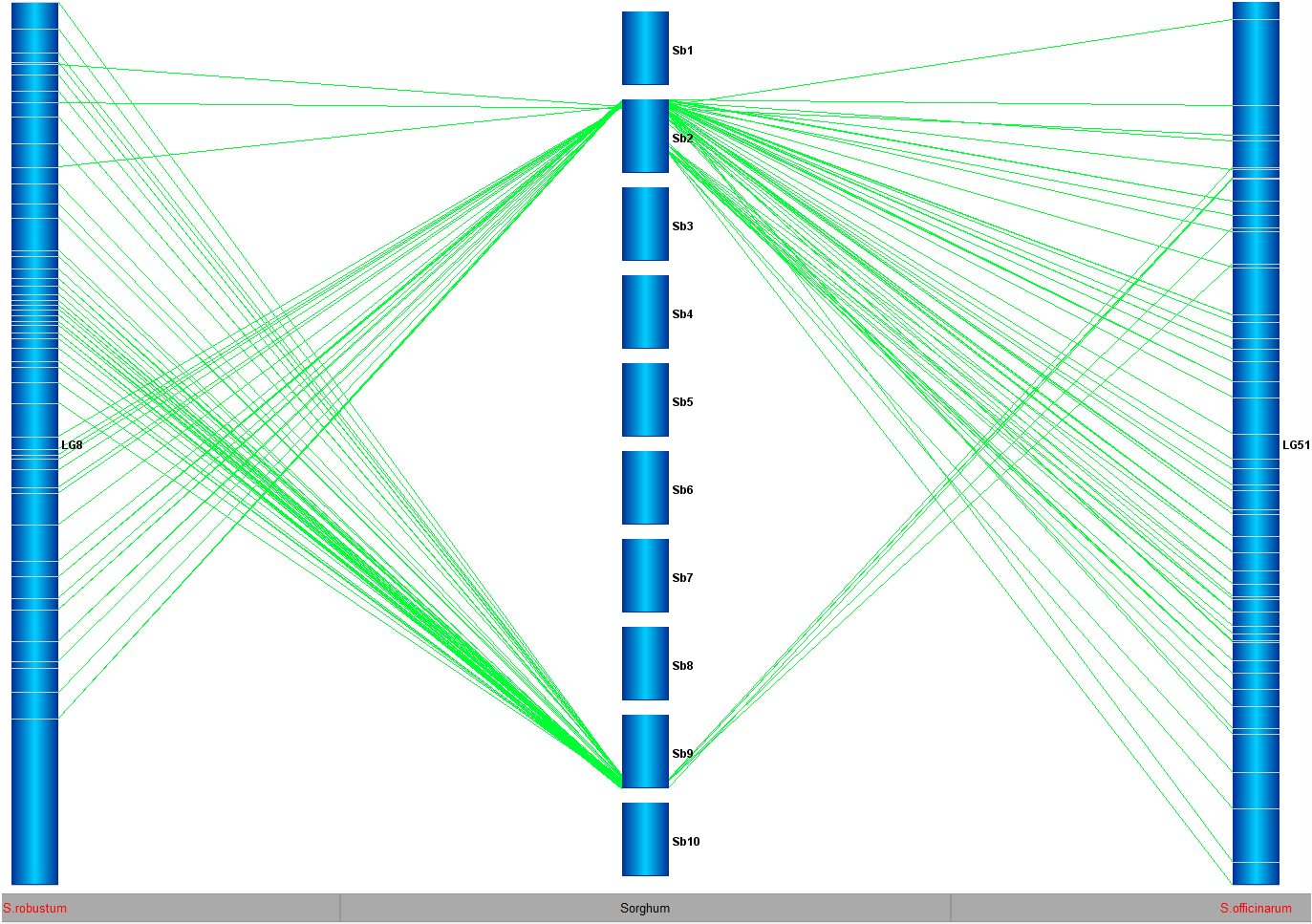

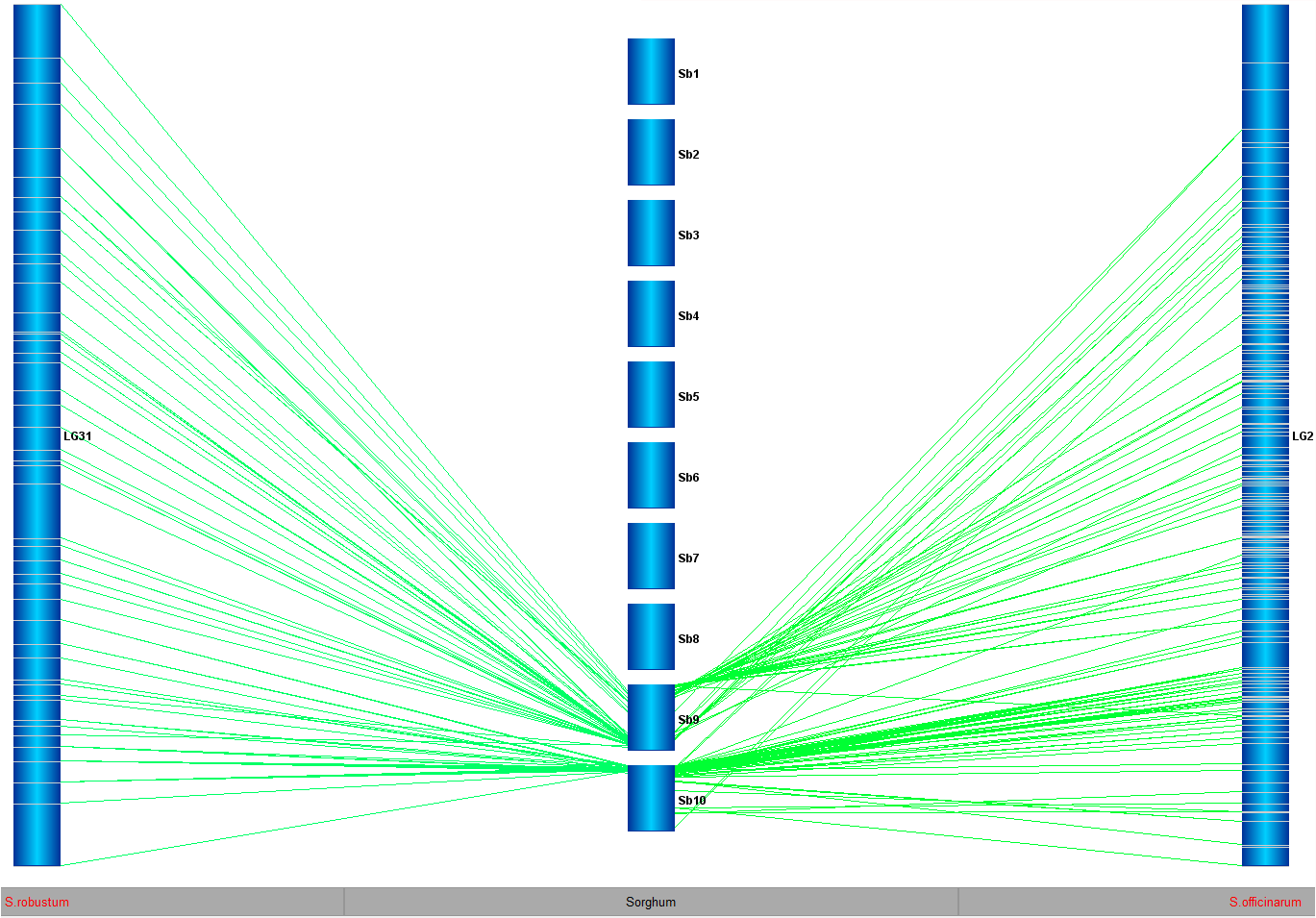


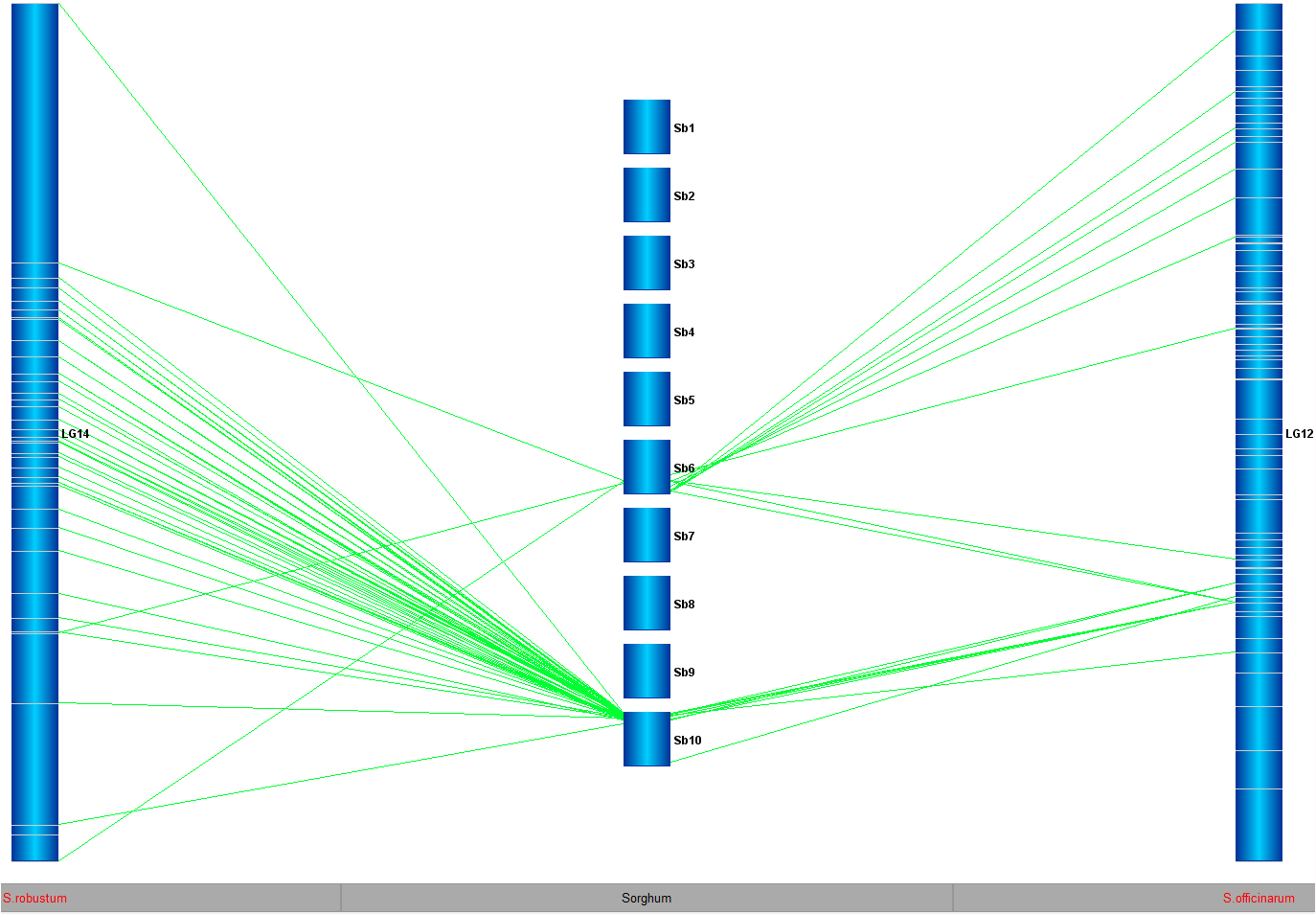


Supplemental Figure 7. Interchromosomal rearrangements between Saccharum and sorghum chromosomes

The red column indicated the linkage group of Saccharum , the blue column indicated the sorghum chromosomes. The green color indicated the r


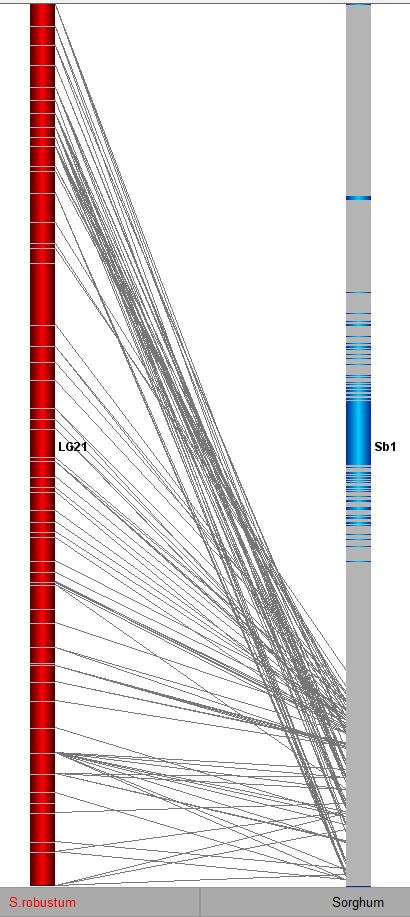

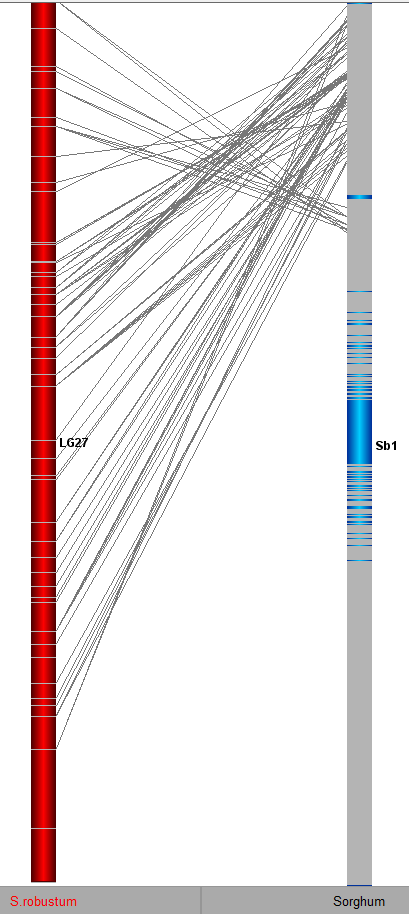

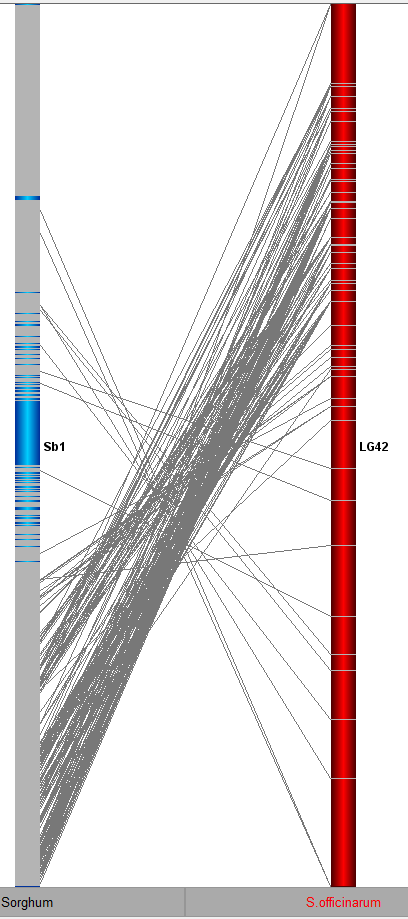


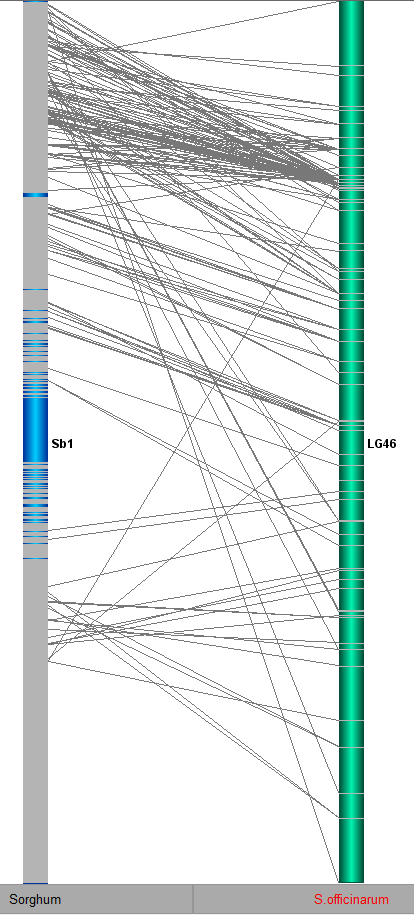

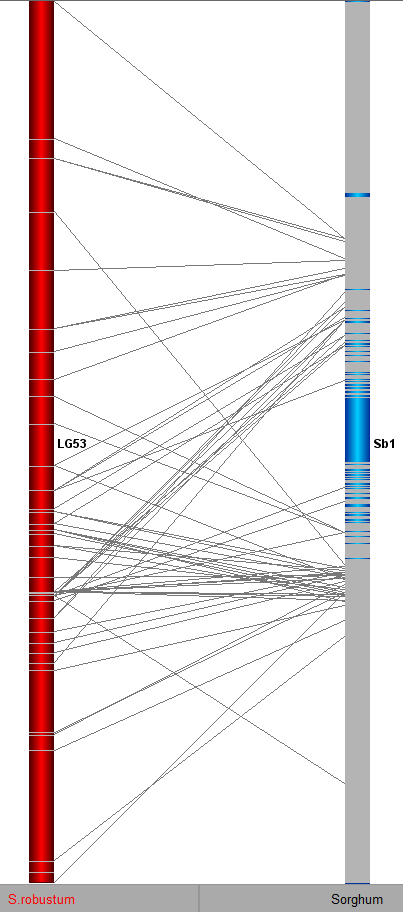

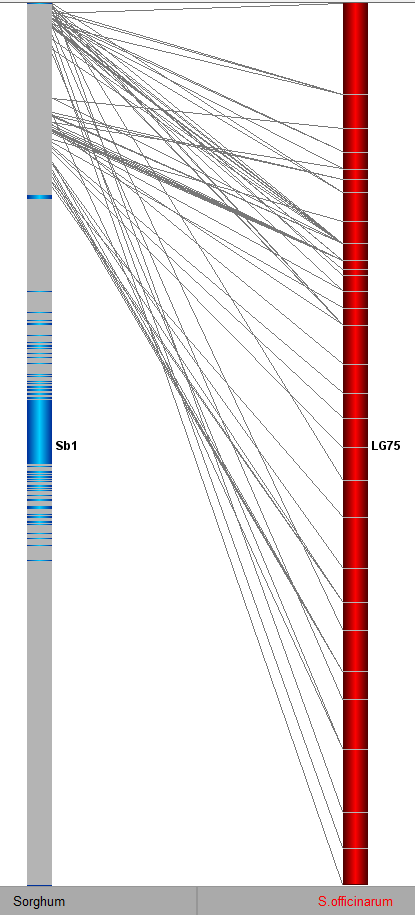

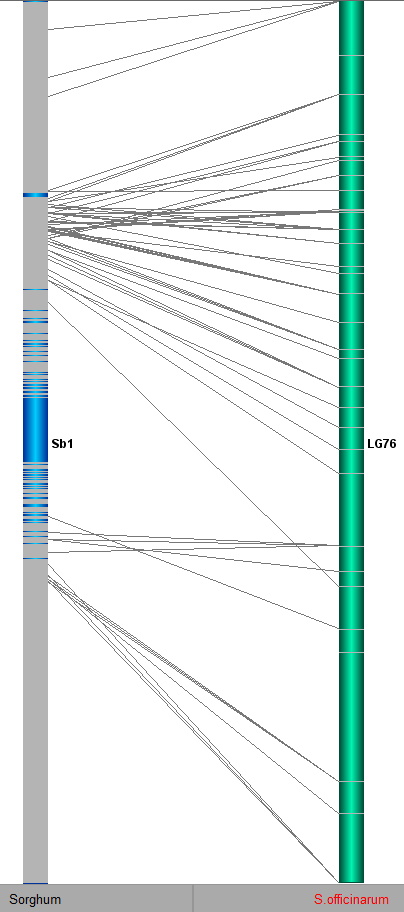


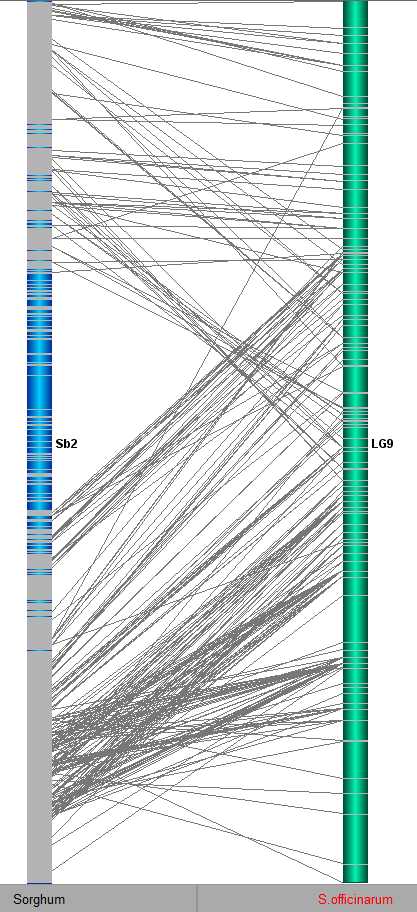

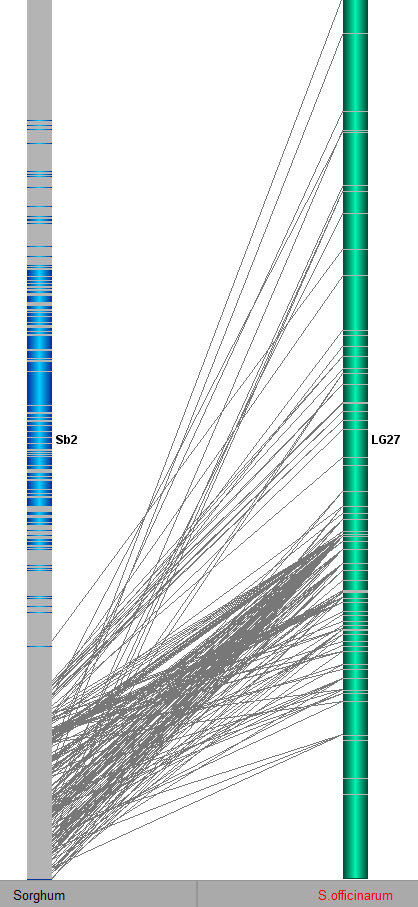

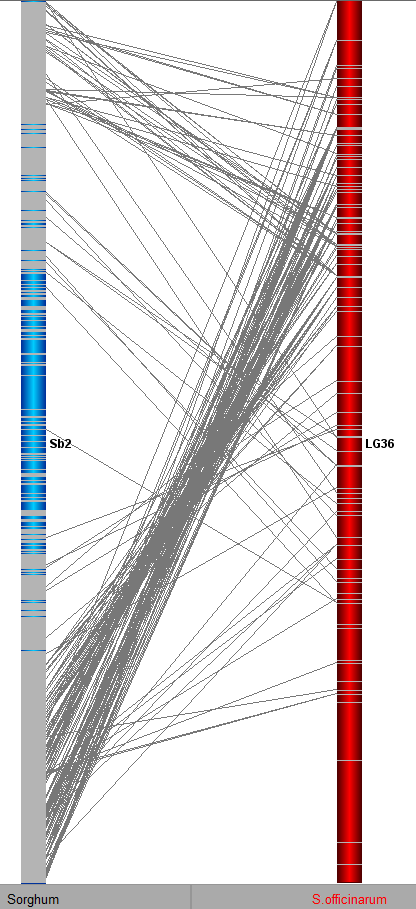


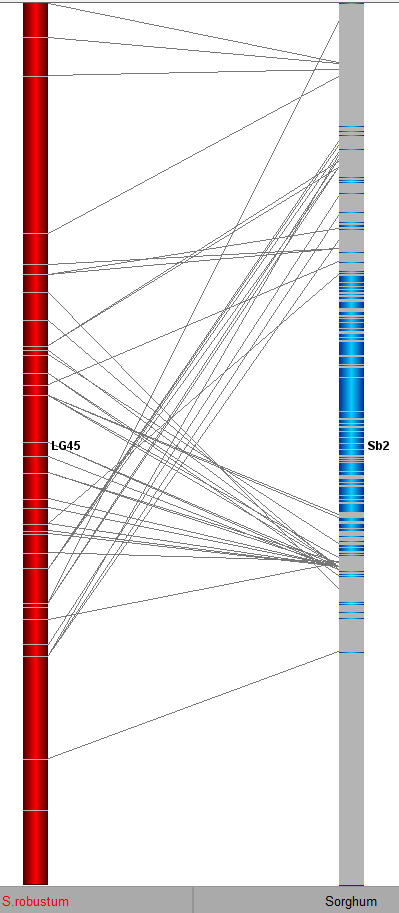

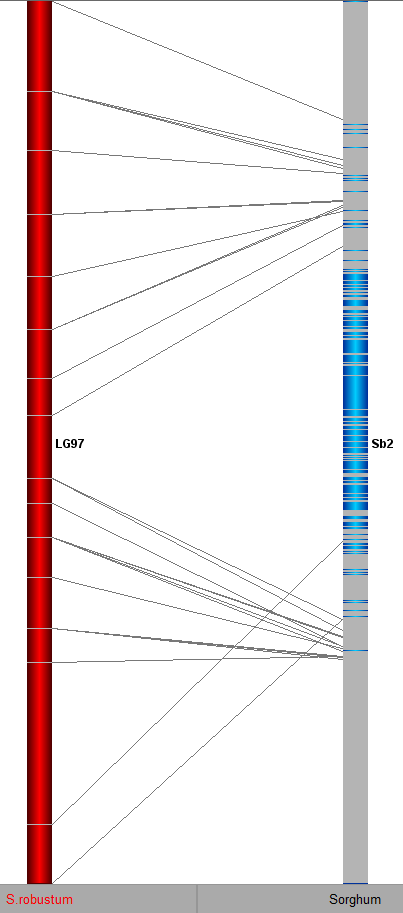


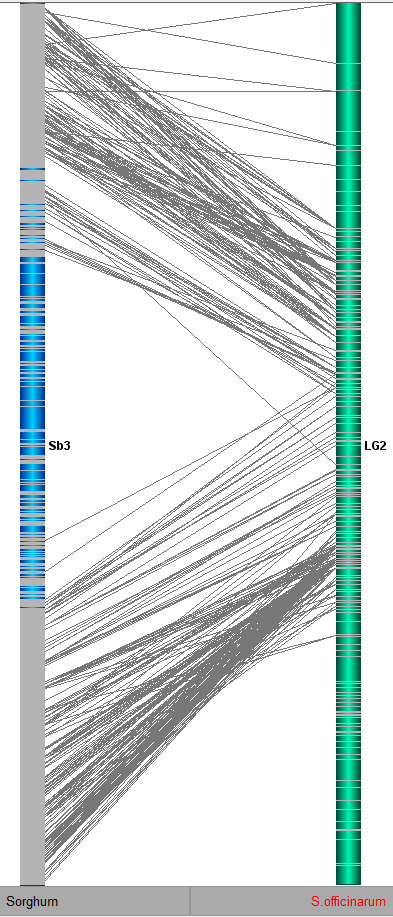

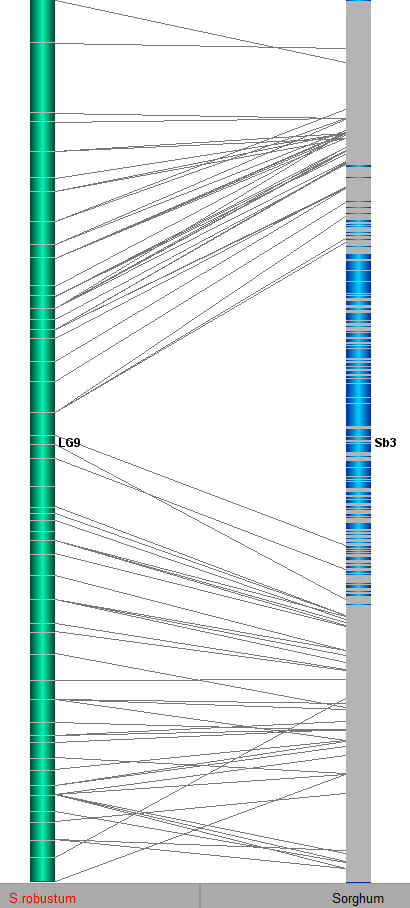

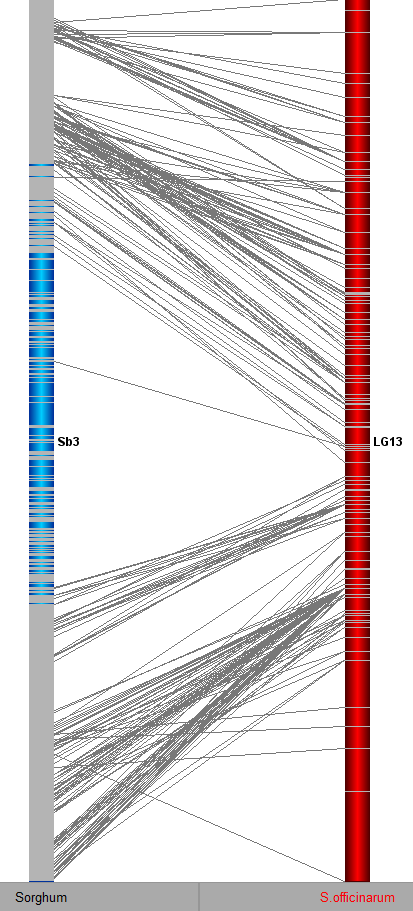


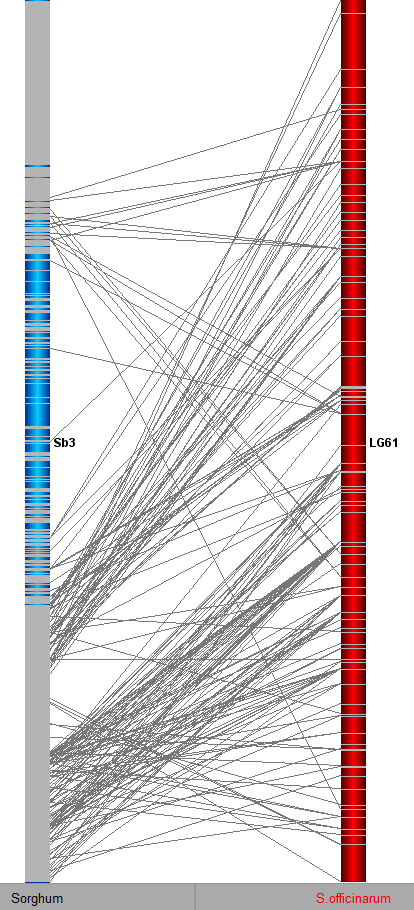


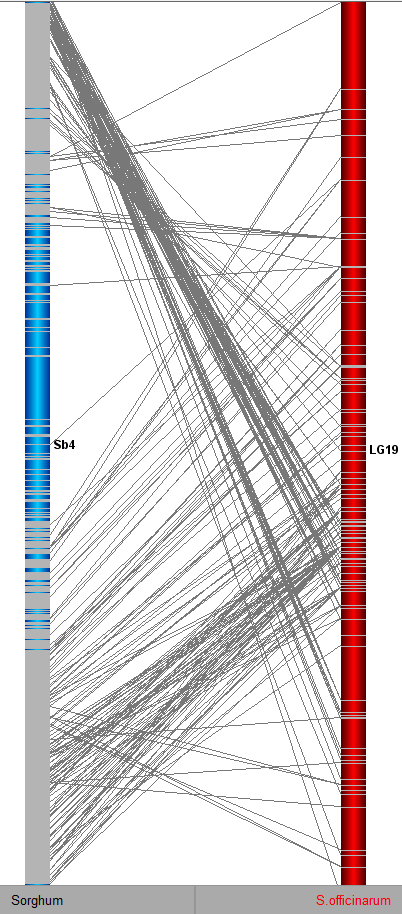

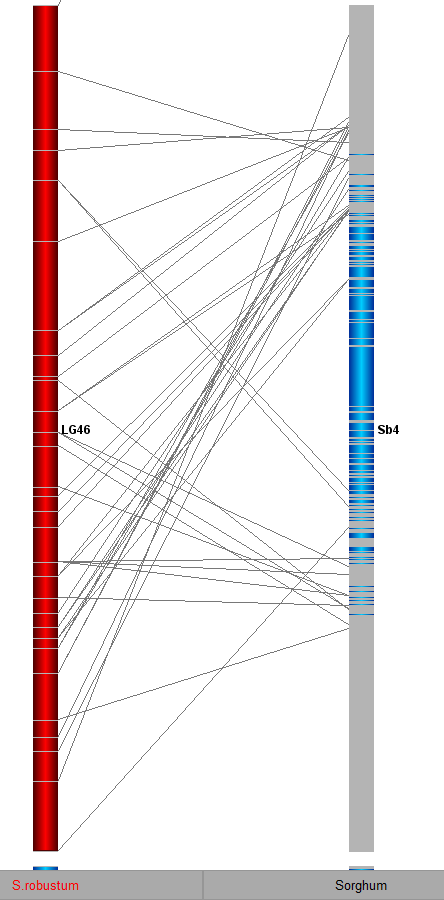

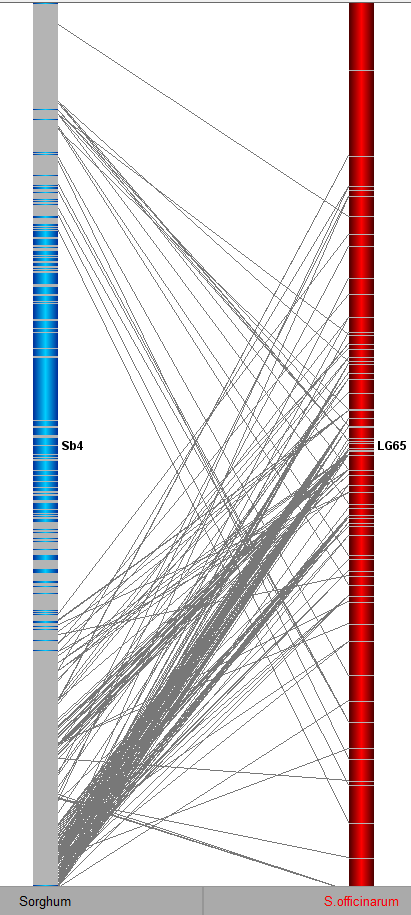


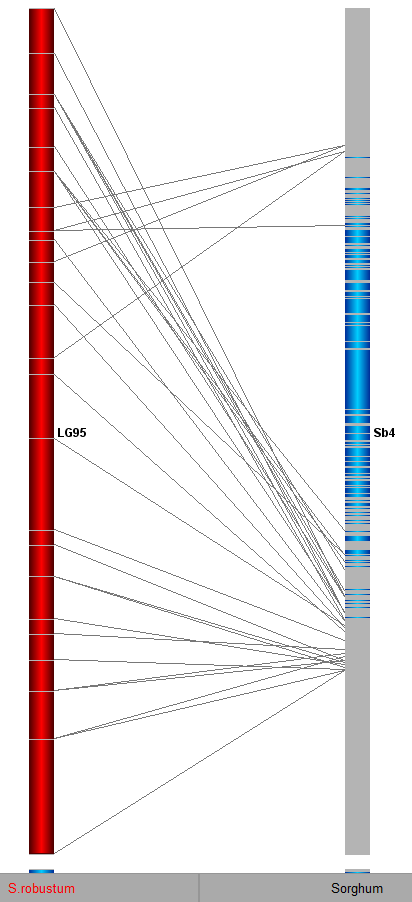


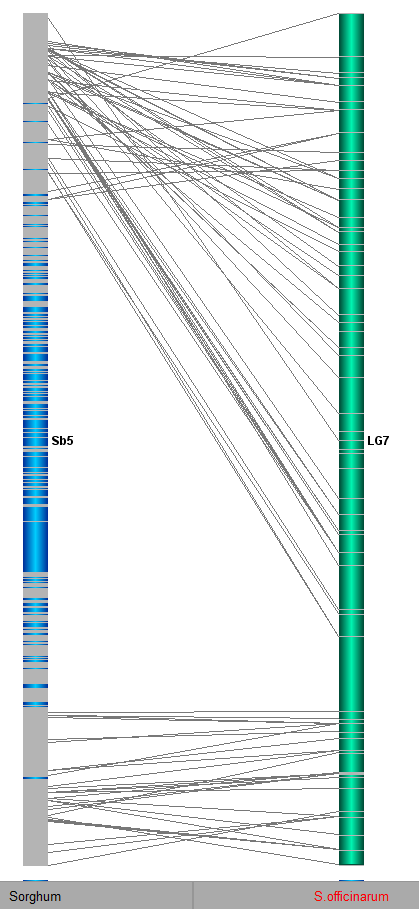

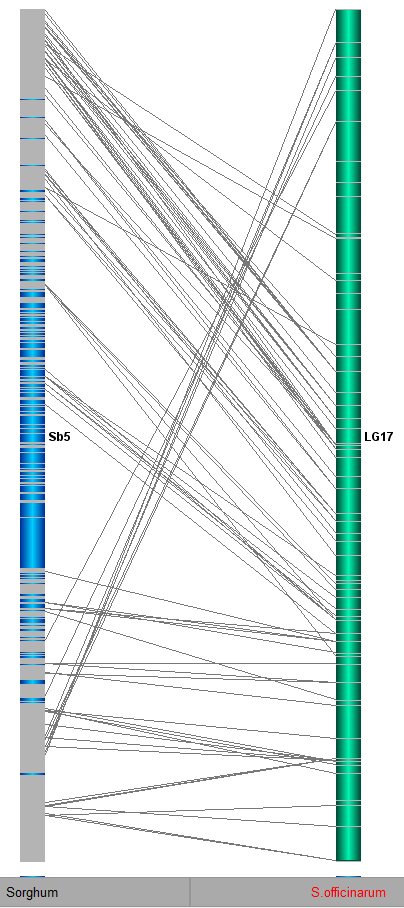

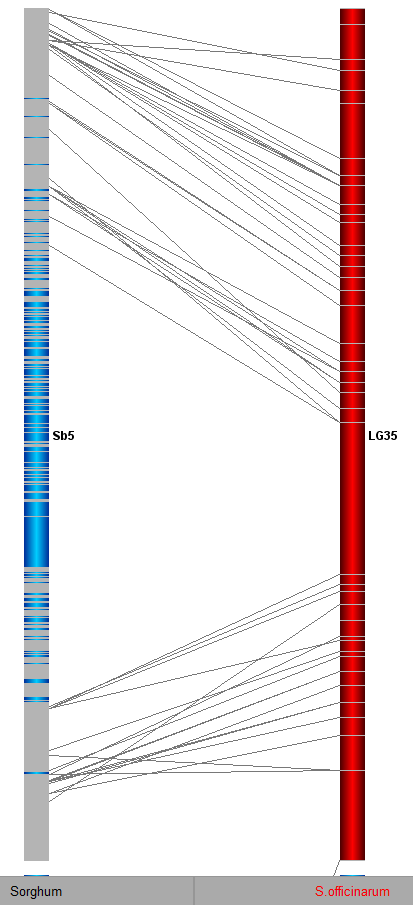


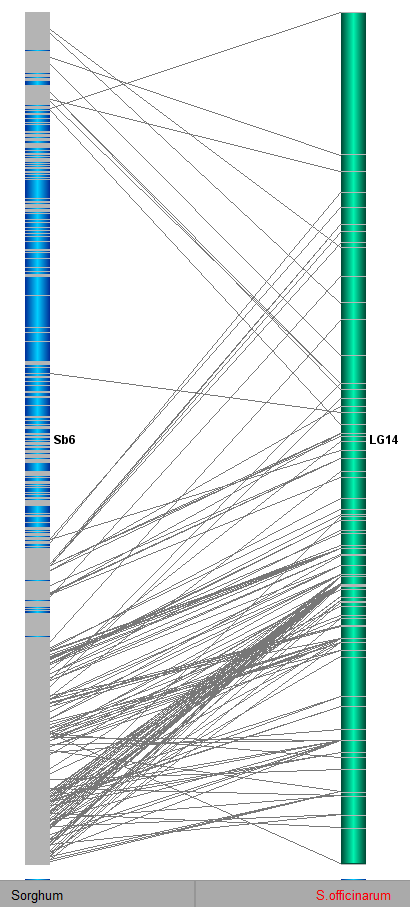

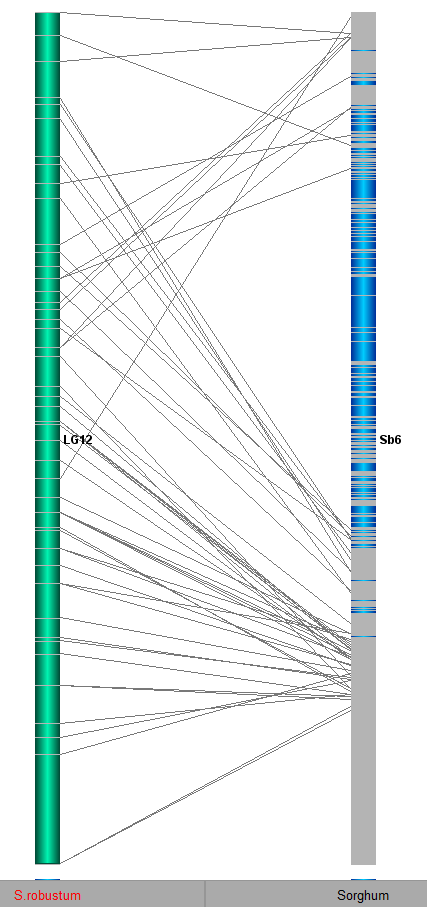

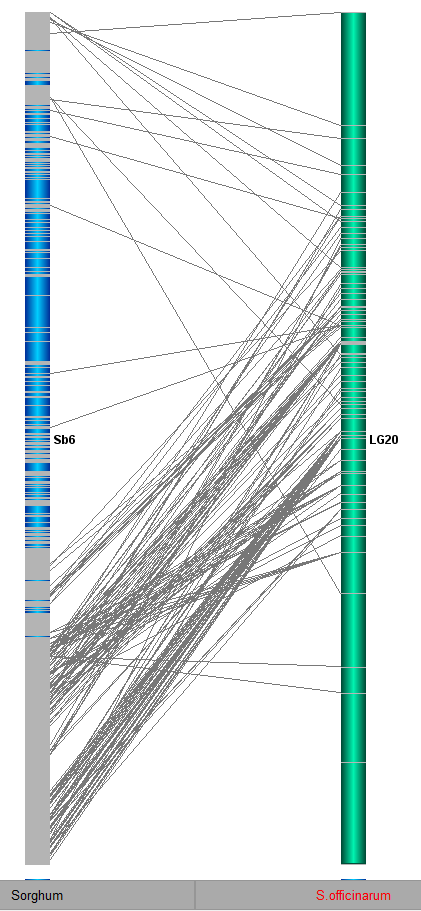

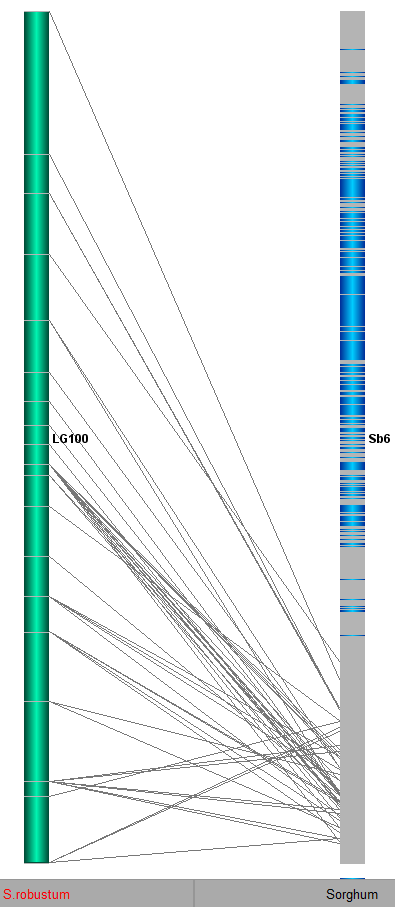


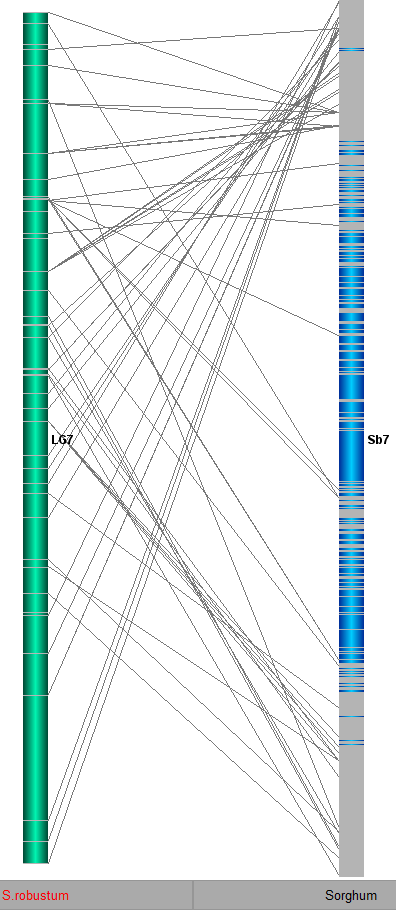

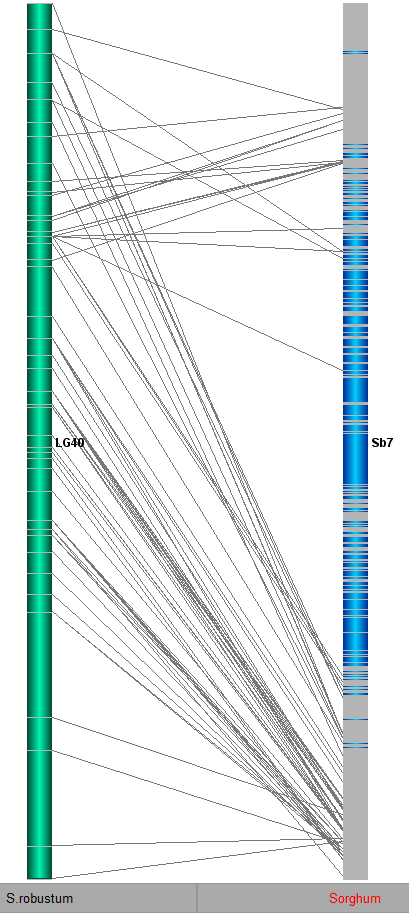

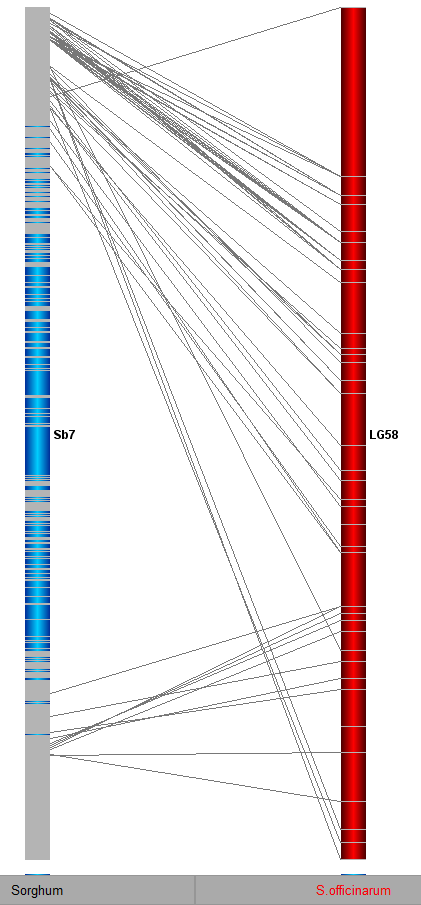


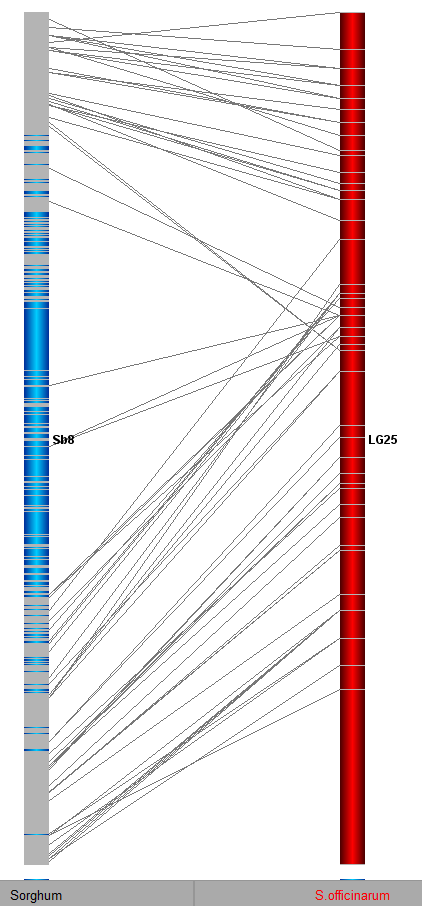


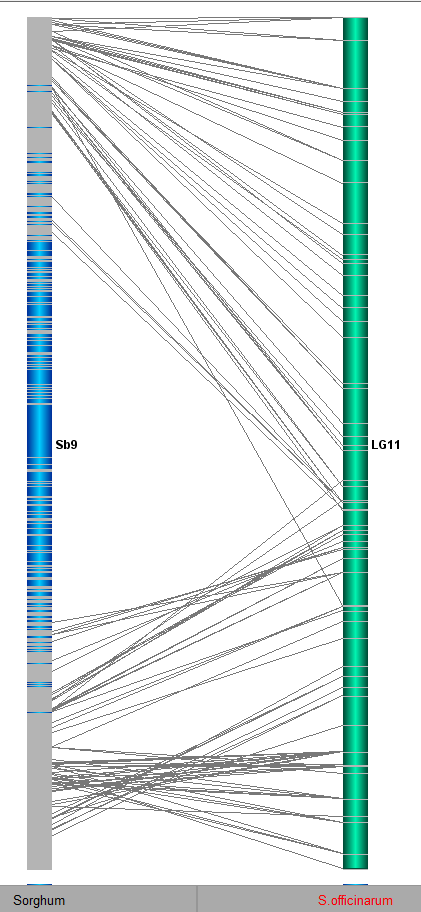


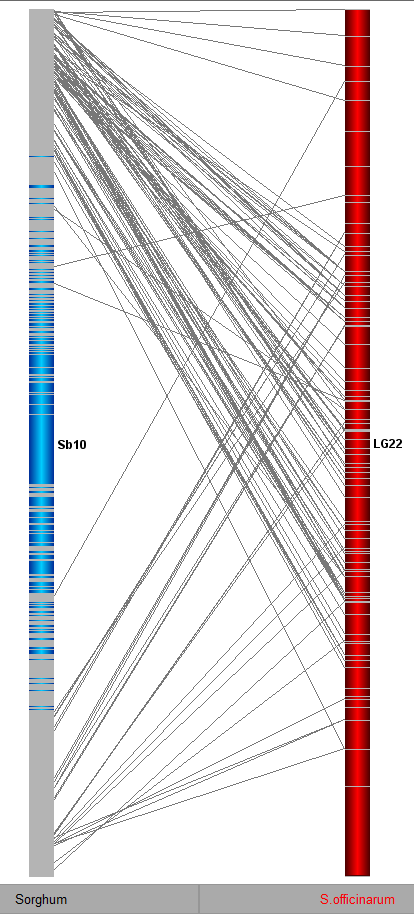

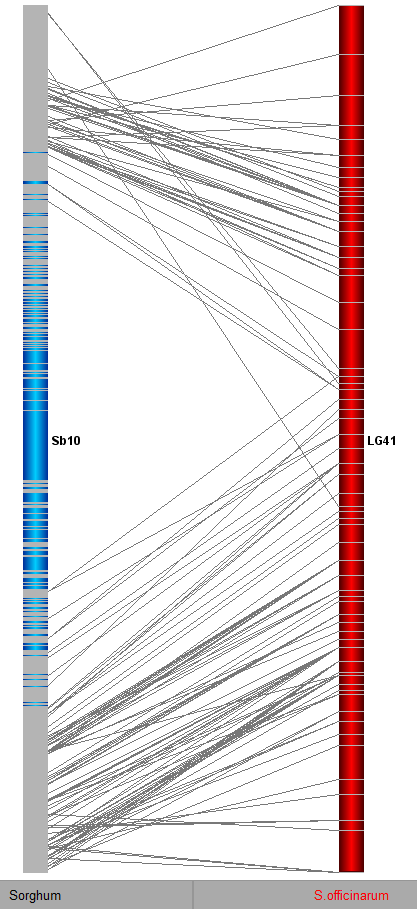

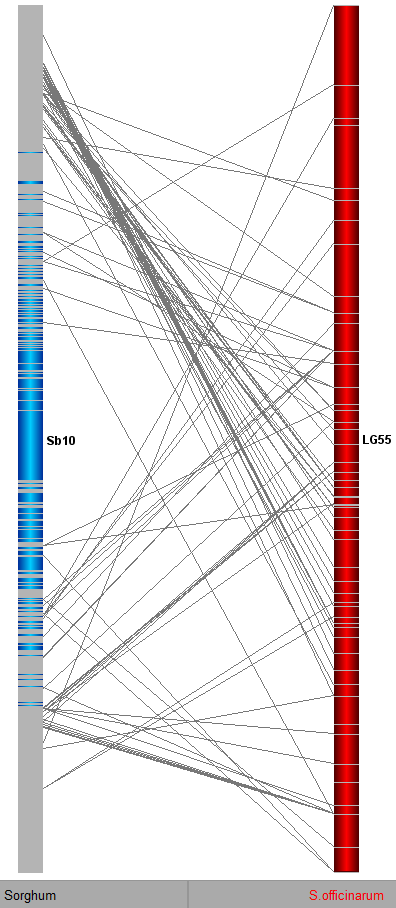

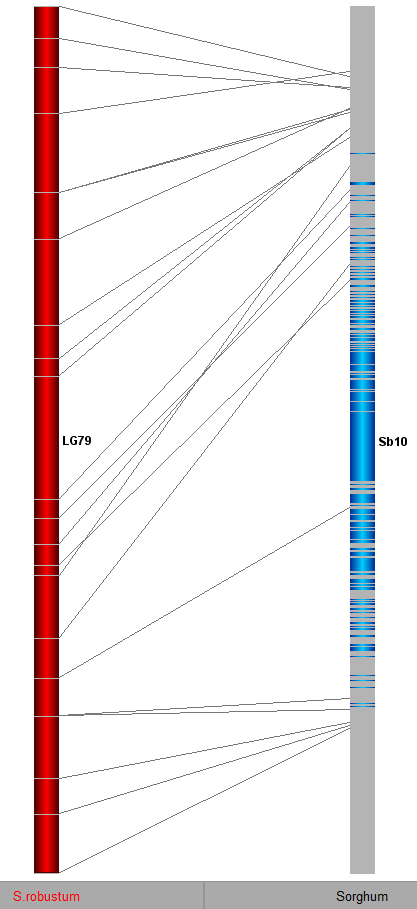


Supplemental Figure 8:. Intrachromosomal rearrangements of homologous group among Saccharum chromosomes.


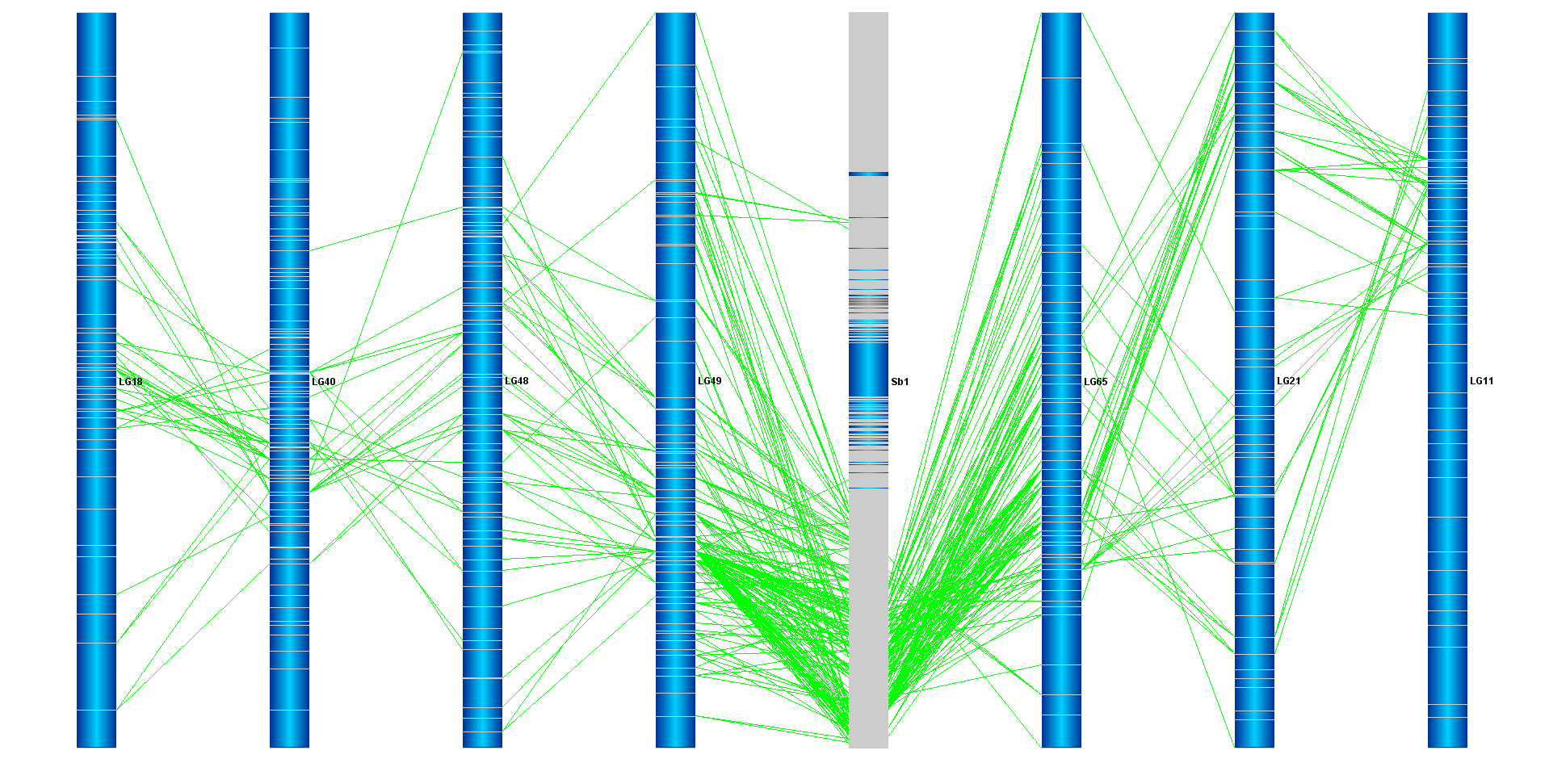


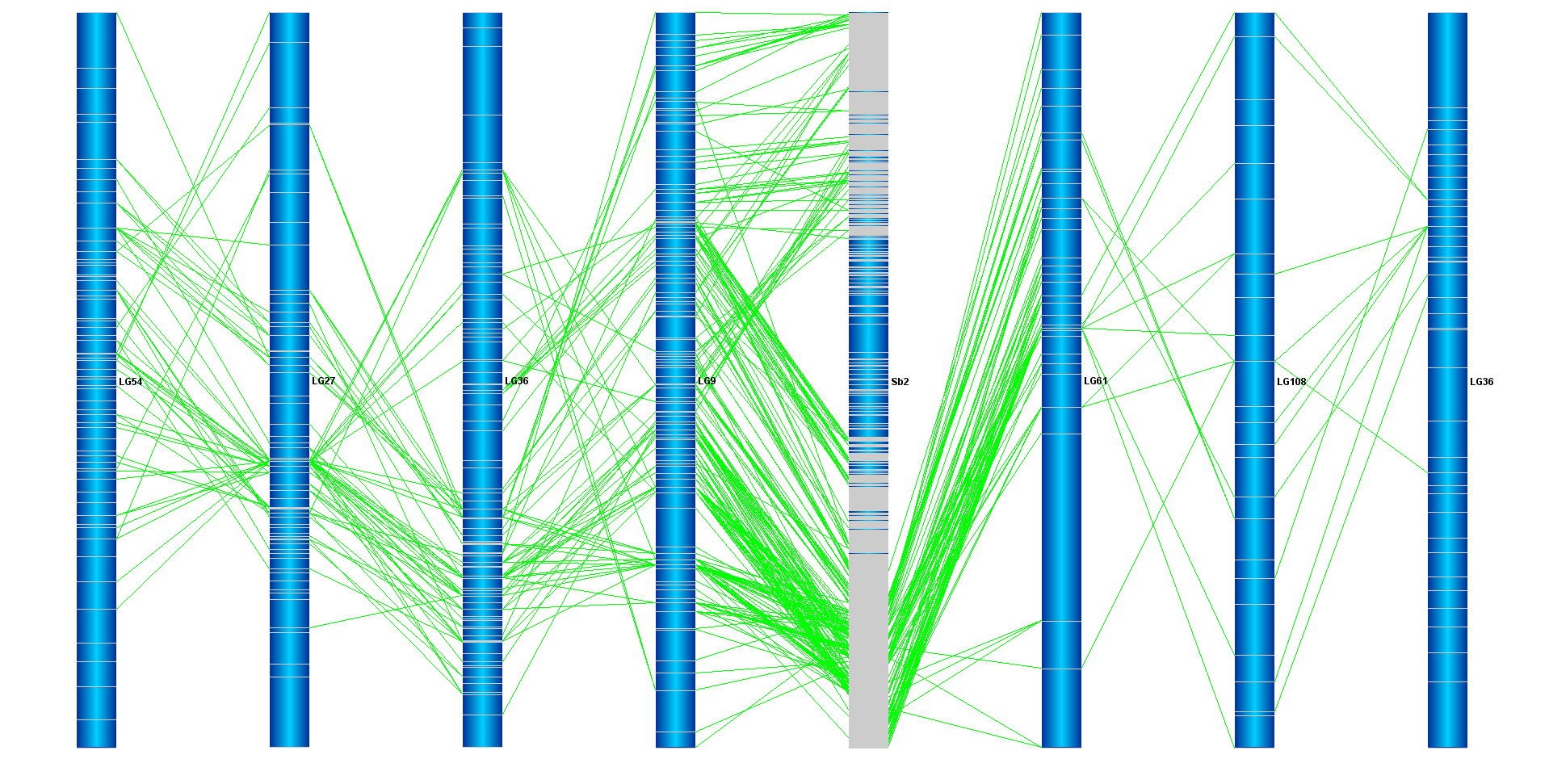


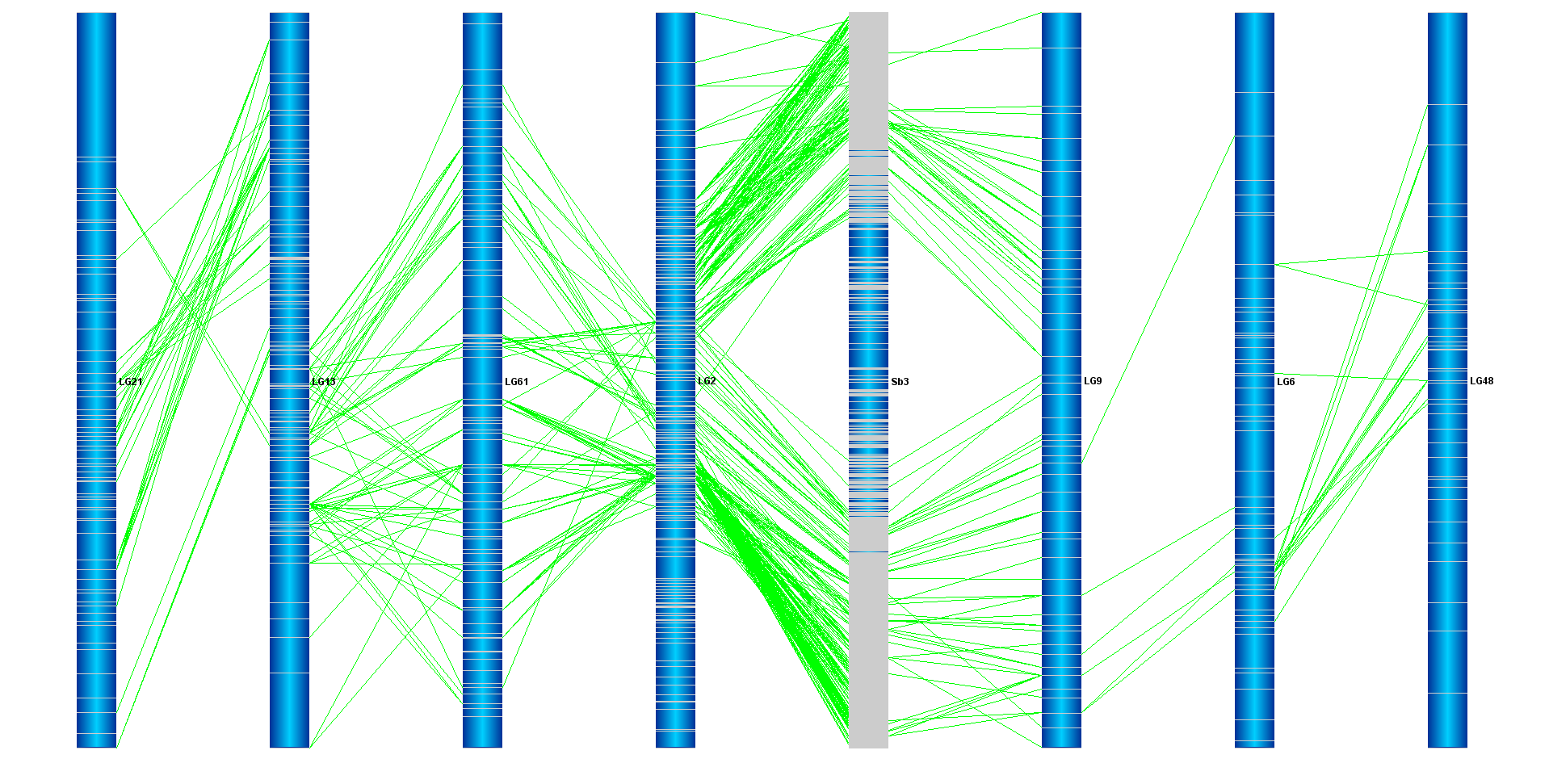


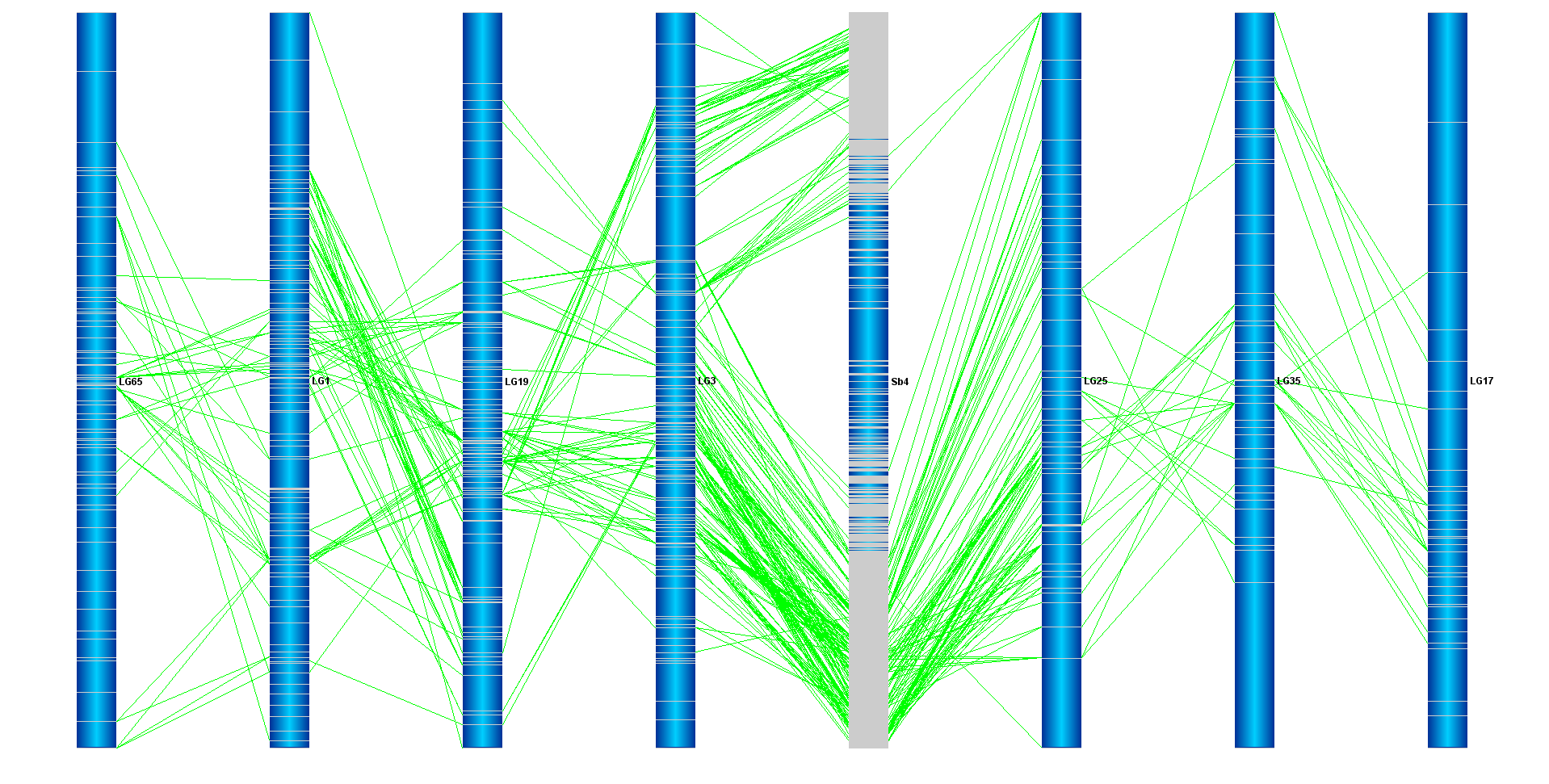

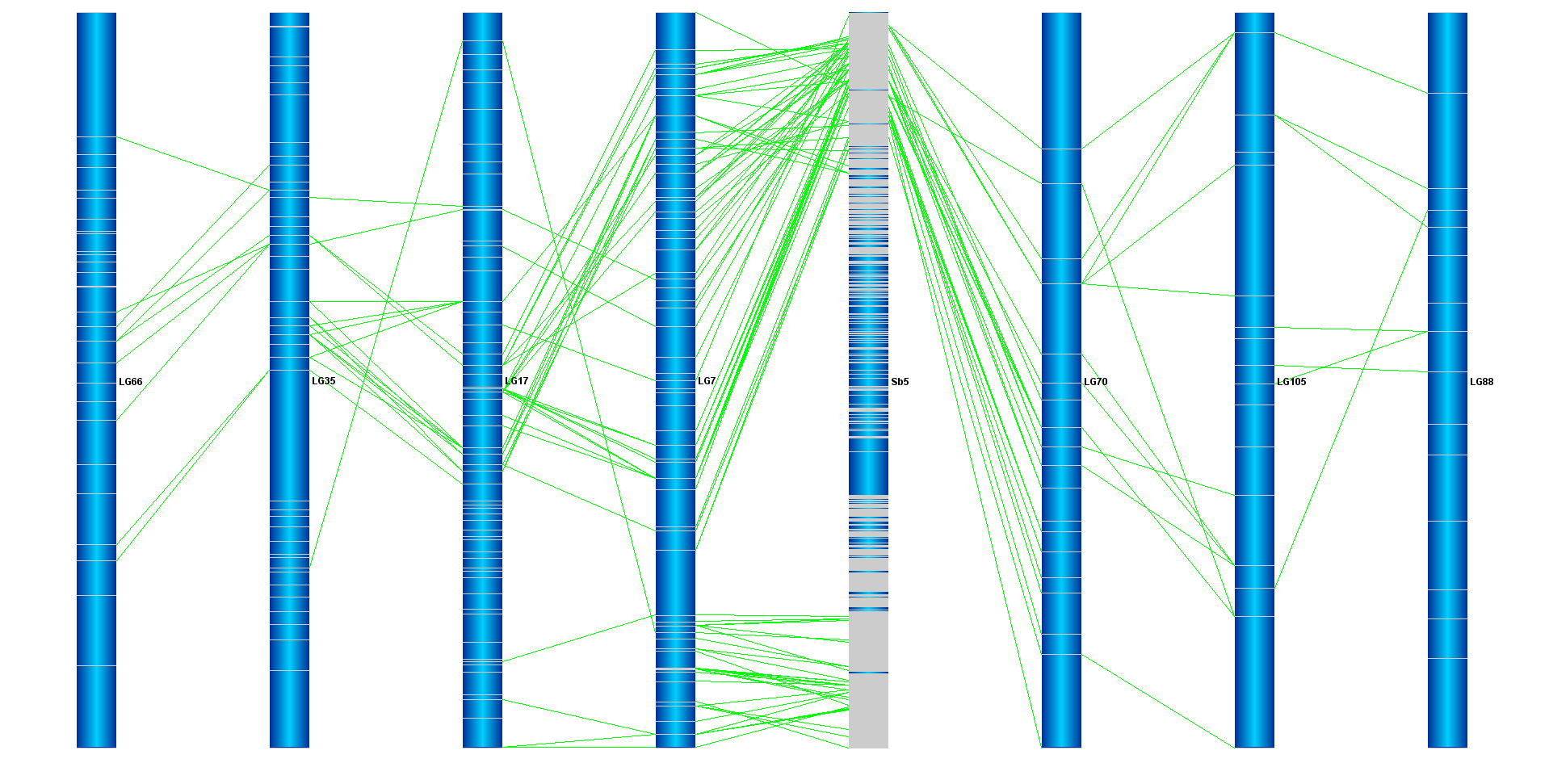

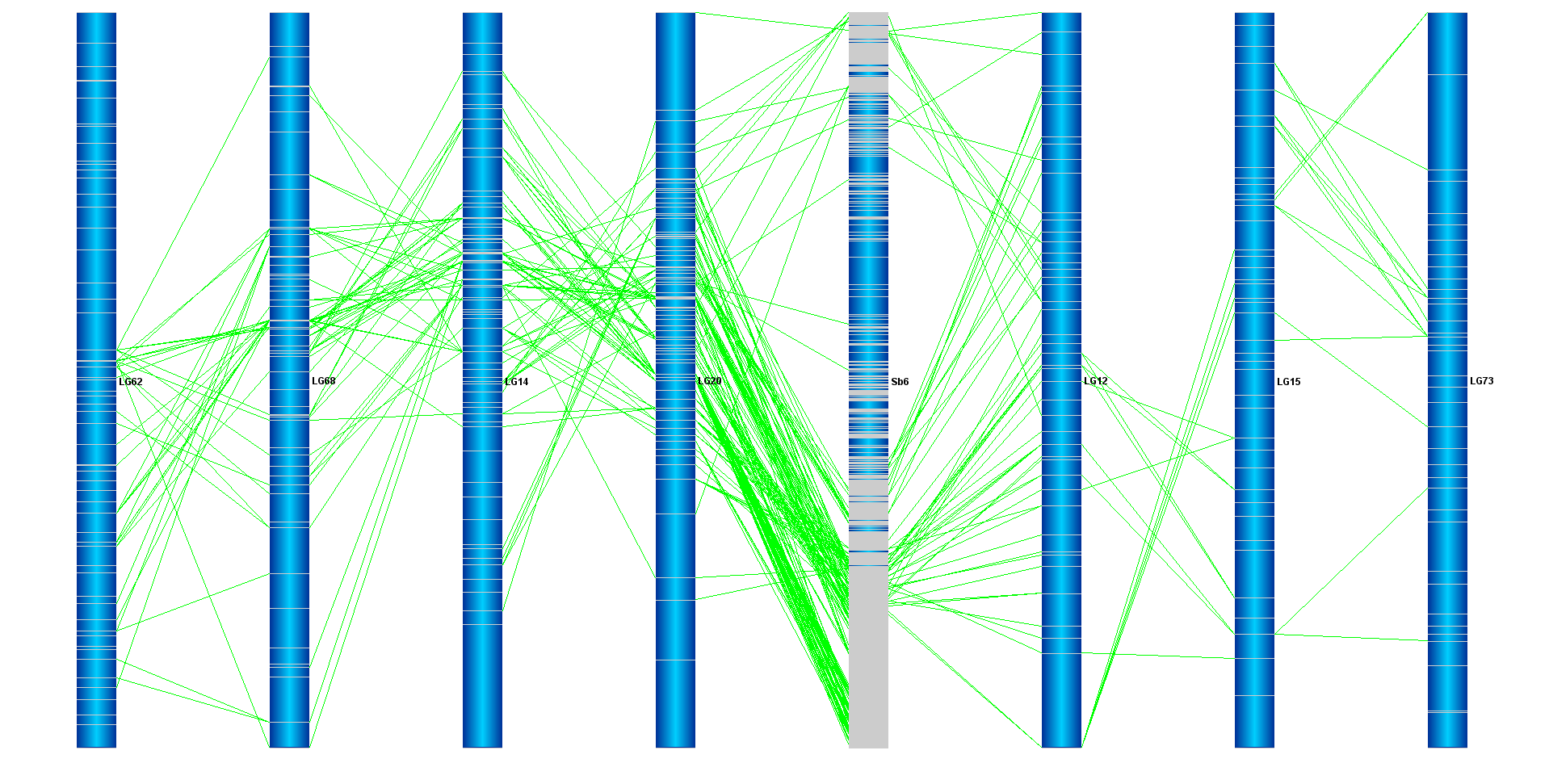

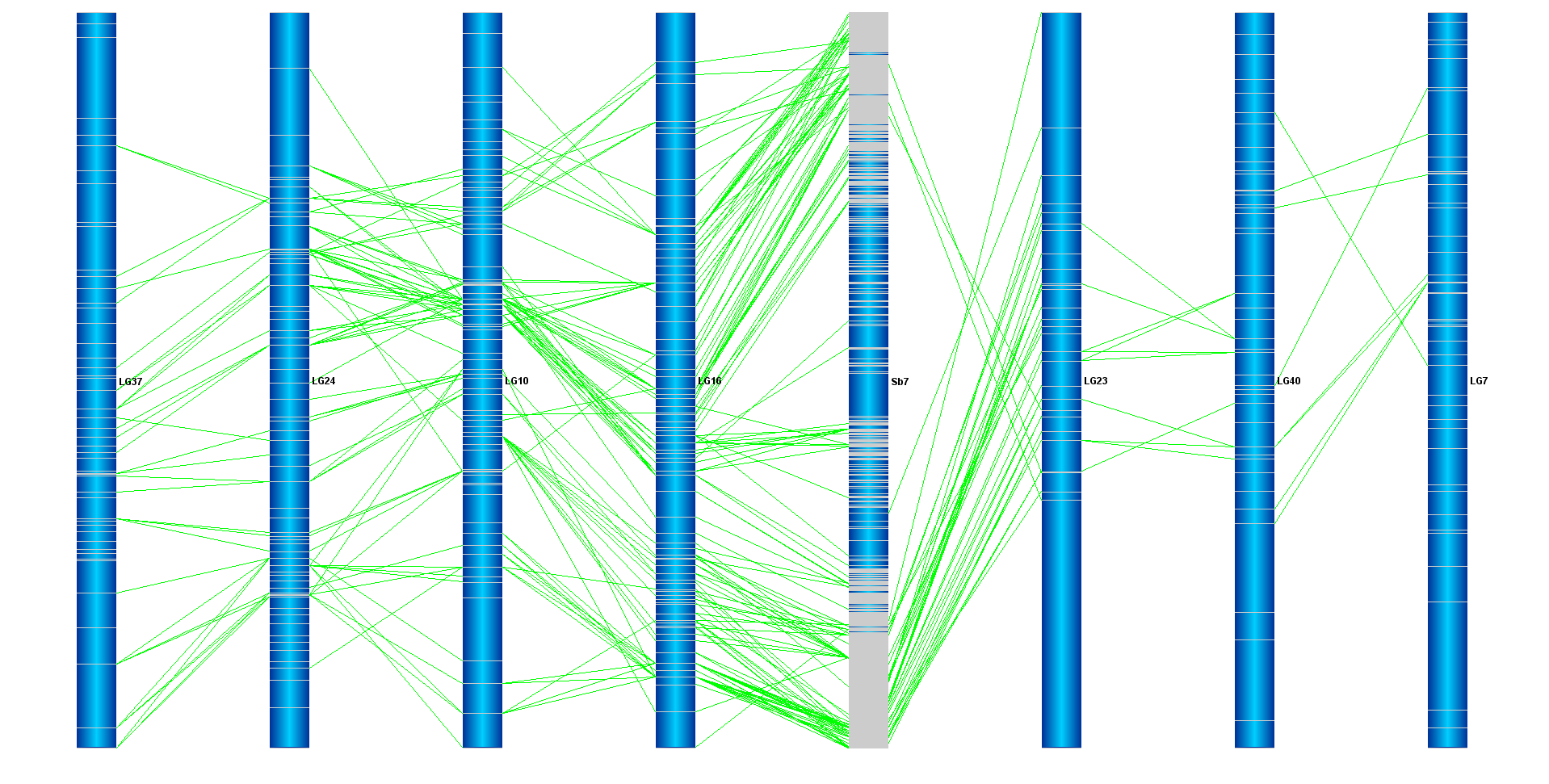

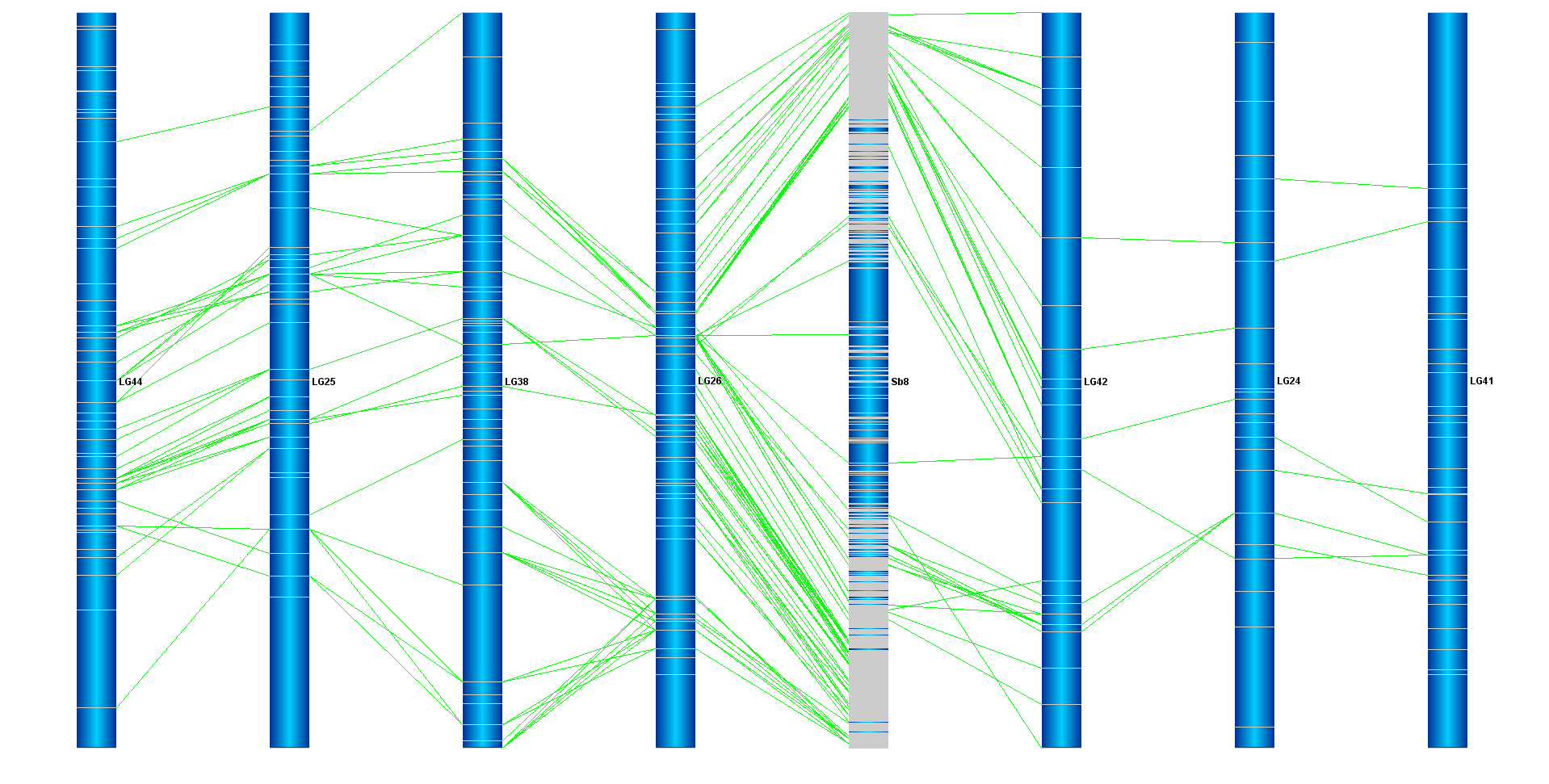

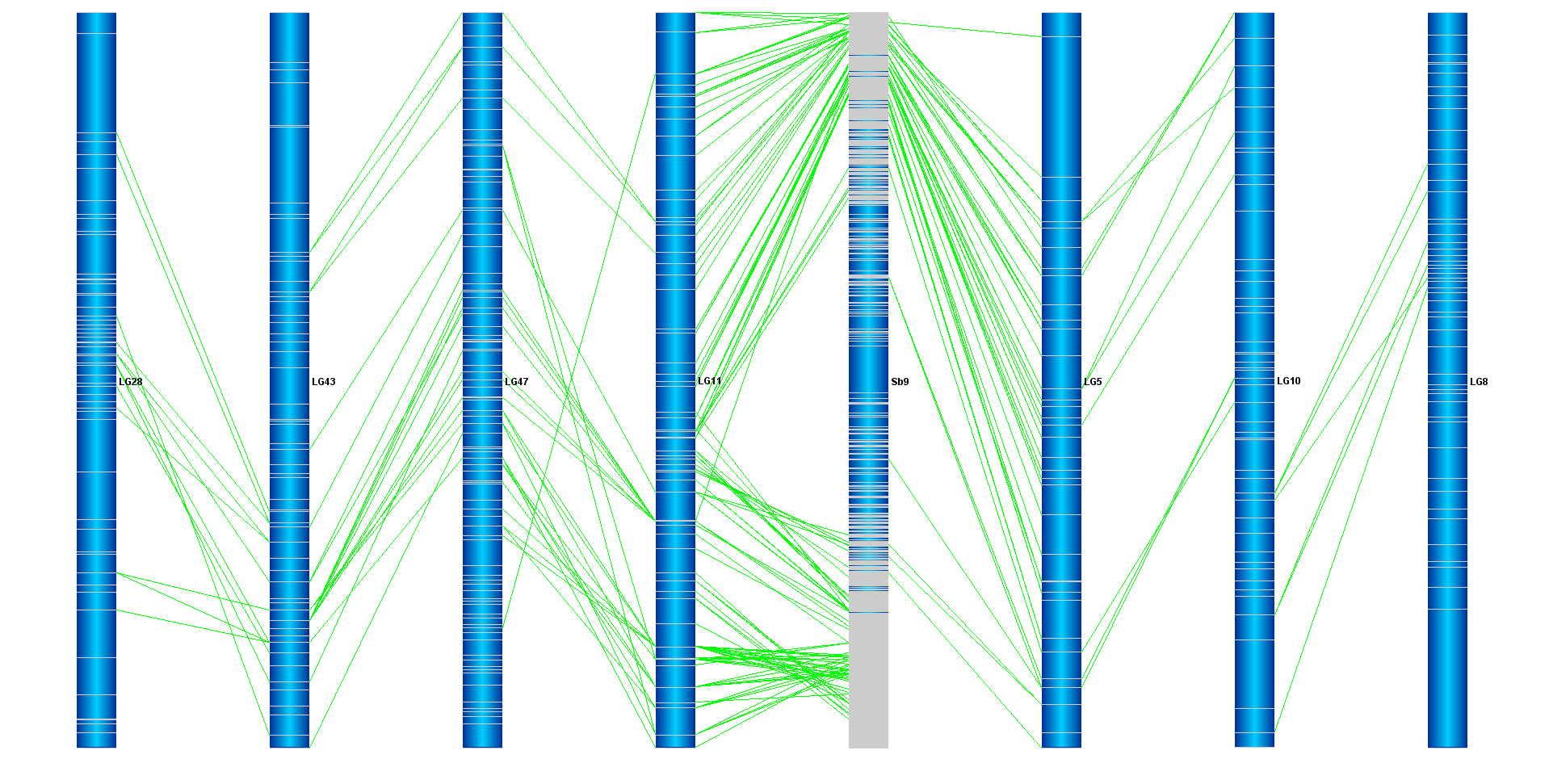

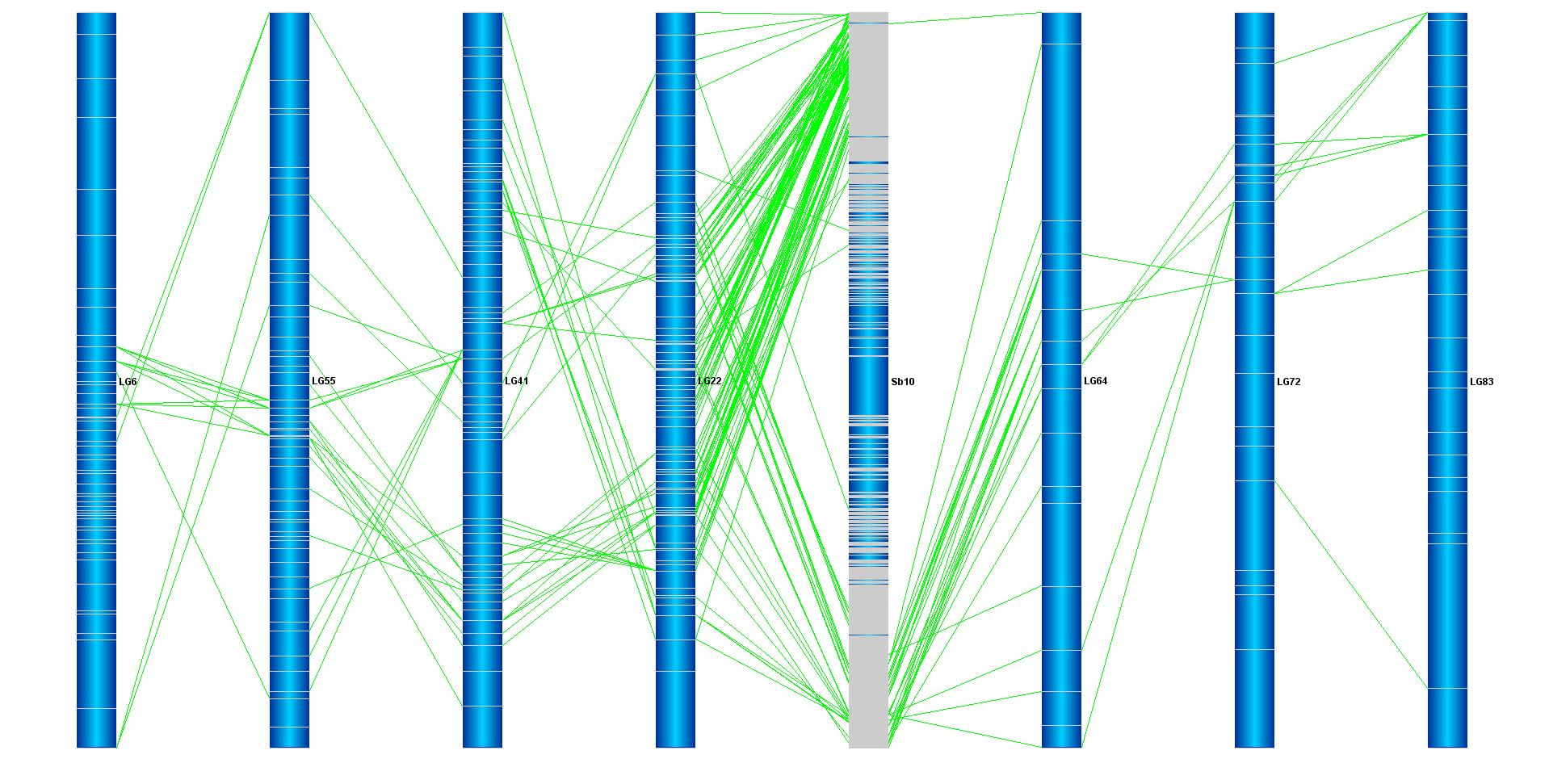


Supplemental Figure 9. Technology roadmap for this study


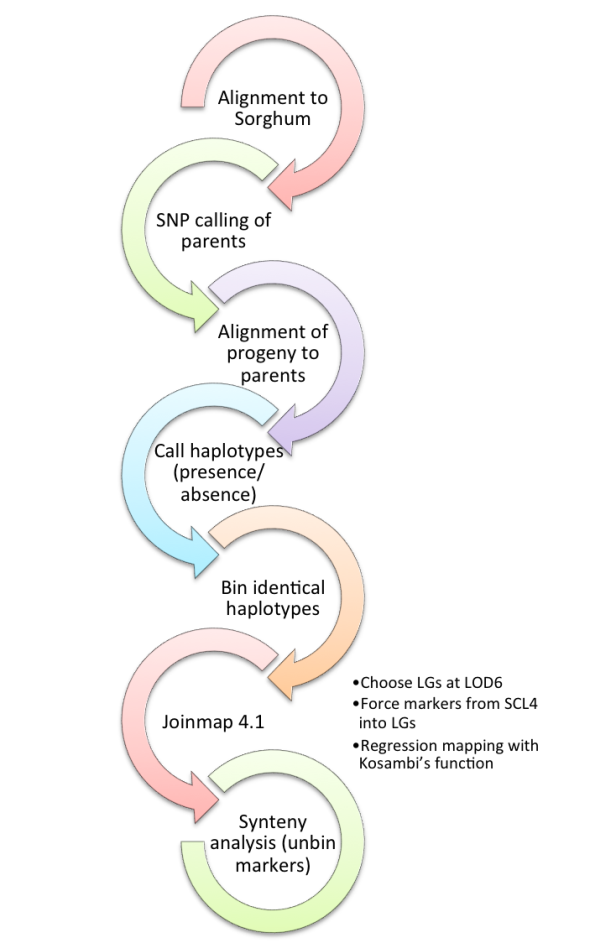

Supplement: Supplementary file 1 — Figure S1 The distribution of aligned reads within SPS gene among different libraries. Figure S2 Overview of single‐dose SNPs calling based on merged alignments RNA‐Seq libraries. Figure S3 (a and b) Distribution of single dose SNPs for parents based on qualified plant numbers and segregation ratios. (a) Distribution of single dose SNPs for female parent based on qualified plant numbers and segregation ratios. (b) Distribution of single dose SNPs for male parent based on qualified plant numbers and segregation ratios. Figure S4 The distribution of bin marker of LG in the sorghum genome. Figure S5 The collinearities between S. officinarum and S. robustum. Figure S6 Interchromosomal rearrangements between Saccharum and sorghum chromosomes. Figure S7 Intrachromosomal rearrangements between Saccharum and sorghum chromosomes. Figure S8 Intrachromosomal rearrangements of homologous group among Saccharum chromosomes. Figure S9 Technology roadmap for this study. [file PBI-17-264-s004.doc]
