# Supplementary material for: Recent polyploidization events in three Saccharum founding species
Source: Plant Biotechnol J. 2018 Jul 24;17(1):264–74. doi: 10.1111/pbi.12962 (PMC6330536; doi:10.1111/pbi.12962)
Supplement: Supplementary file 5 — Table S4 The conserved interchromosomal rearrangements of sorghum–S. officinarum and sorghum–S. robustum shared in S. officinarum and S. robustum. [file PBI-17-264-s001.docx]

| sorghum | *S. officinarum* | *S. robustum* |
| --- | --- | --- |
| Sb1+Sb5 | LG40 | LG27 |
|  | LG15 |  |
| Sb2+Sb3 | LG54 | LG36 |
| Sb4+Sb7 | LG3 | LG22 |
| Sb7+Sb9 | LG74 | LG7 |
| Sb1+Sb3 | LG4 | LG56 |
|  | LG23 |  |
|  | LG21 |  |
|  | LG12 |  |
| Sb7+Sb6 |  | LG7 |
| Sb10+Sb6 |  | LG14 |
| Sb3+Sb7 |  | LG34 |
| Sb3+Sb8 |  | LG6 |
| Sb3+Sb5 | LG39 |  |
|  | LG13 |  |
| Sb9+Sb2 | LG28 | LG8 |
|  | LG51 |  |
|  | LG2 |  |
| Sb9+Sb10 |  | LG31 |

**Supplemental Table** 4 The conserved interchromosomal rearrangements of sorghum-*S. officinarum* and sorghum-*S. robustum* shared in *S. officinarum* and *S. robustum*

Notes: The Sorghum chromosome which highlighted with red color contained majority of bin makers in the LG.
